# Activity-Based Photosensitizers with Optimized Triplet State Characteristics Toward Cancer Cell Selective and Image Guided Photodynamic Therapy

**DOI:** 10.1021/acsabm.2c00202

**Published:** 2022-05-10

**Authors:** Eda Kilic, Zubeyir Elmazoglu, Toghrul Almammadov, Dilay Kepil, Thibaud Etienne, Antoine Marion, Gorkem Gunbas, Safacan Kolemen

**Affiliations:** † Department of Chemistry, Koç University, 34450 Istanbul, Turkey; ‡ Department of Chemistry, Middle East Technical University (METU), 06800, Ankara, Turkey; § Université de Lorraine, CNRS, LPCT, F-54000 Nancy, France; ∥ Surface Science and Technology Center (KUYTAM), Koç University, 34450 Istanbul, Turkey; ⊥ Boron and Advanced Materials Application and Research Center, Koç University, 34450 Istanbul, Turkey; # TUPRAS Energy Center (KUTEM), Koç University, 34450 Istanbul, Turkey

**Keywords:** cancer, photodynamic therapy, activatable photosensitizer, DCM, cysteine

## Abstract

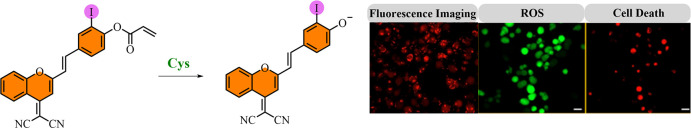

Activity-based theranostic photosensitizers are highly attractive in photodynamic therapy as they offer enhanced therapeutic outcome on cancer cells with an imaging opportunity at the same time. However, photosensitizers (PS) cores that can be easily converted to activity-based photosensitizers (aPSs) are still quite limited in the literature. In this study, we modified the dicyanomethylene-4*H*-chromene (DCM) core with a heavy iodine atom to get two different PSs (DCM_O_-I, I-DCM_O_-Cl) that can be further converted to aPS after simple modifications. The effect of iodine positioning on singlet oxygen generation capacity was also evaluated through computational studies. DCM_O_-I showed better performance in solution experiments and further proved to be a promising phototheranostic scaffold via cell culture studies. Later, a cysteine (Cys) activatable PS based on the DCM_O_-I core (DCM_O_-I-Cys) was developed, which induced selective photocytotoxicity along with a fluorescence turn-on response in Cys rich cancer cells.

## 1 Introduction

Photodynamic therapy (PDT) is a clinically approved and developing therapeutic modality for various types of tumors that utilizes light-activated photosensitizers (PS) and oxygen to eradicate cancer cells.^1^ During a typical PDT action, the combination of excitation light, triplet PS, and tissue oxygen results in cytotoxic reactive oxygen species (ROS) generation, particularly singlet oxygen (^1^O_2_), which triggers oxidative damage induced cell apoptosis and/or necrosis.^2−4^ PDT has attracted immense attention after its first discovery as it offers remarkable advantages compared to state-of-the-art treatment modalities such as lack of multidrug resistance, minimum invasiveness, opportunities for repeated applications, and immune system activation.^5^ On the other side, the need for external light irradiation and tissue oxygen are two main factors restricting its efficacy on deeply seated hypoxic tumors. PDT is known as an intrinsically local treatment as the excitation light, which initiates ROS formation, can be targeted acutely on to the tumor region. Unfortunately, off-target photosensitization on normal tissues still exists and cannot be fully eliminated. Therefore, PSs with enhanced cancer cell selectivity are in high demand to minimize the side effects both during and post-PDT action. To this end, activity-based PSs (aPSs) are highly attractive as they tend to stay inactive in normal cells even under light excitation and turn-on their photocytotoxicity after getting activated in cancer cells via tumor-associated inputs such as overexpressed enzymes, ROS, low pH, and biothiols.^6−8^ In this way, they differentiate between cancerous and healthy cells and consequently reduce any potential damage to nearby normal cells that would otherwise be destroyed during PDT with “always-on” conventional PSs. In the design of aPSs, several requirements need to be fulfilled including strong absorption at long wavelengths, high photostability, ease of modification, water solubility, and adequate fluorescence quantum yield to visualize the PS intracellular localization as well as the efficacy of the therapy.^7,8^ Although, there are many different PS cores available in the current literature, only a few of them can be easily converted to an activatable PS and hold the aforementioned characteristics at the same time.^7−9^ Thus, there is still a need for new PS cores to enlarge the activity-based PS arsenal.

In our efforts to develop easily accessible, activity-based theranostic PS scaffolds with unique properties, we have focused our attention on to the dicyanomethylene-4*H*-chromene (DCM) core, which is a long-known and well-established fluorophore.^10^ DCM-based fluorophores are mainly composed of three parts: an electron-acceptor group (A), which is the DCM core itself, a π-conjugated linker, and an electron-donor group (D), mostly the phenolate unit.^11^ They possess excellent optical properties such as a long emission wavelength, a large Stokes shift, and excellent photostability.^12^ Significantly, DCM derivatives can be utilized as an activity-based molecular sensor. To this end, the DCM core has previously been employed for the detection of a wide range of analytes, including various anions, biothiols, ROS, and enzymes.^13,14^ In a typical design approach, the phenolate unit is masked with a cleavable cage unit that can be removed by a disease specific biological input. Selective removal of the cage group activates the blocked intramolecular charge transfer process (ICT), which results in red-shifted absorption and emission signals, allowing both ratiometric and turn off-on type detection of the analyte of interest.^15,16^ Additionally, DCM derivatives have been used in different applications including organic photovoltaics and molecular logic gates.^17^ Recently, Li et al. introduced a series of modified DCM derivatives by replacing the central oxygen atom with selenium, which are further decorated with either two iodine or bromine at the ortho position of the phenolate unit, as activatable PSs.^18^ It was shown that presence of heavy selenium along with iodine/bromine atoms increased the ^1^O_2_ quantum yield dramatically due to enhanced spin–orbit coupling mediated ISC. They also designed alkaline phosphatase (ALP) and peroxynitrite (^−^ONOO) responsive PSs based on their most promising di-iodinated Se-substituted DCM core and successfully tested their photocytotoxicities in cancerous cell lines.^18^ However, the synthetically more accessible standard DCM core has never been demonstrated as an effective, theranostic PDT agent. In this study, we aimed to investigate the PDT potential of heavy atom incorporated oxygen-substituted DCM derivatives and synthesized two different PSs. Among them, the oxygen substituted DCM core (DCM_O_-I) with an *ortho*-iodinated phenolate showed the best performance and it was further converted to a cysteine (Cys) activatable PS (DCM_O_-I-Cys) to get a cancer cell selective theranostic PS.

## 2 Experimental Section

### 2.1 General

The instrumentation and photophysical and photochemical properties have been described in the Supporting Information.

### 2.2 Synthesis

Compounds **1**,^19^**2**,^20^ and **6**,^21^ DCM,^22^ and DCM-N^23^ were synthesized according to previous literature reports.

#### DCM_O_-I

Compound **1** (35 mg, 168 mmol) and compound **2** (40 mg, 168 mmol) were dissolved in 10 mL of toluene with piperidine (16 μL) and acetic acid (16 μL) under an argon atmosphere at room temperature. Then the mixture was refluxed for 16 h, and the solvent was evaporated *in vacuo*. The obtained crude product was purified by silica column chromatography with DCM to DCM/MeOH (50:1.5, v/v) to get DCMO-I as an orange solid (40 mg, 55% yield). ^1^H NMR (500 MHz, *d*_6_-DMSO) δ 11.09 (s, 1H), 8.79 (dd, *J* = 8.4, 1.5 Hz, 1H), 8.25 (d, *J* = 2.2 Hz, 1H), 7.98 (ddd, *J* = 8.6, 7.2, 1.5 Hz, 1H), 7.83 (dd, *J* = 8.5, 1.3 Hz, 1H), 7.71 (d, *J* = 16.0 Hz, 1H), 7.67 (dtt, *J* = 10.3, 4.4, 2.2 Hz, 2H), 7.42 (d, *J* = 15.9 Hz, 1H), 7.04 (s, 1H), 7.00 (d, *J* = 8.4 Hz, 1H). ^13^C NMR (126 MHz, *d*_6_-DMSO) δ 159.31, 159.01, 153.41, 152.50, 139.17, 137.99, 135.86, 130.78, 128.78, 126.60, 125.12, 119.48, 117.80, 117.64, 117.60, 116.44, 115.68, 106.64, 86.17, 60.02. Mass spectrometry (ESI positive ion mode for [M + H]^+^): calcd for C_20_H_11_IN_2_O_2_ 437.9865, found 438.9948.

#### DCM_O_-I-Cys

DCM_O_-I (40 mg, 91 mmol) and acryolyl chloride (34 mg, 365 mmol) in the presence of triethyl amine (25 mg, 250 mmol) were dissolved in 10 mL of dry ACN at room temperature for 0.5 h and the solvent was evaporated *in vacuo*. The obtained crude product was purified by silica column chromatography with DCM/hexane (70:30 v/v) to get DCM_O_-I-Cys as a dark orange solid (15 mg, 35% yield). ^1^H NMR (500 MHz, CDCl_3_) δ 8.92 (dd, *J* = 8.4, 1.5 Hz, 1H), 8.07 (d, *J* = 2.1 Hz, 1H), 7.76 (ddd, *J* = 8.6, 7.2, 1.5 Hz, 1H), 7.62–7.55 (m, 2H), 7.52 (s, 1H), 7.47 (ddd, *J* = 8.4, 7.1, 1.3 Hz, 1H), 7.24 (d, *J* = 8.4 Hz, 1H), 6.89 (s, 1H), 6.79 (dd, *J* = 15.9, 1.4 Hz, 1H), 6.73 (dd, *J* = 17.3, 1.2 Hz, 1H), 6.38 (dd, *J* = 17.3, 10.5 Hz, 1H), 6.13 (dd, *J* = 10.5, 1.1 Hz, 1H). ^13^C NMR (126 MHz, CDCl_3_) δ 163.33, 156.65, 152.67, 152.41, 152.27, 138.79, 138.52, 135.93, 134.85, 134.33, 134.05, 128.59, 127.28, 126.14, 125.88, 123.47, 120.08, 118.64, 117.80, 116.55, 115.45, 107.51, 91.19, 63.72, 0.01. Mass spectrometry (ESI positive ion mode for [M + H]^+^): calcd for C_23_H_13_IN_2_O_3_ 491.9971, found 493.0042.

#### Compound **3**

2-Hydroxyacetophenone (2.2 g, 16.6 mmol) and *p*-toluenesulfonic acid (2.8 g, 16.6 mmol) were dissolved in 10 mL of ACN. After 5 min of stirring, *N*-iodosuccinimide (3.7 g, 16.6 mmol) was added to the solution portionwise at room temperature. The solution was allowed to stir overnight at 40 °C. Reaction was cooled down to room temperature, and ACN was removed under reduced pressure. The crude was diluted with 150 mL EtOAc and the mixture was washed with saturated sodium thiosulfate solution. Organic layer was separated, dried over Na_2_SO_4_ and the solvent was evaporated under reduced pressure. The crude product was purified by column chromatography on silica gel hexane/EtOAc (85:15 v/v) to give compound **3** as a yellowish solid (4.1 g, 94% yield). ^1^H NMR (500 MHz, CDCl_3_) δ 12.11 (s, 1H), 7.93 (s, 1H), 7.62 (d, *J* = 8.9 Hz, 1H), 6.69 (s, 1H), 2.56 (s, 3H). ^13^C NMR (126 MHz, CDCl_3_) δ 202.44, 160.92, 143.71, 138.17, 120.74, 119.90, 78.82, 25.48.

#### Compound **4**

To a solution of compound **3** (4.1 g, 15.6 mmol) in dry ethyl acetate (80 mL) was added sodium (5 g) as small pieces. The suspension was stirred overnight at room temperature. The solution was filtered, and the brown solid was dissolved in 100 mL of distilled water. Acidification of the solution with 1 N HCl was done to adjust the pH to neutral. The solution was washed with 250 mL of EtOAc, amd the organic layers were dried over Na_2_SO_4_, filtered, and concentrated to give a crude product as a brown solid which was directly used in the next reaction without further purification. Sulfuric acid (2.5 mL)was slowly added to a solution of the crude product in acetic acid (30 mL). The mixture was heated to 120 °C for about 0.5 h. After cooling to room temperature, the solution was poured into an ice bath and the pH was adjusted to neutral with the slow addition of Na_2_CO_3_ and dilute NaOH. The solution was washed with DCM (250 mL), and the organic layers were dried over Na_2_SO_4_, filtered, and evaporated to dryness. The residue was purified on silica gel with hexane/EtOAc (90:10 v/v) as an eluent to get compound **4** as a brown solid (1.6 g, 57% yield). ^1^H NMR (500 MHz, CDCl_3_) δ 9.14 (d, *J* = 2.0 Hz, 1H), 7.90 (dd, *J* = 8.8, 2.0 Hz, 1H), 7.13 (d, *J* = 8.8 Hz, 1H), 6.64 (d, *J* = 0.9 Hz, 1H), 2.35 (s, 3H). ^1^H NMR (500 MHz, CDCl_3_) δ 8.47 (dt, *J* = 7.3, 2.2 Hz, 1H), 7.88 (m, *J* = 8.7, 6.1, 2.0 Hz, 1H), 7.22–7.14 (m, 1H), 6.21–6.13 (s, 1H), 2.39 (s, 3H). ^13^C NMR (126 MHz, CDCl_3_) δ 176.62, 166.50, 155.89, 141.99, 134.57, 125.22, 119.91, 110.71, 88.75, 20.52.

#### Compound **5**

Compound **4** (50 mg, 0.001 mmol) and malononitrile (57 mg, 12.87 mmol) were dissolved in 2.5 mL of acetic anhydride, the solution was refluxed overnight. After completion, 10 mL of methanol was added to the solution and refluxed for another 3 h. The solvent was evaporated *in vacuo*, and the residue was purified on silica gel with hexane/EtOAc (80:20 v/v) as an eluent to get compound **5** as a dark-reddish solid (30 mg, 51% yield). ^1^H NMR (500 MHz, CDCl_3_) δ 9.14 (d, *J* = 2.0 Hz, 1H), 7.90 (dd, *J* = 8.8, 2.0 Hz, 1H), 7.13 (d, *J* = 8.8 Hz, 1H), 6.64 (d, *J* = 0.9 Hz, 1H), 2.35 (s, 4H).^13^C NMR (126 MHz, CDCl_3_) δ 160.56, 151.40, 150.13, 142.21, 133.28, 119.32, 118.41, 114.92, 113.94, 104.59, 88.42, 62.45, 19.43. Mass spectrometry (ESI negative ion mode for [M – H]^−^): calcd for C_13_H_7_IN_2_O 336.9603, found 336.9600

#### I-DCM_O_-Cl

I-DCM_O_-Cl was synthesized according to the same procedure as of DCMo-I. ^1^H NMR (500 MHz, *d*_6_-DMSO) δ 9.10 (d, *J* = 2.0 Hz, 1H), 8.27 (dd, *J* = 8.9 Hz, 1H), 7.91 (d, 1H), 7.73 (d, *J* = 15.9 Hz, 1H), 7.64 (d, 1H), 7.59 (dd, 1H), 7.44 (d, *J* = 15.9 Hz, 1H), 7.09 (d, *J* = 8.5 Hz, 1H), 7.03 (s, 1H).^13^C NMR (126 MHz, *d*_6_-DMSO) δ 158.89, 152.17, 151.67, 143.78, 138.50, 133.39, 130.03, 129.52, 127.75, 121.53, 121.24, 119.73, 117.52, 116.15, 106.83, 90.83. Mass spectrometry (ESI negative ion mode for [M – H ]^−^): calcd for C_20_H_10_IN_2_O_2_Cl 471.9475, found 470.9417

### 2.3 *In Vitro* Experiments

#### 2.3.1 Cell Culture and Treatments

Human cervical cancer cells (HeLa) and mouse fibroblast cells (L929) were cultured in DMEM high glucose supplemented with 10% heat inactivated fetal bovine serum (FBS),1% penicilin/stremptomycin, 0.5% amphothericin B, and 2 mM glutamine in a humidified atmosphere at 37 °C with 5% CO_2_. Cells were subcultured at 80–90% confluency every 2–3 days. For photodynamic therapy, cells were treated with various concentrations (0.02–10 μM) of DCM_O_-I or DCM_O_-I-Cys for 1 or 2 h in the dark, followed by LED light (595 nm, 9.83 mW/cm^2^) illumination for 2 h (with fresh media), then kept in an incubator up to 24 h in the dark. For the dark toxicity, cells were treated under the same conditions without any LED illumination and incubated up to 24 h, and then the cell viability analysis was held.

#### 2.3.2 Cell Viability Assay

Cells (3 × 10^4^/well) were seeded on 96 well plates and incubated for 24 h. They were then treated with DCM_O_-I or DCM_O_-I-Cys according to the PDT protocol. After incubation periods, the cell media was removed and 100 μL fresh medium containing MTT (0.5 mg/mL) was added to each well, then incubated at 37 °C for 2–4 h. After the incubation period, formazan crystals were dissolved with equal volumes of 10% SDS in PBS (0.01 N HCl) at 37 °C, overnight. The absorbance of each well was measured at a wavelength of 490 and 570 nm using a microplate reader (MultiskanSky, Thermo Scientific, USA) (*n* = 6).

#### 2.3.3 Cellular Internalization Assay

Cells were seeded on 35 mm glass bottom confocal dishes (1 × 10^4^) and then treated with DCM_O_-I or DCM_O_-I-Cys (2 μM) for 30 min, 1 h, or 2 h at 37 °C. After the incubation period, cells were washed three times with 1× PBS and fixed with 4% paraformaldehyde for 15–20 min at RT. After three washes with 1× PBS, cells nuclei were stained with Hoechst 33342 (2 μg/mL in PBS) and confocal images were taken at 361/497 nm (ex/em) and 550/680–704 (ex/em) wavelengths for Hoechst and DCM_O_-I or -Cys by Zeiss LSM 900 CLSM, respectively (40×) (*n* = 3).

#### 2.3.4 ROS Generation Assay

Cells were treated with the IC_50_ values of DCM_O_-I or DCM_O_-I-Cys for 2 h in the dark, illuminated with LED light for 2 h in the presence or absence of increasing concentrations of scavengers for ROS (*N*-acetylcysteine, NAC) or singlet oxygen (sodium azide, NaN_3_), and then incubated up to 24 h in the dark for MTT analysis. For confocal imaging, cells were seeded at a density of 2 × 10^4^ cells/well on 96 well plates and treated with DCM_O_-I or DCM_O_-I-Cys for 2 h in the dark and illuminated with LED in the presence or absence of NAC or NaN_3_ for 2 h. After illumination, cells were washed twice with 1× PBS and then stained with DCFH-DA (20 μM), PI (10 μg/mL), and Hoechst 33342 (2 μg/mL) in serum free media for 30–45 min. After the washing steps with 1× PBS, confocal images were taken at 488/535 nm (ex/em), 550/617 nm (ex/em), and 361/497 nm (ex/em) wavelengths for DCF, PI, and Hoechst by Zeiss LSM 900 CLSM, respectively (Carl Zeiss, Oberkochen, Germany). Six images of duplicate wells were taken for each experiment (10×).

#### 2.3.5 AO/EtBr Staining for Apoptosis and Necrosis

Cells were seeded at a density of 2 × 10^4^ cells/well on black walled, clear bottom 96 well plates and treated with DCM_O_-I or DCM_O_-I-Cys and an additional 2 h w/wo LED illumination. After the illumination period, cells were kept at 37 °C for 30 min and washed twice with 1× PBS. Equal volumes of AO (1 μg/mL) and EtBr (1 μg/mL) in serum free media were added after the last wash and incubated for 30 min at 37 °C. Cells were washed with 1× PBS and images were taken at 500/525 (ex/em) and 530/617 nm (ex/em) wavelengths by a Zeiss LSM 900 CLSM (Carl Zeiss, Oberkochen, Germany). Six images of duplicate wells were taken for each experiment (10×).

#### 2.3.6 Statistical Analysis

MTT results were expressed as the percentages of DMSO treated vehicle controls. IC_50_ values were calculated by the concentration-normalized response curves obtained from nonlinear regression analysis. One-way analysis of variance (ANOVA) with Tukey post hoc tests were performed using GraphPad Prism 8.0 software for viability and scavenger assays. Data were expressed as mean ± SD, and values of *p* < 0.05, *p* < 0.01, and *p* < 0.001 were considered as statistically significant.

### 2.4 Theoretical Calculations

All calculations were performed with Orca5.0.2,^24^ with additional single points done with Gaussian 16^25^ to generate data files compatible with the Mesra software^26^ for analysis of the nature of electronic transitions. Geometry optimizations were systematically followed by frequency calculations, either analytical or numerical, to characterize the corresponding stationary points, except for the benchmark. Excited states were calculated with time-dependent density-functional theory (TD-DFT). The Tamm–Dancoff approximation was used only for geometry optimization of excited states and switched off for energy and properties calculations. The benchmark was conducted on DCM_o_-I to determine the impact of varying the density functionals, basis set, and solvent. Solvent effects were introduced in an implicit manner via the CPCM solvation model with the dielectric constant set to emulate a DMSO environment. We selected two GGA functionals (i.e., BP86^27,28^ and PBE^29^), two meta-GGA (i.e., TPSS^30^ and M06L),^31^ two hybrids (i.e., B3LYP^27,32−34^ and TPSSH),^30,35^ two range-separated hybrids (i.e., CAM-B3LYP^29,36^ and ωB97X-D3BJ^37,38^), two range-separated double-hybrids (i.e., RSX-0DH^39^ and ωPBEPP86),^40^ and three range-separated double-hybrids with spin-component or spin-opposite scaling (i.e., ωB97X-2^41^ and SCS/SOC-ωPBEPP86^40^). D3BJ dispersion corrections were applied when relevant and available in Orca.^42,43^ Because of the presence of iodine in the molecules under investigation, relativistic effects were systematically included in all calculations using the Douglas–Kroll–Hess (DKH) Hamiltonian^44−46^ and related basis sets. Relativistic basis were set on iodine and chlorine (for consistency) only with the corresponding DKH version of the correlation-consistent basis used for lighter elements (i.e., [aug]-cc-pVnZ-DK, n = D,T). For the benchmark, five basis sets were considered: cc-pVDZ/cc-pVTZ-DK(iodine), cc-pVTZ/cc-pVTZ-DK(iodine), aug-cc-pVDZ/aug-cc-pVTZ-DK(iodine), aug-cc-pVTZ/aug-cc-pVTZ-DK(iodine), and aug-cc-pVDZ/cc-pVTZ-DK(iodine). For single points using Gaussian 16, the cc-pVTZ-DK basis set was obtained via ccRepo.^47^ Geometry optimizations for the benchmark were conducted with the corresponding density functional and the cc-pVDZ/cc-pVTZ-DK(iodine) in the gas phase. As discussed in the results section, B3LYP yielded results in fair agreement with experiments, especially for the difference in energy between excited states. We thus decided to select B3LYP/cc-pVDZ/cc-pVTZ-DK(iodine)/CPCM(DMSO) for geometry optimizations and B3LYP/aug-cc-pVDZ/cc-pVTZ-DK(iodine)/CPCM(DMSO) for excited states and properties calculations. For DCM_o_-I, ensembles of structures in the ground state and first singlet state were generated as a Wigner distribution of 1000 conformations at 300 K with the tools available in the SHARC software.^48−50^ A locally modified version of the Mesra software was used in combination with Gaussian 16 outputs to calculate detachment/attachment densities between states of interest and related descriptors. Natural transition orbitals (NTOs) were computed with Theodore,^51^ and JMol was used to generate plots shown in the Supporting Information. Spin orbit coupling matrix elements were calculated with Orca5.0.2, and the magnitude of the spin orbit coupling was computed as the square root of the square modulus over real and imaginary parts of *x*, *y*, and *z* components. All molecules were modeled in their deprotonated form (i.e., phenolate). Electronic structure analysis and spin orbit coupling matrices are available in the Supporting Information for all molecules discussed in this work.

## 3 Results and Discussion

**Figure 1:**
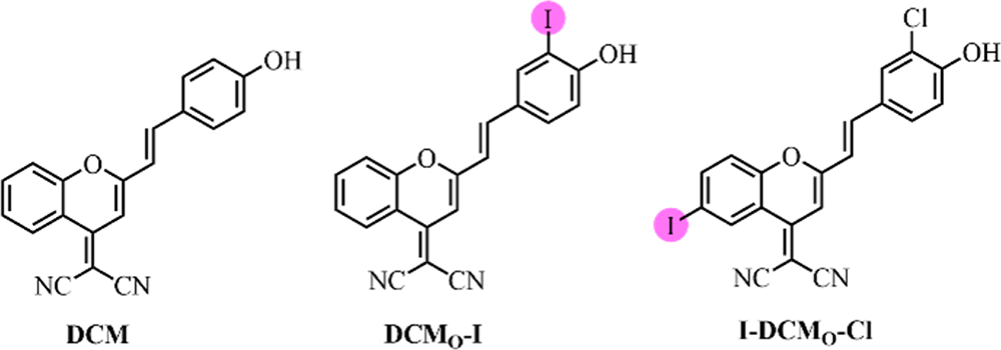
Structures of DCM and photosensitizers DCM_O_-I and I-DCM_O_-Cl.

In the design of DCM-based PSs (Figure 1), we concentrated on oxygen-substituted DCMs (DCM_O_) and introduced DCM_O_-I, which bears a monoiodo substituted phenolate unit. Iodine triggers ISC and decreases the p*K*_a_ of the phenol, which consequently increases the phenolate concentration in physiological medium (pH 7.4) as previously noted.^52^ In order to evaluate the influence of iodine location on efficiency of ISC, we moved the iodine from the phenolate ring to the DCM core (I-DCM_O_-Cl) and introduced an ortho chlorine to decrease the p*K*_a_ of the phenol as in the case of DCM_O_-I.

**Scheme 1:**
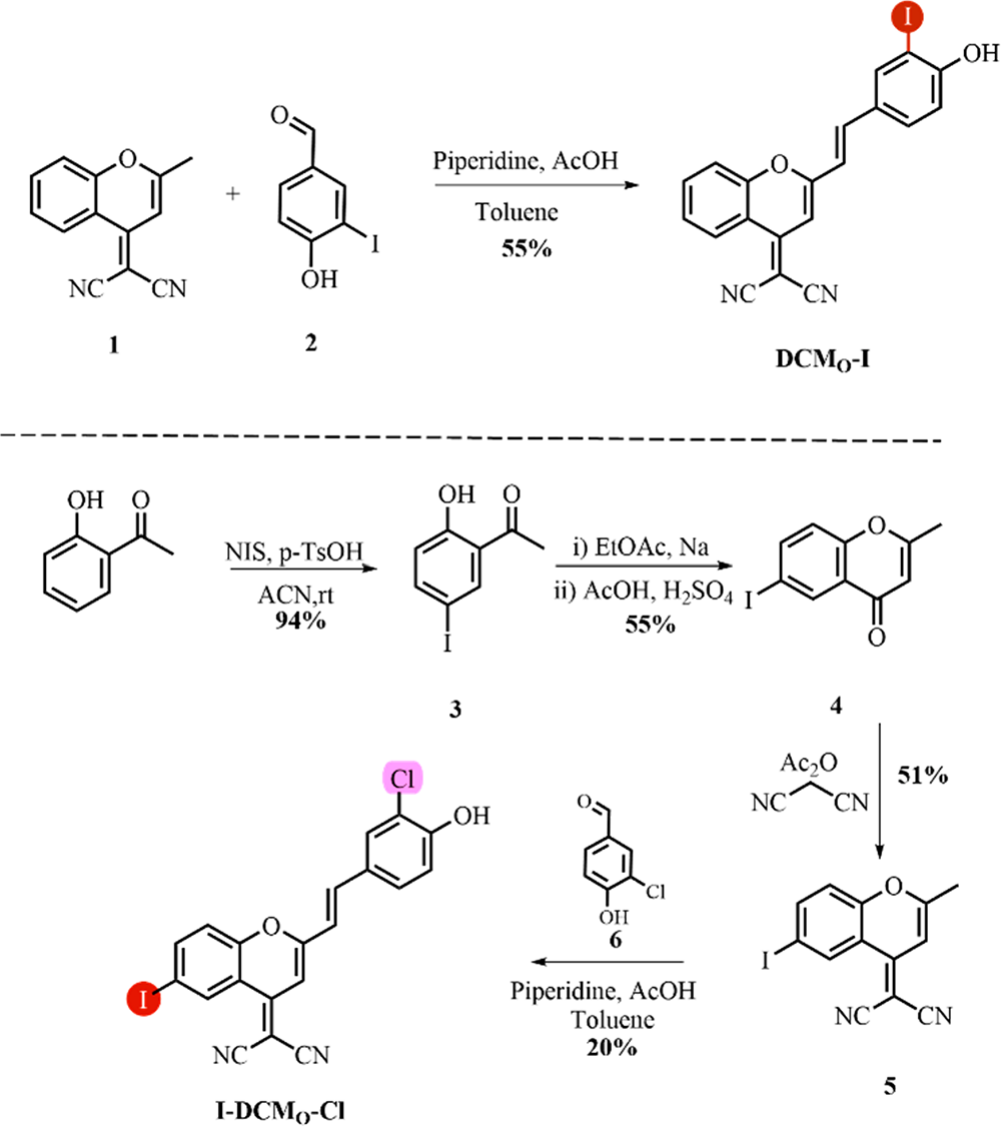
Synthetic Route of DCM_O_-I and I-DCM_O_-Cl

Synthetic pathways for proposed PSs are depicted in Scheme 1. DCM_O_-I was synthesized by reacting the DCM core (**1**) with a 4-hydroxy-3-iodobenzaldehyde (**2**) under reflux conditions through a Knoevenagel reaction. For the synthesis of I-DCM_O_-Cl, an iodinated DCM core was initially prepared. In this direction, 2-hydroxyacetophenone was iodinated by NIS to give compound **3**, which was followed by chromone ring formation (**4**). Later, the reaction with iodinated chromone (**4**) and malononitrile afforded an iodinated DCM scaffold (**5**). Finally, condensation of **5** with 4-hydroxy-3-chlorobenzaldehyde (**6**) yielded I-DCM_O_-Cl.

**Figure 2:**
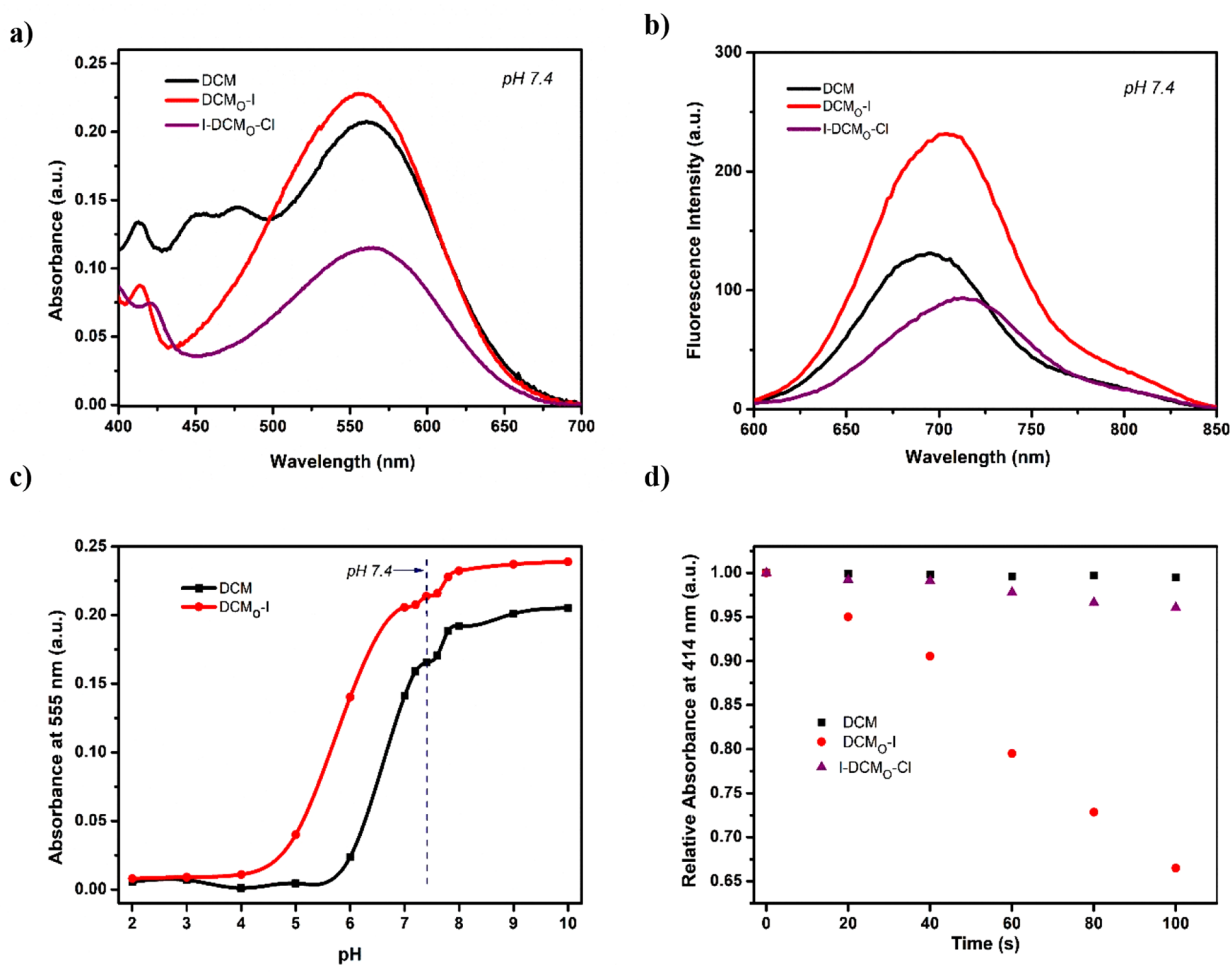
(a) Absorption and (b) fluorescence spectra of DCM, DCM_O_-I, and I-DCM_O_-Cl. (c) pH-dependent absorption signal of DCM_O_-I and DCM at 555 nm in aqueous solutions. (d) Decrease in the absorption signal of DPBF at 414 nm upon irradiating DCM, DCM_O_-I, or I-DCM_O_-Cl containing DMSO (1% PBS, pH 7.4) solutions with a 630 nm LED (24.3 mW/cm^2^) light.

^1^O_2_ generation performances of the PSs were investigated first chemically in DMSO (1% PBS, pH 7.4) solutions by using a well-established trap molecule, 1,4-diphenylbenzofuran (DPBF). Irradiation of PSs with a 630 nm LED (24.3 mW/cm^2^) caused a remarkable decrease in the DPBF absorption, suggesting photosensitized ^1^O_2_ formation in each case (Figure 2d and Figures S3 and S4c,d). Singlet oxygen quantum yields of agents were calculated by using methylene blue as a standard PS^53^ (Figure S5, Table S1). DCM_O_-I, which bears an iodine on the phenolate ring, exhibited the highest yield (Φ_Δ_ = 5.2%). Interestingly, when the iodine was placed on the chromone ring of the DCM core (I-DCM_O_-Cl), the ^1^O_2_ generation yield (Φ_Δ_ = 0.6%) (Figure S6) dropped dramatically, suggesting that the location of the heavy atom on DCM based PSs is highly critical. The DCM core itself did not show any sign of ^1^O_2_ generation under 630 nm LED irradiation as expected (Figure S4a,b).

After completing the synthesis, we first evaluated the photophysical properties of DCM-based PSs in aqueous solutions (PBS, pH 7.4, 50% DMSO) (Table S1). Heavy atom bearing oxygen substituted PSs DCM_O_-I and I-DCM_O_-Cl exhibited absorption peaks at 555 and 560 nm, respectively, which is similar to the parent DCM core (λ_abs_ = 560 nm) (Figure 2a). In the case of fluorescence measurements, DCM_O_-I and I-DCM_O_-Cl gave signals at 728 and 738 nm, respectively, which are red-shifted compared to the DCM scaffold (λ_em_ = 705 nm) (Figure 2b). DCM_O_-I possessed a higher fluorescence quantum yield (Φ_F_ = 0.24), however significantly lower compared to DCM^10^ as expected, since ISC and fluorescence are two competing pathways. Both agents showed similar pH dependency as evidenced from their absorption and emission signals in aqueous solutions that were buffered at different pHs (Figure 2c, Figures S1 and S2). At physiological pH (7.4), they were almost fully deprotonated, staying as phenolate anions, and reached their maximum fluorescence intensities. This is quite promising considering the potential bioapplications.

**Figure 3:**
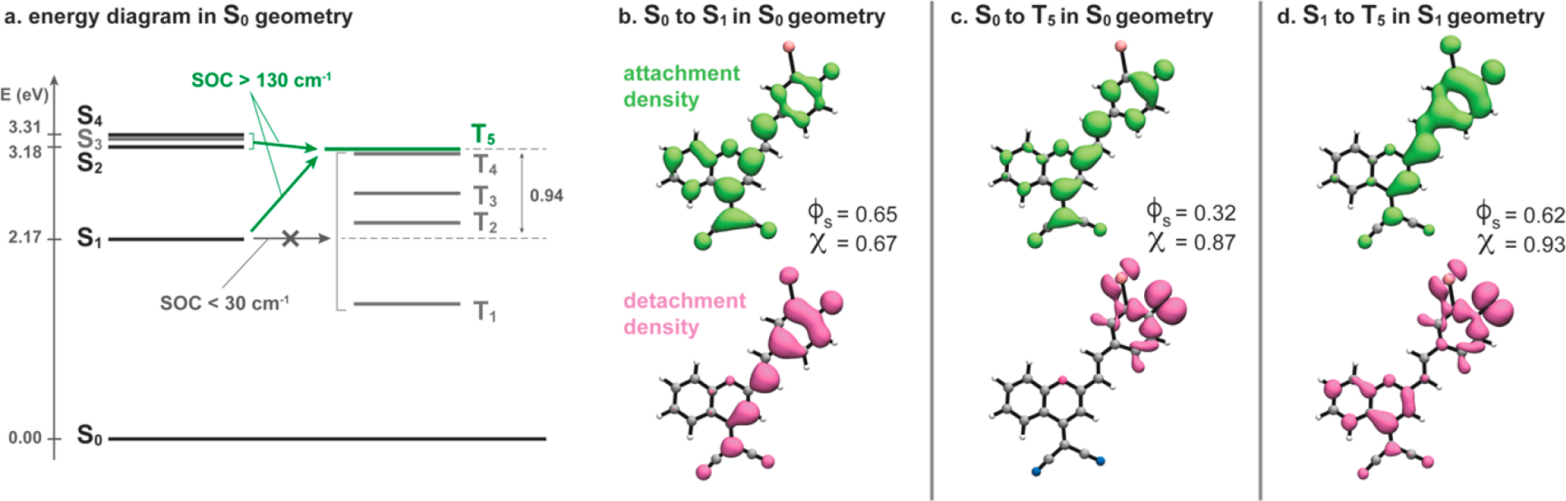
Energy diagram and electronic transition nature for DCM_O_-I at the B3LYP/aug-cc-pVDZ/cc-pVTZ-DK(iodine)/CPCM(DMSO) level. (a) Energy diagram in the minimum geometry of S0. S1, S2, and S4 are coupled with T5 with a magnitude of spin orbit coupling of 175, 136, and 208 cm^–1^, respectively. (b) Detachment and attachment electron densities involved in the transition from S0 to S1 in the geometry of S0. (c) Same as part b showing the effective electron density reorganization to reach T5 from S0 (via any intermediate singlet state). (d) Same as part b showing the electron density reorganization upon transition from S1 to T5, in the geometry of S1. All surfaces are plotted with an isodensity value of 0.001. Detachment/attachment densities overlap integral (ϕ_s_) and magnitude of the net transferred charge upon electron density reorganization (*q*^CT^) are given for each transition.

To further understand the mechanism of ISC in different DCM-based PSs, we conducted a computational study at the time-dependent density-functional theory level. We first determined the method of choice by performing an extensive benchmark of functionals, basis sets, and solvation, which we reported in the Supporting Information (see the supplementary text, Tables S4–S6, and Figure S13). We then analyzed the electronic structure of DCM_O_-I with a particular interest in the mechanism underlying the intersystem crossing phenomenon that populates the triplet states of the dye. Our results were summarized in Figure 3. We found that only one triplet state, T5, is significantly coupled to S1 with a total magnitude of the spin orbit coupling of 175 cm^–1^. The difference in energy between S1 and T5 in the geometry of S0 is, however, substantial (0.94 eV). T4 is found slightly lower than T5 and also couples with S1, yet with a significantly lower coupling constant of 26 cm^–1^. Lower triplet states have a nearly null coupling to S1. As a comparison, Zobel et al.^54^ as well as Orozco-Gonzalez et al.^55^ discuss spin orbit couplings of 40–65 cm^–1^ in the intersystem crossing mechanism of 2-nitronaphthalene. The spin orbit coupling between S1 and T5 calculated for DCM_o_-I (i.e, 175 cm^–1^) is therefore likely to induce intersystem crossing, yet the high and positive energy gap between the states is prohibitive. Relaxing the structure to the geometry of S1 yields a similar picture with a spin orbit coupling of 184 cm^–1^ and an energy gap of 1.07 eV.

**Figure 4:**
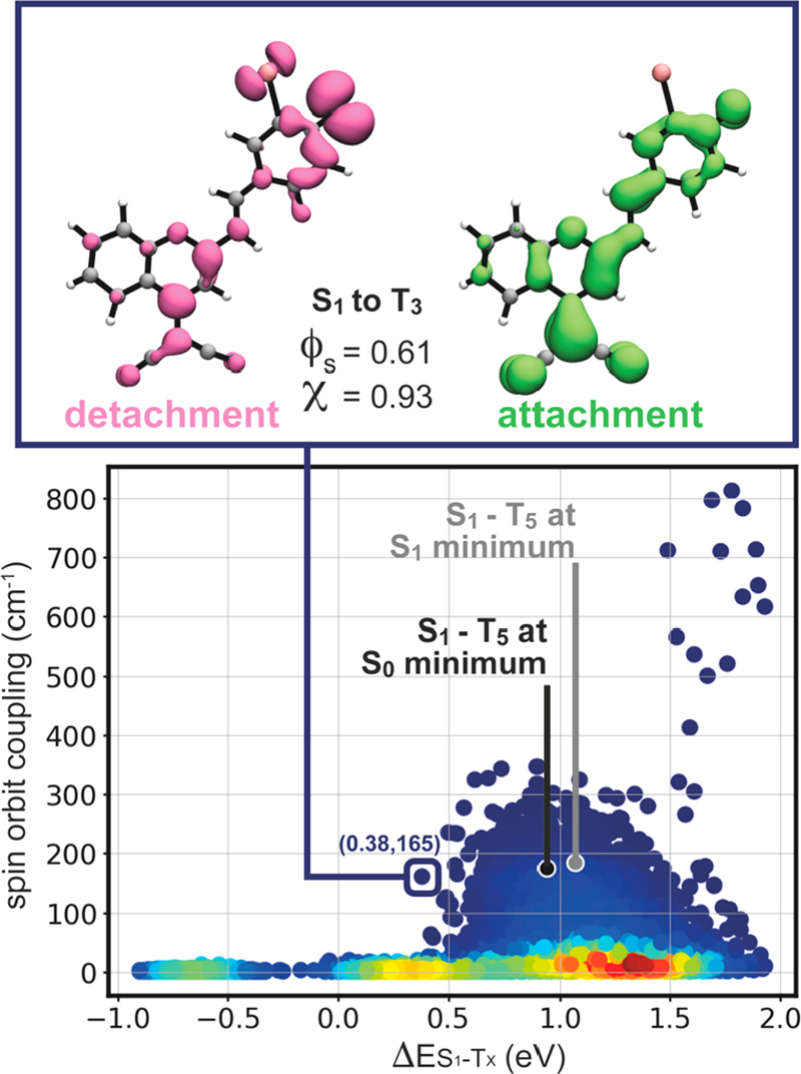
Representation of the energy difference between S1 and T1–10 versus the corresponding magnitude of the spin orbit coupling for a Wigner distribution at 300 K based on the normal modes in the S1 minimum geometry of DCM_O_-I. The S1–T5 energy difference and coupling in the geometry of S0 and S1 are represented in the figure as black and gray dots, respectively. The color scale reflects the density of points with blue-green-yellow-red ranging from low to high density. One point of the distribution is highlighted, and the topology of the corresponding transition is represented by its detachment/attachment density plot in the upper panel.

A comparison of the electron density between different states of DCM_O_-I was represented in Figure 3b–d with detachment/attachment electron densities and related descriptors as calculated with a locally modified version of the Mesra software.^26^ In brief, the detachment density corresponds to the portion of electron density that is displaced from the initial state upon a given transition, and the attachment density indicates that this portion of density relocates in the target excited state. The ϕ_S_ descriptor corresponds to the overlap between the detachment and attachment densities, ranging from 0 to 1, transcribing the charge transfer character of the reorganization of electron density (i.e, the lower the value of ϕ_S_, the greater the charger transfer).^56^ The *q*^CT^ descriptor quantifies the net charge being effectively transferred upon electronic structure reorganization.^57^ The transition from S0 to S1 upon absorption has a π → π* character and shows little charge transfer (Figure 3b). Comparing the electron densities in the S0 and T5 states (Figure 3c) indicates that reaching T5 via S1 (or any other singlet sate) would result in a significant effective charge transfer and an overall *n* → π* transition involving the lone pair on the phenolate moiety of the molecules as the departing density (detachment). It is noteworthy that this departing density is in close proximity to the heavy iodine atom in DCM_O_-I. The actual transition from S1 to T5 was depicted in Figure 3d, where the electronic structure was calculated in the geometry of S1. The departing density in the transition involved mainly the lone pair of the phenolate moiety and marginally other parts of the molecules. The rather large overlap between detachment and attachment densities of the S1 to T5 transition (ϕ_S_ = 0.62) indicates that the significant, effective charge relocation between S0 and T5 (ϕ_S_ = 0.32) is sequential, with parts resulting from the S0 to S1 transition and the rest occurring upon intersystem crossing from S1 to T5. The large and rather prohibitive energy gap between S1 and T5 might be reduced by accounting for the dynamics of the molecule. In particular, it is likely that the potential energy surface of the S1 and T5 states have significantly different shapes along a specific normal mode or combination of normal modes, allowing them to cross for particular geometries. Following this line of thoughts, we produced an ensemble of 1000 structures from a Wigner distribution at 300 K using the geometry and corresponding normal modes of S1. The first 10 singlet and triplet states were calculated together with their spin orbit coupling, and the data is represented in Figure 4. For each geometry, the energy difference between S1 and each one of the first 10 triplets was plotted against their corresponding spin orbit coupling. The values of the S1–T5 couple in the geometries of S0 and S1 were also represented in the figure. Accounting for the dynamics draws a slightly different picture, in which the energy gap between S1 and triplets to which it is significantly coupled spans a wide range of values, roughly from 0.50 to 1.50 eV. An extreme situation was identified in the distribution, showing a spin orbit coupling of 165 cm^–1^ with an energy gap reduced to 0.38 eV. As represented in the upper panel of the figure, this transition involves S1 and T3 and has a topology (i.e., detachment/attachment densities, ϕ_S_, and *q*^CT^) analogous to that of the S1 to T5 transition discussed above (Figure 3d). The original T5 moved down in the energy spectrum upon vibrational distortion of the molecule without changing the nature of the transition. Our efforts to identify a single normal mode responsible for the decrease in the energy gap were unfortunately unsuccessful. This task would be better addressed by performing nonadiabatic molecular dynamics simulations using software such as SHARC^48−50^ in a manner similar to the work by Zobel et al. for 2-nitronaphthalene^54^ and others.^58−60^ Such a highly elaborate and computationally intensive study is, however, out of the scope of the present paper and may be the subject of a future communication. Nevertheless, the identification of structures in which the energy gap was significantly reduced while retaining a high spin orbit coupling points toward the fact that the intersystem crossing between S1 and T5 (as labeled in the minimum geometry) is possible, yet with a fairly low probability. This may explain the relatively low singlet oxygen quantum yield observed experimentally.

Next, we investigated the impact of iodine and its position on the intersystem crossing capabilities of the molecules. The S0 geometries of DCM_O_, DCM_O_-Cl, I-DCM_O_, and I-DCM_O_-Cl were optimized followed by TD-DFT calculations at the same level as for DCM_O_-I. The data is available as Supporting Information in the form of spin orbit coupling matrices, with energies of the corresponding singlet and triplet states (Tables S7–S11). We found that the electronic structure and the nature of the electronic transition (see natural transition orbitals in Figures S15–S23 in the Supporting Information) are highly similar from one molecule to another. Only the energy of the states slightly varies within a few hundredths of an electron-volt. A significant difference, however, was revealed when analyzing the spin orbit coupling between singlet and triplet states (Tables S7–S11 in the Supporting Information). It appears that beside DCM_O_-I, none of the molecules investigated in this work has a first singlet state with a significant coupling to any triplet. This result was expected for DCM_O_ and DCM_O_-Cl, which are lacking a heavy element. It is more surprising, however, in the case of I-DCM_O_ and I-DCM_O_-Cl. Compared to DCM_O_-I, the latter derivatives present the iodine on the DCM core, and I-DCM_O_-Cl did not show any significant singlet oxygen generation experimentally. The analysis of detachment and attachment densities upon intersystem crossing from S1 to T5 (or T3) in Figures 3d and 4 shows that the electron density on the left-most side of the DCM core was not involved in the reorganization. Instead, the greater part of the departing density (detachment) was located on the phenolate. This, in correlation with experimental observations, tends to indicate that the proximity of the heavy element with the departing electron density is crucial in order to benefit from an enhanced spin orbit coupling and trigger intersystem crossing. We trust that this information will become valuable in the design of future generations of photosensitizers.

**Figure 5:**
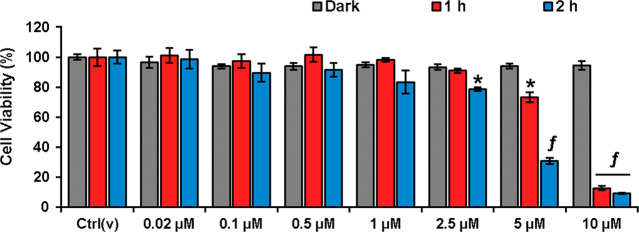
Cell viability of HeLa cells treated with the increasing concentrations (0.02–10 μM) of DCM_O_-I for 24 or 1 or 2 h in the dark, followed by 2 h of LED light (595 nm 9.83 mW/cm^2^) illumination in fresh media, and then dark incubation up to 24 h. Ctrl(v): vehicle control. Data is presented as mean ± SD (*n* = 6). *p < 0.05, *^f^p* < 0.001 vs dark.

Given that DCM_O_-I has the highest ^1^O_2_ quantum yield, we intensified our focus on that core and further tested its potential in cell culture studies. First, photocytotoxicity of DCM_O_-I was investigated by a conventional MTT assay in cancerous HeLa cells. A dose dependent decrease in cell viability was detected after both 1- and 2 h LED light (595 nm, 9.83 mW/cm^2^) irradiation of DCM_O_-I treated cells, with a lower IC_50_ value (3.74 μM) in the case of the 2 h irradiation (Figure 5, Table S3). Almost complete cell death was detected at the 10 μM dose upon 2 h treatment. In contrast, no cell death was detected even at high concentrations when DCM_O_-I incubated cells were kept in the dark, suggesting no dark toxicity, which is one of the critical requirements of a successful PDT agent (Figure 5). Next, HeLa cells were treated with sodium azide (NaN_3_)^61^ and *N*-acetyl cysteine (NAC).^62^ Cell viability increased substantially in the cells that were incubated with NaN_3_, while no remarkable change was observed in NAC treated cells, indicating that ^1^O_2_ is the primary ROS generated during DCM_O_-I induced PDT action (Figure S11).

**Figure 6:**
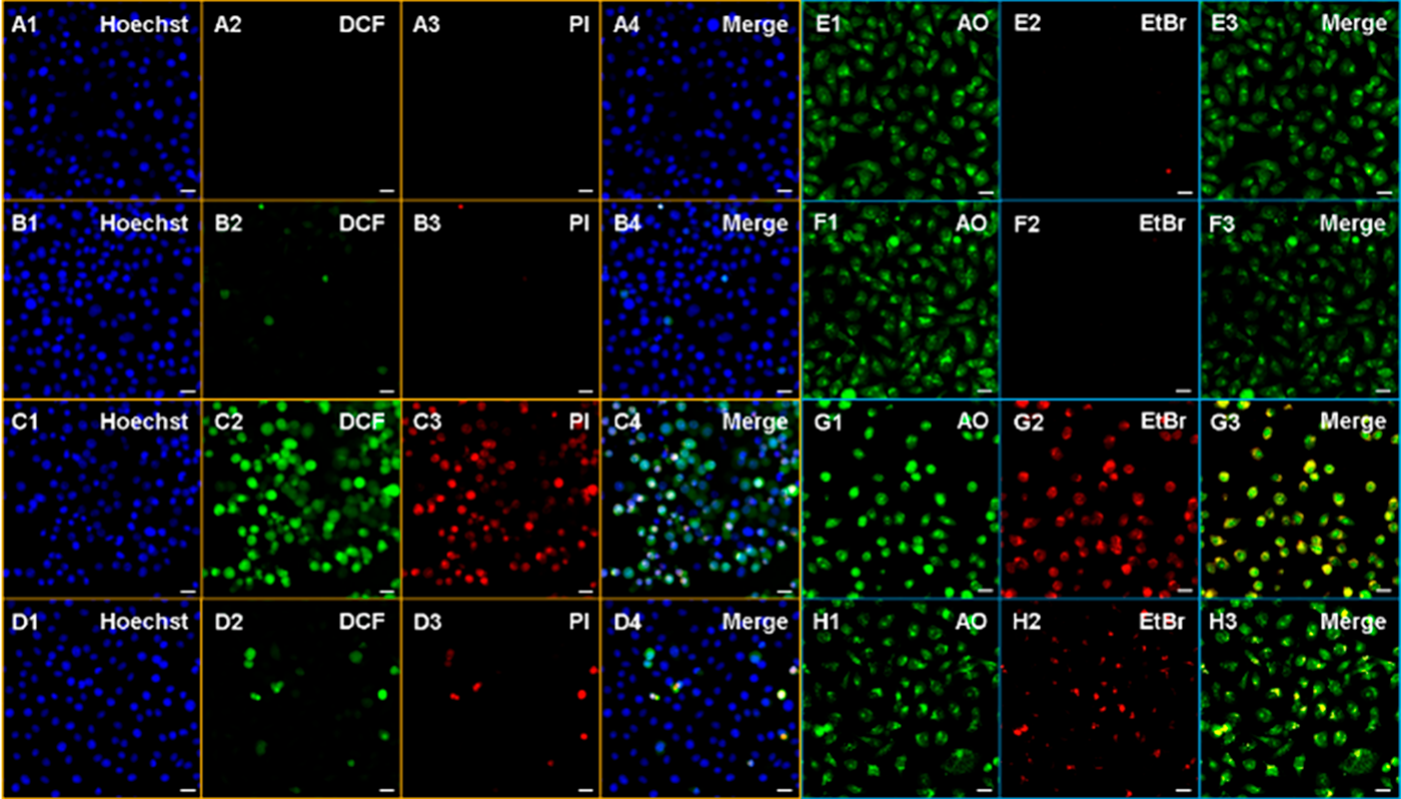
Images of triple Hoechst/DCF-DA/PI (A–D) and dual AO/EtBr (E–H) staining of HeLa cells treated with DMSO (0.2%) (A1–4,E1–3) or IC_50_ values of DCM_O_-I for 2 h in the dark (B1–4,F1–3), followed by 2 h LED light (595 nm 9.83 mW/cm^2^) illumination (C1–4,G1–3) in the presence of NaN_3_ (10 mM) (D1–4,H1–3). Hoechst 33342; DCF, 2′-7′-dichlorofluorescein; PI, propium iodide; AO, Acridine orange; EtBr, ethidium bromide. Scale bar: 50 μm.

Intracellular ^1^O_2_ formation and consequent cell death were further demonstrated under confocal microscopy by using a cell permeable ROS sensor 2′,7′-dichloro-fluorescein diacetate (DCFH_2_-DA), which emits characteristic green light upon oxidation to DCF by ROS, and propidium iodide (PI), which allows detection of cell death with its red fluorescence. Both green and red signals were only detected in DCM_O_-I treated HeLa cells after 2 h of PDT (Figure 6). DCFH_2_-DA is a general ROS sensor, and it is not selective toward singlet oxygen, thus an inhibition experiment was performed to prove that ^1^O_2_ is the major ROS generated during the PDT action. In the case of cells that were treated with NaN_3_, both DCF and PI signals were dramatically quenched even under light irradiation (Figure 6). Additionally, no signal was detected in other control cells, which were either kept in the dark or missing the PS (Figure 6). These results suggest that upon light irradiation, DCM_O_-I primarily triggers ^1^O_2_ generation, which results in oxidative damage induced cell death. To further understand the cell death mechanism (AO)/ethidium bromide (EtBr) costaining was performed. In DCM_O_-I incubated cells, a yellowish color was detected in the merge channel suggesting that cells underwent mostly early apoptosis during the PDT action (Figure 6). In the control groups, only green emission was observed indicating that cells were alive. We also wanted to investigate the intracellular imaging potential of DCM_O_-I. To this end, DCM_O_-I (1 μM) was incubated with HeLa cells and washed, and then images were taken with confocal microscopy. A clear, time dependent and strong cytosolic signal was observed, proving that the DCM_O_-I core can function as a theranostic core as it offers photocytotoxicity and live cell imaging capability at the same time (Figures S12 and S13).

**Figure 7:**
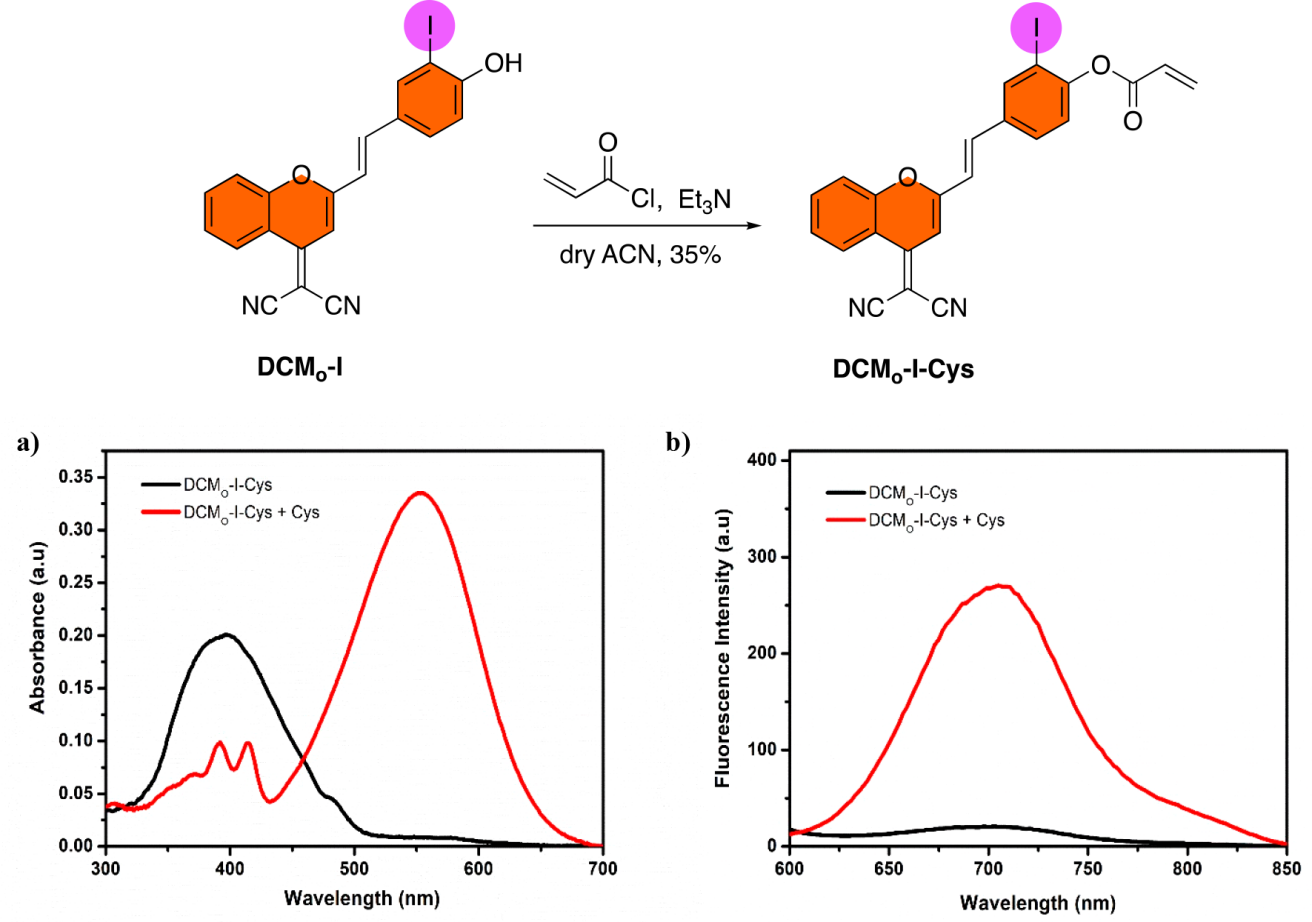
(Top row) Synthesis of DCM_O_-I-Cys. (Bottom row) (a) Absorption and (b) fluorescence emission spectra of DCM_O_-I-Cys (10 μM) with addition of Cys (100 μM) in PBS buffer (50% DMSO, pH 7.4) at 37 °C.

After showing that DCM_O_-I is a highly promising theranostic core, we wanted to convert it to a cancer cell selective activity-based PS. Thus, it can be activated only in cancer cells and potential side effects on healthy cells can be minimized. In this direction, we caged the phenolic group, as in the case of DCM-based imaging probes, by utilizing a cysteine (Cys) responsive acrylate moiety as a masking unit and designed the first example of a Cys activatable DCM-based PS (DCM_O_-I-Cys) (Figure 7). Cys serves as an intracellular antioxidant and is known to be overexpressed in most cancer cells (up to 200 μM) to reduce the high oxidative stress.^63−65^ Therefore, it is accepted as a tumor marker and has been used extensively in activity-based fluorescent sensor designs.^66,67^ However, Cys-responsive therapeutic agents have remained elusive. Recently, we published a Cys activatable hemicyanine-based PS, which marks the first example of a Cys responsive phototherapy agent.^68^ In the caged form, both ^1^O_2_ generation and fluorescence of DCM_O_-I-Cys is blocked due to the restrained intramolecular charge transfer (ICT) process. Michael addition of Cys to acrylate and following cyclization cleave the cage unit and release the active phototheranostic agent DCM_O_-I (Figure S9). Synthesis of DCM_O_-I-Cys was completed in one step by reacting DCM_O_-I with an acryloyl chloride.

**Figure 8:**
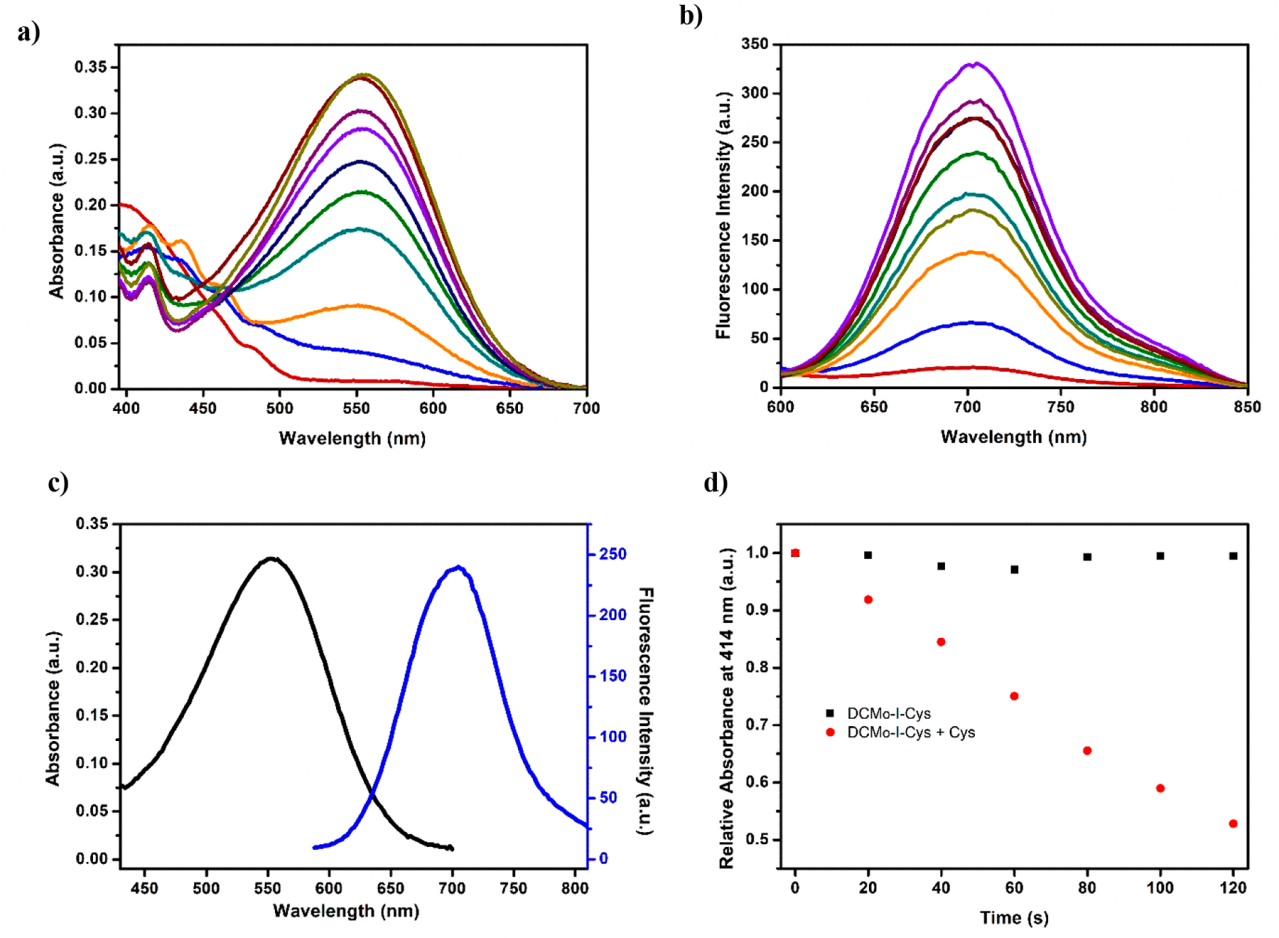
(a) Absorbance and (b) fluorescence spectra of DCM_O_-I-Cys (10 μM) upon addition of different concentrations of Cys (from 0.5 to 140 μM) in PBS buffer (50% DMSO, pH 7.4) at 37 ° C. (c) Stokes shift of DCM_O_-I-Cys in the presence of Cys (100 μM). (d) Relative ^1^O_2_ generation efficiency of DCM_O_-I-Cys (10 μM) prior to and after addition of Cys (100 μM) by using DPBF as a ^1^O_2_ trap molecule. In the case of fluorescence measurements, the excitation wavelength and slit widths were adjusted to 555 nm and 10/10, respectively.

^1^O_2_ generation capacity of DCM_O_-I-Cys was similarly evaluated by using DPBF as a trap molecule in DMSO (1% PBS, pH 7.4). When DCM_O_-I-Cys was irradiated with a 630 nm LED (24.3 mW/cm^2^), no sign of ^1^O_2_ generation was observed as evidenced from the stable absorption signal of the DPBF (Figure 8d, Figure S6). Upon addition of Cys (100 μM), a gradual decrease in the trap absorption was detected (Figure 8d, Figure S7), indicating the formation of an active DCM_O_-I core in the presence of Cys and consequent photosensitized ^1^O_2_ formation.

DCM_O_-I-Cys exhibited a blue-shifted absorption signal in PBS (50% DMSO, pH 7.4) which was centered at 395 nm. Upon treating DCM_O_-I-Cys with increasing concentrations (0–100 μM) of Cys, a strong absorption peak at 555 nm appeared rapidly (∼1 min), which belongs to the active core DCM_O_-I (Figures 7a and 8a). In the case of fluorescence measurements, similarly a very fast turn-on response was detected at 705 nm upon addition of Cys (λ_ex_ = 555 nm), which possessed up to a 14-fold enhancement in the emission intensity with a large Stokes shift (150 nm) when 100 μM Cys was added (Figures 7b and 8b,c). Reaction of Cys with DCM_O_-I-Cys was also monitored by HPLC (Figure S8). Treating DCM_O_-I-Cys with a Cys (100 μM) resulted in a signal that was eluted at the same minute with the parent DCM_O_-I core, indicating DCM_O_-I is released upon activation process (Figure S9, Table S2). The response of DCM_O_-I-Cys to glutathione (GSH), another abundant intracellular biothiol in cancerous cells, was also checked in aqueous solutions. Treating DCM_O_-I-Cys with GSH (100 μM) resulted in a weak absorption signal at 555 nm along with a poor fluorescence intensity at 705 nm compared to Cys treatment (Figure S10), suggesting that Cys is the predominant biothiol that triggers the activation process.

**Figure 9:**
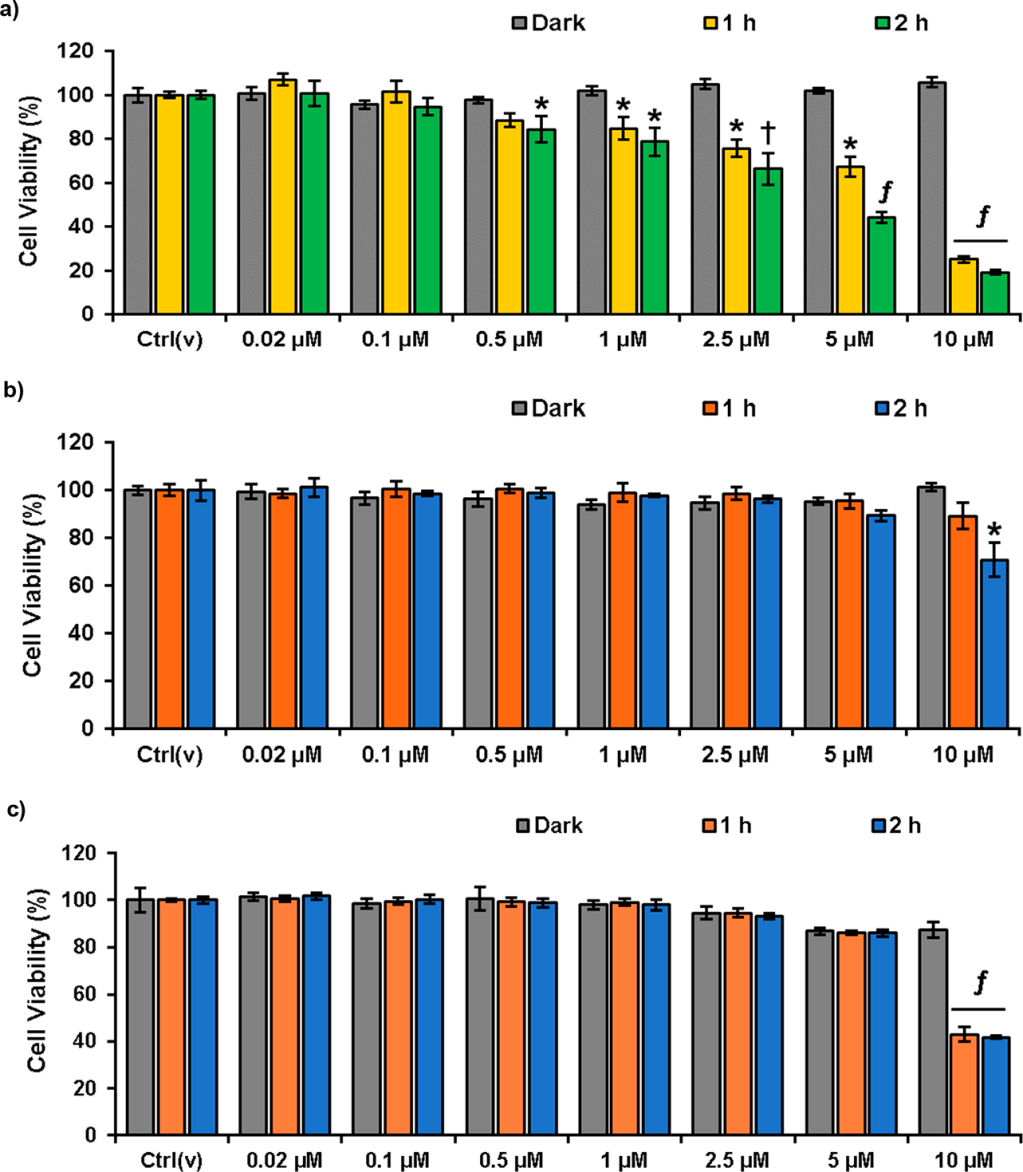
Cell viabilities of (a) HeLa and (b) L929 cells treated with the increasing concentrations (0.02–10 μM) of DCM_O_-I-Cys for 1 or 2 h in the dark, followed by 2 h of LED light (595 nm 9.83 mW/cm^2^) illumination in fresh media and then dark incubation up to 24 h. (c) Cell viability of L929 cells treated with the increasing concentrations (0.02–10 μM) of DCM_O_-I for 1 or 2 h in the dark, followed by 2 h of LED light (595 nm 9.83 mW/cm^2^) illumination in fresh media and then dark incubation up to 24 h. In all cases, a group of cells was kept in the dark for 24 h to test the dark toxicity. Ctrl(v): vehicle control. Data is presented as mean ± SD (*n* = 6). *p < 0.05, ^†^*p* < 0.01, ^*f*^*p* < 0.001 vs dark.

Next, we tested the cytotoxicity of DCM_O_-I-Cys in cancerous HeLa cells with a high Cys level^69,70^ and normal L929 cells to further investigate its selectivity. DCM_O_-I-Cys did not show any dark toxicity in both cells in the whole concentration range (0–10 μM) (Figure 9a,b). A dose dependent cell death was determined in DCM_O_-I-Cys treated HeLa cells after 1 h and 2 h light (595 nm, 9.83 mW/cm^2^) irradiation. Cell viability dropped to 15% at a 10 μM dose, and the IC_50_ was found to be 4.33 μM for 2 h excitation (Figure 9a, Table S3). In the case of 1 h irradiation, IC_50_ was appeared to be slightly higher and calculated as 6.11 μM (Figure 9a, Table S3). On the other side, when normal L929 cells were subjected to same experiment under identical conditions, significantly lower cell death was observed, and cell viability stayed around 80% even at the highest dose (10 μM) (IC_50_ > 10 μM) (Figure 9b, Table S3). In contrast, when L929 cells were treated with “always on” DCM_O_-I (10 μM), the cell viability dropped to 40% as it cannot differentiate between normal and cancer cells (Figure 9c). Treating HeLa cells with NaN_3_ increased the cell viability in a concentration dependent manner, while NAC inhibition did not show any effect as in the case of DCM_O_-I core (Figure S11).

**Figure 10:**
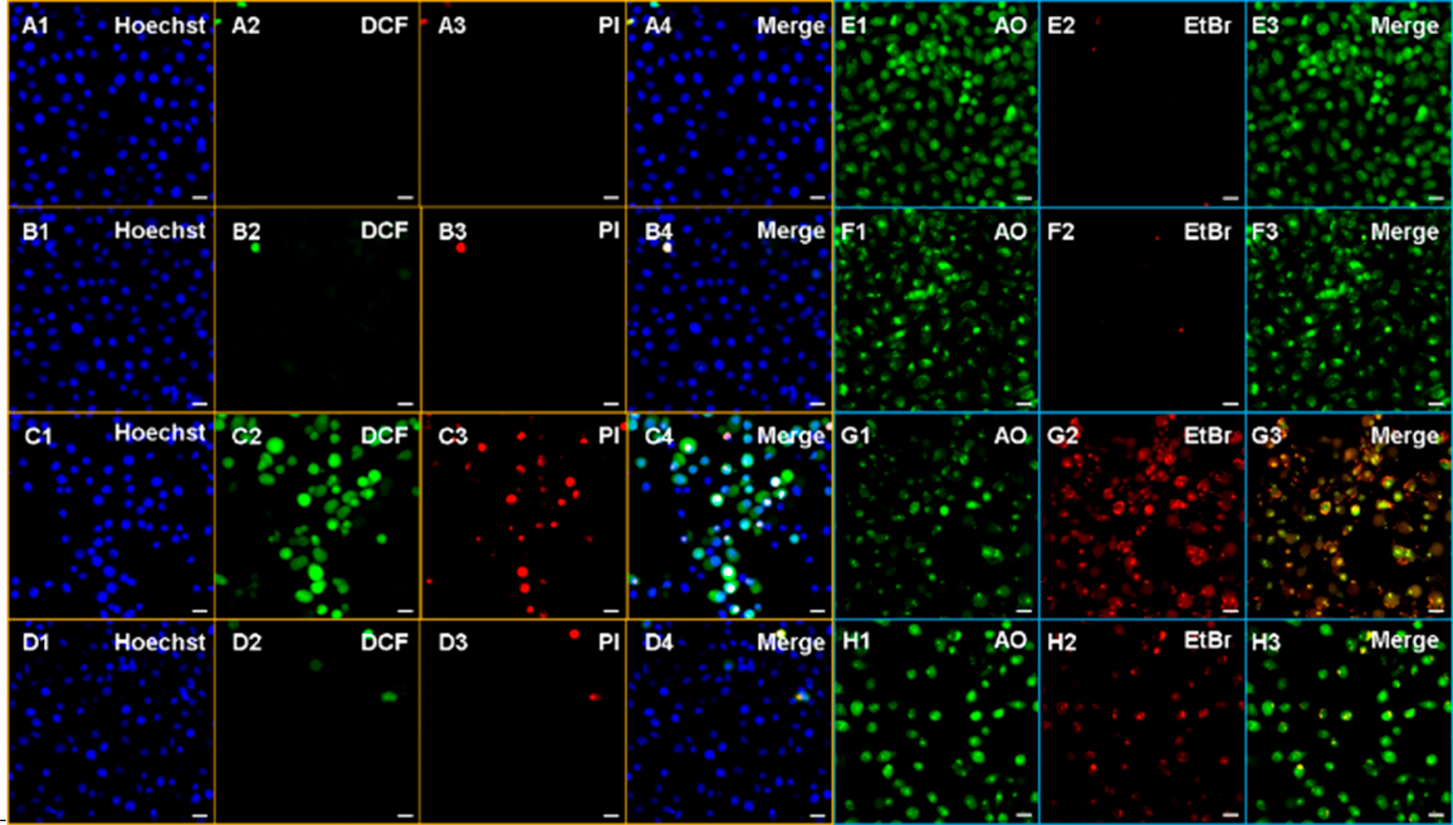
Images of triple Hoechst/DCF-DA/PI (A–D) and dual AO/EtBr (E–H) staining of HeLa cells treated with DMSO (0.2%) (A1–4,E1–3) or IC_50_ values of DCM_O_-I-Cys for 2 h in the dark (B1–4,F1–3), followed by 2 h of LED light (595 nm, 9.83 mW/cm^2^) illumination (C1–4,G1–3) in the presence of NaN_3_ (10 mM) (D1–4,H1–3). Hoechst 33342; DCF, 2′-7′-dichlorofluorescein; PI, propium iodide; AO, acridine orange; EtBr, ethidium bromide. Scale bar: 50 μm.

DCM_O_-I-Cys induced ROS generation was also monitored in HeLa cells by using a DCFH_2_-DA sensor and green emission was only detected when the DCM_O_-I-Cys incubated cells were irradiated (Figure 10). Similarly, cell death was only detected in the same group of HeLa cells. PI staining along with AO/EtBr imaging showed that mostly late apoptotic/necrotic cells exist after PDT action (Figure 10). Similar to the DCM_O_-I core, NaN_3_ inhibition substantially reduced ^1^O_2_ generation and the alive cell ratio appeared to be higher. Control cells, which were either not treated with DCM_O_-I-Cys or kept in the dark exhibited no ROS generation and cytotoxicity as expected (Figure 10). Cumulative results suggest that DCM_O_-I-Cys is activated by endogenous Cys and triggers ^1^O_2_-induced selective photocytotoxicity toward cancer cells possessing high Cys activity.

**Figure 11:**
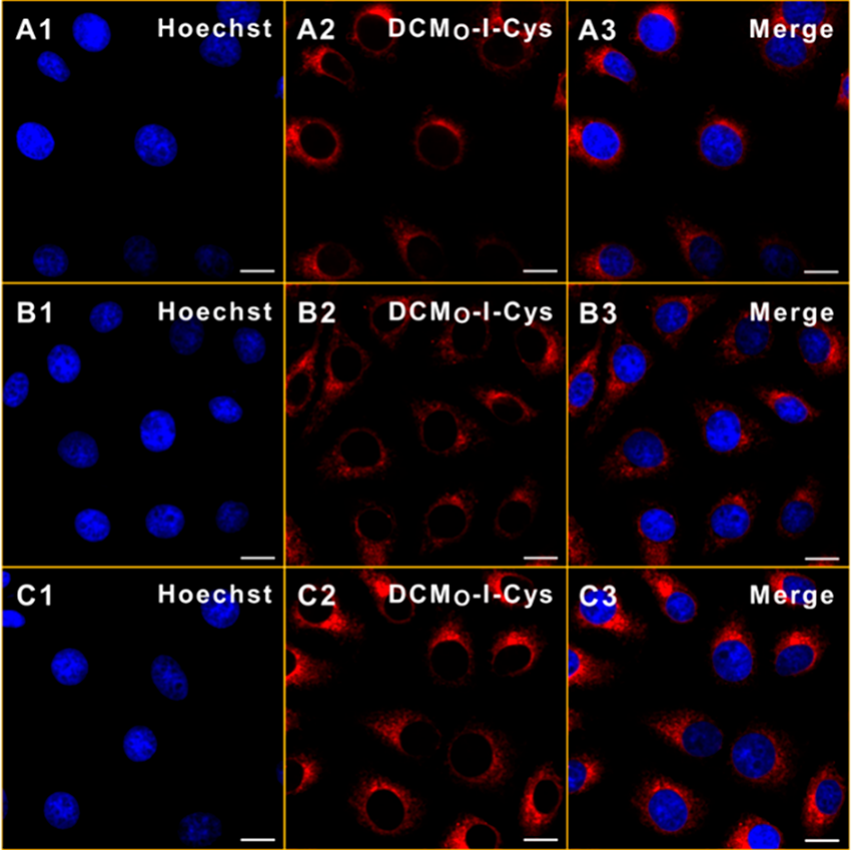
Time dependent cellular internalization of DCM_O_-I-Cys in HeLa cells after 30 min (A1–3), 1 h (B1–3), and 2 h (C1–3). Blue, Hoechst 33342; red, DCM_O_-I-Cys. Scale bar: 10 μm.

Finally, the imaging potential of DCM_O_-I-Cys was evaluated under confocal microscopy. In HeLa cells, a time dependent increase in the cytosolic fluorescence signal was observed indicating that the agent can be utilized as an activatable theranostic agent (Figure 11). On the other side, DCM_O_-I-Cys was also getting activated to some extent in L929 cells; however, the intensity of the signal is lower compared to HeLa cells (Figure S13), and the agent cannot be sufficiently activated to induce effective cell death as it is observed in cell viability results.

## 4 Conclusion

In summary, oxygen substituted DCM was designed as a phototheranostic PDT agent for the first time by introducing an iodine atom on to the core structure. It was found that the presence of iodine atom by itself is not sufficient to get a high ^1^O_2_ generation yield as understood from chemical trap experiments, but its location is also highly critical. DCM_O_-I, which bears the iodine on the phenolate ring performed better compared to other PS I-DCM_O_-Cl, which holds iodine on the chromene ring. TDDFT calculations suggest a rationale for the enhanced singlet oxygen production activity of DCMo-I. Analysis of the electronic topology of the target triplet state involved in intersystem crossing shows that the departing density, i.e., the electron density that is moved from the initial to the final state, is highly localized on the phenolate moiety of the molecule. The proximity of the iodine to the departing electron density appears to have a significant impact on the magnitude of the spin orbit coupling, explaining the lower activity in I-DCM_O_-Cl. When DCM_O_-I was tested in cell cultures, it exhibited high photocytotoxicity in HeLa cancer cells along with a strong fluorescence signal, proving its phototheranostic nature. Based on the promising results obtained with DCM_O_-I, the core was utilized to develop a Cys activatable aPSs (DCM_O_-I-Cys) by masking the phenol with a Cys responsive cage unit. Upon treating with Cys in aqueous solutions, a dramatic red shift in the absorption signal with a concomitant turn-on response in fluorescence was observed. DCM_O_-I-Cys induced significant photocytotoxicity in HeLa upon getting activated by an endogenous Cys. In contrast, it stayed in its off state in L929 cells, and cell viabilities remained unperturbed. These observations were supported by DCFH_2_-DA and PI/EtBr/AO staining studies. DCM_O_-I-Cys was also used successfully to image the Cys activity in cancer cells under confocal microscopy. DCM_O_-I core offers highly attractive advantages compared to first-generation PSs, such as ease of modification toward development of cancer cell selective PDT agents as in the case of DCM_O_-I-Cys and opportunities for theranostic applications. It is worth mentioning that the DCM core shows strong two-photon absorption (TPA) characteristics, which can be implemented to DCM-based PSs to satisfy deep tissue penetration in *in vivo* studies. Our effort in this direction is in progress. To close, DCM_O_-I-Cys marks the first ever example of a Cys-responsive DCM-based PS. DCM-based PSs introduce exiting new gateways for cancer cell specific therapeutics, particularly in the scope of phototherapies.

## Supporting Information Available

Materials and instruments, synthetic details, ^1^H, ^13^C NMR, and HR-MS spectra, details of photophysical characterization, additional photophysical measurements, HPLC chromatograms, ^1^O_2_ trap experiments, details of cell culture and imaging experiments, additional cell culture studies, details of computational studies, and additional figures and tables related to computational studies (PDF)

## Abbreviations

PDT: photodynamic therapy
PS: photosensitizer
ROS: reactive oxygen species
DCM: dicyanomethylene- 4*H*-chromene
ALP: alkaline phosphatase
ICT: intramolecular charge transfer process
Cys: cysteine
DPBF: 1,4-diphenylbenzofuran
TD-DFT: time-dependent density functional theory
PBS: phosphate buffer saline
ISC: intersystem crossing
DMSO: dimethyl sulfoxide
MTT: 3-(4,5-dimethylthiazolyl- 2)-2,5-diphenyltetrazolium bromide
PI: propidium iodide
DCFH_2_-DA: 2′,7′-dichloro- fluorescein diacetate
NAC: *N*-acetyl cysteine
NaN_3_: sodium azide
LED: light emitting diode
AO: acridine orange
EtBr: ethidium bromide
TPA: two-photon absorption
aPS: activatable photosensitizer
IC_50_: half maximal inhibitory concentration
