# Supplementary material for: Activity-Based Photosensitizers with Optimized Triplet State Characteristics Toward Cancer Cell Selective and Image Guided Photodynamic Therapy
Source: ACS Appl Bio Mater. 2022 May 10;5(6):2754–67. doi: 10.1021/acsabm.2c00202 (PMC9214761; doi:10.1021/acsabm.2c00202)
Supplement: Supplementary file 1 — mt2c00202_si_001.pdf [file mt2c00202_si_001.pdf]

Supporting Information

Activity-Based Photosensitizers with Optimized Triplet State Characteristics Toward Cancer Cell Selective and Image Guided Photodynamic Therapy

Eda Kilic,[a§] Zubeyir Elmazoglu,[b§] Toghrul Almammadov,[a] Dilay Kepil,[b] Thibaud Etienne,[c] Antoine Marion,[b] Gorkem Gunbas,[b]* Safacan Kolemen[a,d,e,f]*

[a] Department of Chemistry, Koç University, 34450 Istanbul, Turkey

[b] Department of Chemistry, Middle East Technical University (METU), 06800, Ankara, Turkey

[c] Université de Lorraine, CNRS, LPCT, F-54000 Nancy, France

[d] Surface Science and Technology Center (KUYTAM), Koç University, 34450 Istanbul, Turkey

[e] Boron and Advanced Materials Application and Research Center, Koç University, 34450 Istanbul, Turkey

[f] TUPRAS Energy Center (KUTEM), Koç University, 34450 Istanbul, Turkey

[§] *E.K.and Z.E. contributed equally to this paper.*

*Email: ggunbas@metu.edu.tr ; skolemen@ku.edu.tr

Materials and Methods

All reagents were commercially available and used without further purification unless otherwise noted. All dry solvents used in reactions were directly obtained from the Mbraun MBSPS5 solvent drying system. The inert atmosphere was obtained by Argon. The ^{1}H and ^{13}C-NMR spectra were recorded on a Varian (500 MHz) or Bruker Avance III Ultrashield (400 MHz) spectrometers using $CDCl_3$ or d_6-DMSO as the solvents. The chemical shifts are reported in parts per million (ppm) downfield from an internal TMS (trimethylsilane) reference. Coupling constants (*J*) are reported in hertz (Hz), and the spin multiplicities were specified by the following symbols: s (singlet), d (doublet), t (triplet), and m (multiplet). NMR spectra were processed with MestReNova program. Column chromatography was performed by using thick-walled glass columns and silica Gel 60 (Merck 230-400 mesh). Thin layer chromatography (TLC Merck Silica Gel 60 F254) was performed by using commercially prepared 0.25 mm silica gel plates and visualization was provided by UV lamp. The relative proportions of solvents in chromatography solvent mixtures refer to the volume: volume ratio. Electronic absorption spectra in solution were acquired using a Shimadzu Uv-3600 Uv-Vis-NIR spectrophotometer. Mass spectra were recorded on Waters Synapt G1 High-Definition mass spectrometer. HPLC analysis was performed on an Agilent 1260 series.

1. Photophysical Characterization

Unless otherwise stated, all the absorbance and fluorescence measurements were made according to the following procedure. In a 3-mL quartz cell of 1-cm optical length, 10 µM of the stock solution of DCM, DCM_O-I and I-DCM_O-Cl was dissolved in DMSO–PBS buffer (10 mM, pH 7.4, 1:1, v/v) at 37 °C. In all fluorescence measurements, both excitation and emission slit widths were set to 10 nm.

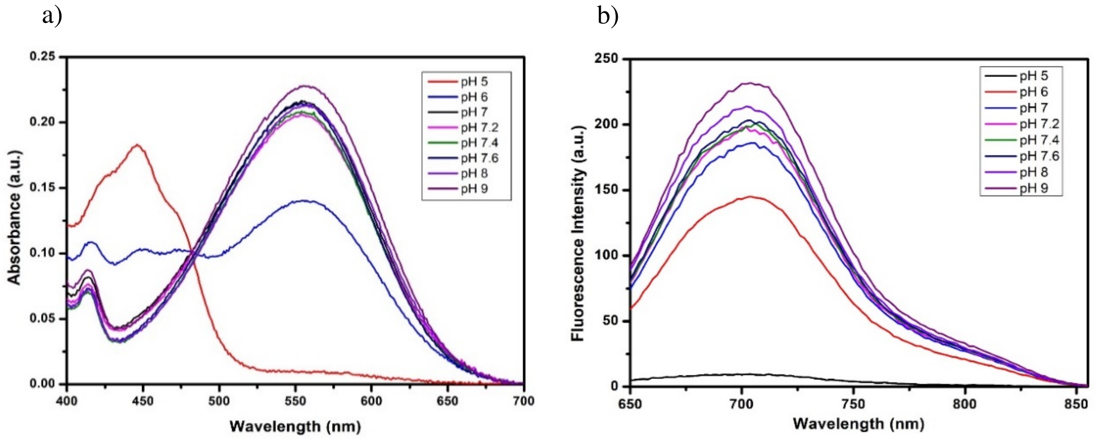

Figure S1 (a) Absorption and (b) fluorescence emission spectra of DCMo-I in aqueous solutions with different pH values.

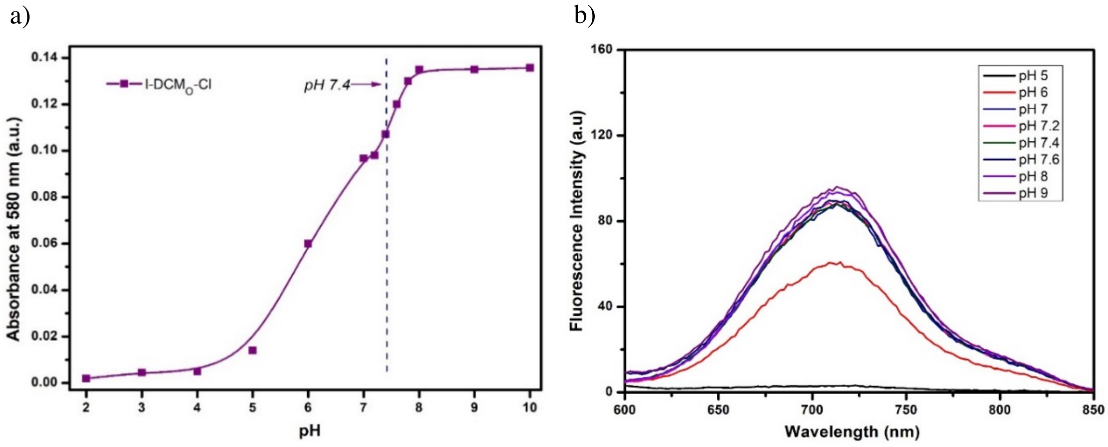

Figure S2 (a) Absorption and (b) fluorescence emission spectra of I-DCM_O-Cl in aqueous solutions with different pH values.

Table S1 Photophysical parameters and $^1O_2$ quantum yields of DCM derivatives.

	λ_{abs} (nm)	λ_{em} (nm)	$\varepsilon(M^{-1}cm^{-1})$	ϕ_F (%)[a]	ϕ_Δ (%)
DCM	560	705	20600	58[1]	n.d.
DCM$_O$-I	555	728	22750	24	5.2
I-DCM$_O$-Cl	560	738	11500	6	0.6

(a) The fluorescence quantum yield measurement was carried out using B-DCM-N in DCM (quantum yield was reported as 0.5958 in DCM)[2]. All experiments were done in DMSO (containing 1% PBS). For fluorescence measurement all spectra were collected with a slit width: (10/10).

Chemical Detection of Singlet Oxygen

Singlet oxygen generation of DCM, DCM$_O$-I and I-DCM$_O$-Cl was determined by using 1,4-diphenylbenzofuran (DPBF) in DMSO–PBS buffer (10 mM, pH 7.4, 1:1, v/v). Methylene blue (Φ_Δ = 0.49 in DMSO[3]) was used as a reference compound for singlet oxygen quantum yield calculations. DCM, DCM$_O$-I or I-DCM$_O$-Cl were mixed in oxygen bubbled PBS and DMSO. The solutions were exposed to LED light source of 630 nm from 15 cm of distance and 20 second time interval for all the samples. The absorbance of DPBF was determined after each irradiation to evaluate producing $^1O_2$. Singlet oxygen quantum yields were calculated according to the equation given below:

$$\phi_{\Delta sample} = \phi_{\Delta standard}\left(\frac{1-10^{-A_{std}}}{1-10^{-A_{sam}}}\right)\left(\frac{m_{sample}}{m_{standard}}\right)$$

where sample and standard represent DCM, DCM$_O$-I or I-DCM$_O$-Cl and methylene blue respectively. m is the slope of absorbance maxima of DPBF at 414 nm versus time graph. A is the absorbance value of both sample and standard at irradiation wavelength, 630 nm.

a) b)

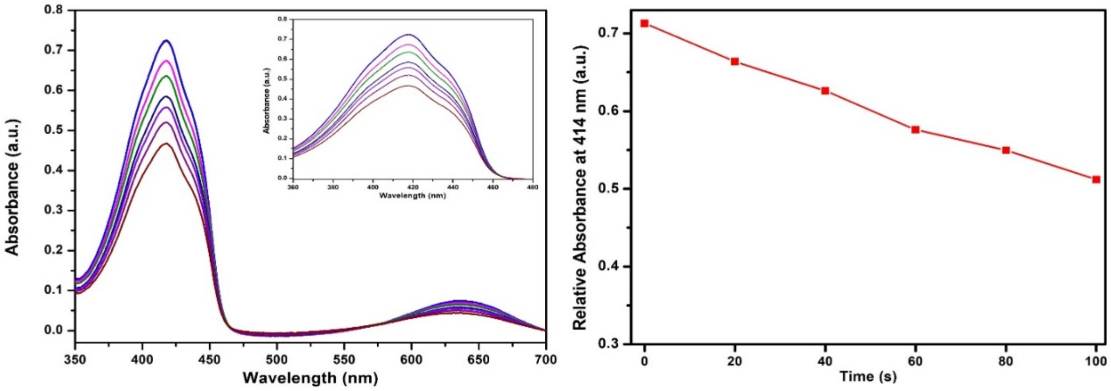

Figure S3 (a) Absorption spectra of DPBF upon irradiating DCM$_O$-I (10 μM) containing DMSO solution (1% PBS, pH 7.4) with a 630 nm LED light. (b) change in the absorption signal of DPBF at 414 nm upon irradiation with 630 nm LED light.

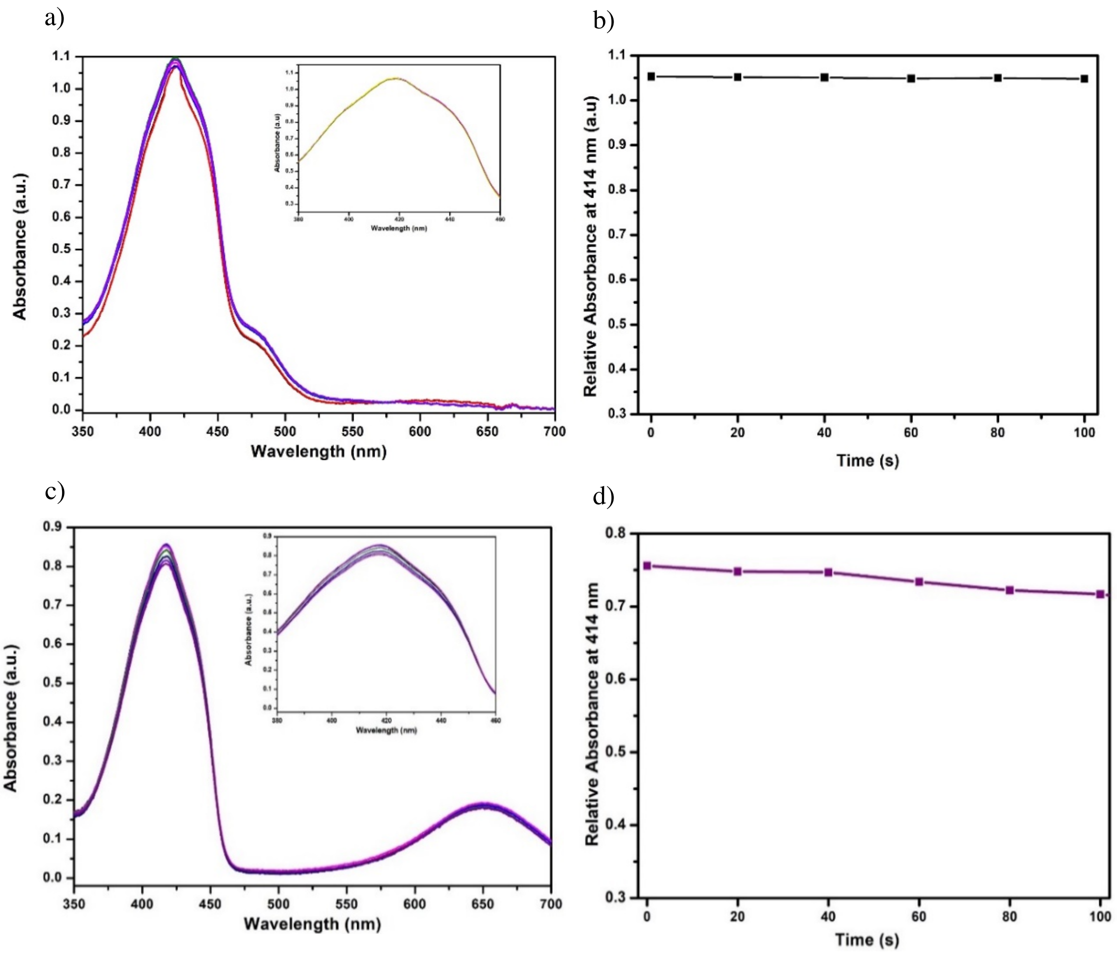

Figure S4 Change in the absorption spectra of (a) DCM and (c) I-DCM$_O$-Cl in 1% PBS/DMSO (1:99) solution containing DPBF trap molecule upon irradiation with a 630 nm LED light; (b), (d) change in the absorption signal of DPBF at 414 nm upon irradiation (b) DCM and (d) I-DCM$_O$-Cl with 630 nm.

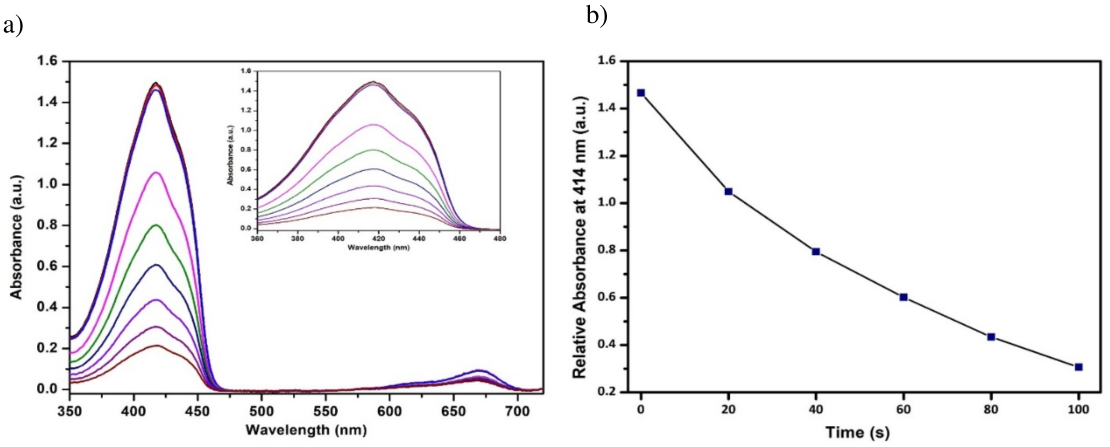

Figure S5 (a) Absorption spectra of DPBF upon irradiating methylene blue containing DMSO solution (1% PBS, pH 7.4) with a 630 nm LED light. (b) change in the absorption signal of DPBF at 414 nm upon irradiation with 630 nm LED light.

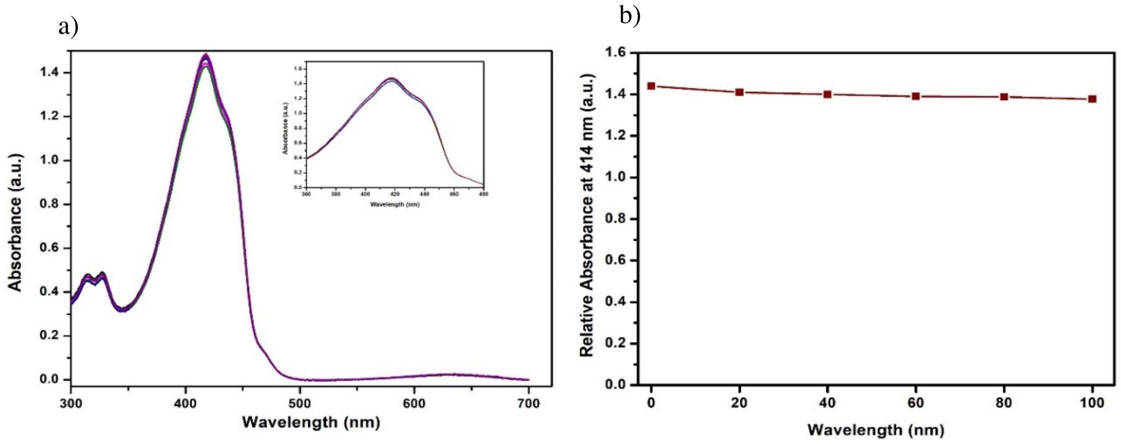

Figure S6 (a) Absorption spectra of DPBF upon irradiating DCM$_O$-I-Cys containing DMSO solution (1% PBS, pH 7.4) with a 630 nm LED light. (b) change in the absorption signal of DPBF at 414 nm upon irradiation with 630 nm LED light.

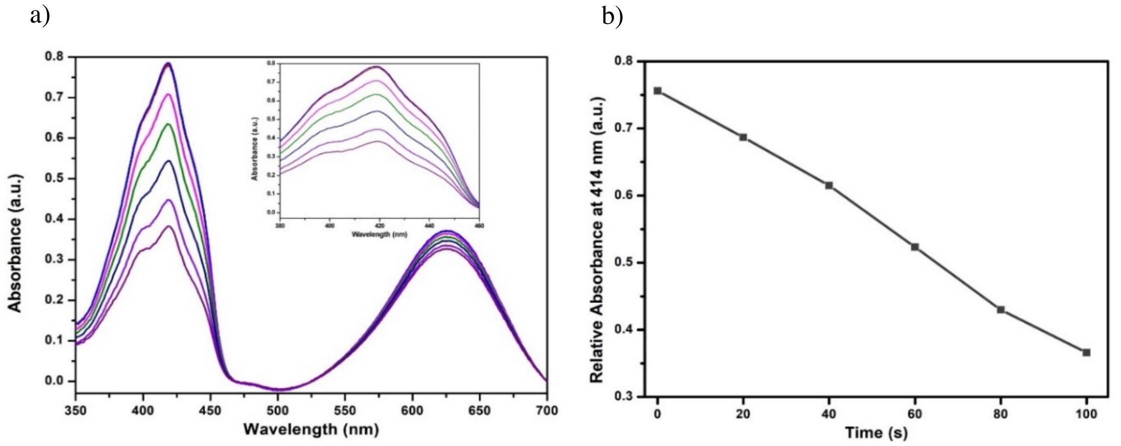

Figure S7 (a) Absorption spectra of DPBF upon irradiating DCM$_O$-I-Cys + Cys containing DMSO solution (1% PBS, pH 7.4) with a 630 nm LED light. (b) change in the absorption signal of DPBF at 414 nm upon irradiation with 630 nm LED light.

Fluorescence Quantum Yield Calculation

The fluorescence quantum yield measurements of **DCM$_O$-I** and **I-DCM$_O$-Cl** were carried out using B-DCM-N (quantum yield was reported as 59.58% in DCM)[5] as the reference. For both measurements, the samples were dissolved in DMSO (10 mM, containing 1% PBS) and absorbance values of samples at their excitation wavelengths were kept less than 0.1.

The fluorescence quantum yield ($\boldsymbol{\phi}_F$) was calculated by using the following formula:

$$\mathbf{QY}_{sample} = \mathbf{QY}_{standard} \frac{\boldsymbol{F}_{sample}\, \boldsymbol{A}_{standard}\, \boldsymbol{n}^2_{sample}}{\boldsymbol{F}_{standard}\, \boldsymbol{A}_{sample}\, \boldsymbol{n}^2_{standard}}$$

where $\mathbf{QY}_{sample}$ and $\mathbf{QY}_{standard}$ are the fluorescence quantum yields of the sample and the standard (B-DCM-N), respectively. F_{sample} and $F_{standard}$ are the integrated fluorescence emission of the sample, A_{sample} and $A_{standard}$ represent the absorbance of the sample and standard at their respective excitation wavelengths. Measurements were carried out in 1 cm quartz cuvettes with a total sample volume of 3 mL.

2. HPLC Analysis:

HPLC analysis was performed using RP-HPLC System with UV-Vis detection and a reversed-phase C18 column (4 μm, 4.6 × 150 mm). The data collect and analysis was carried out using the Chemstation software. The oven temperature was maintained at 25 ˚C, the injection volume was 20 μL and the flow rate was 1.0 mL/min with detection wavelength at 480 nm.The separation program of gradient elution was as follows ; where solvent A was water with 0.1% TFA and solvent B was acetonitrile.

Table S2 RP-HPLC Conditions

Time/min	Phase A/%	Phase B/%
0	10.0	5.0
7.0	5.0	95.0
13.0	5.0	95.0
16.0	95.0	5.0

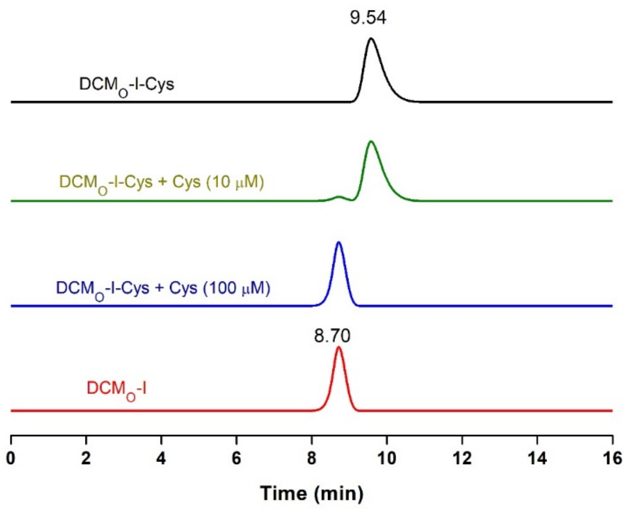

Figure S8 HPLC chromatograms of DCM_O-I-Cys before and after treatment with either 10 μM Cys or 100 μM Cys at 37 °C and DCM_O-I.

3. Activation Mechanism of DCM$_O$-I-Cys in presence of Cysteine

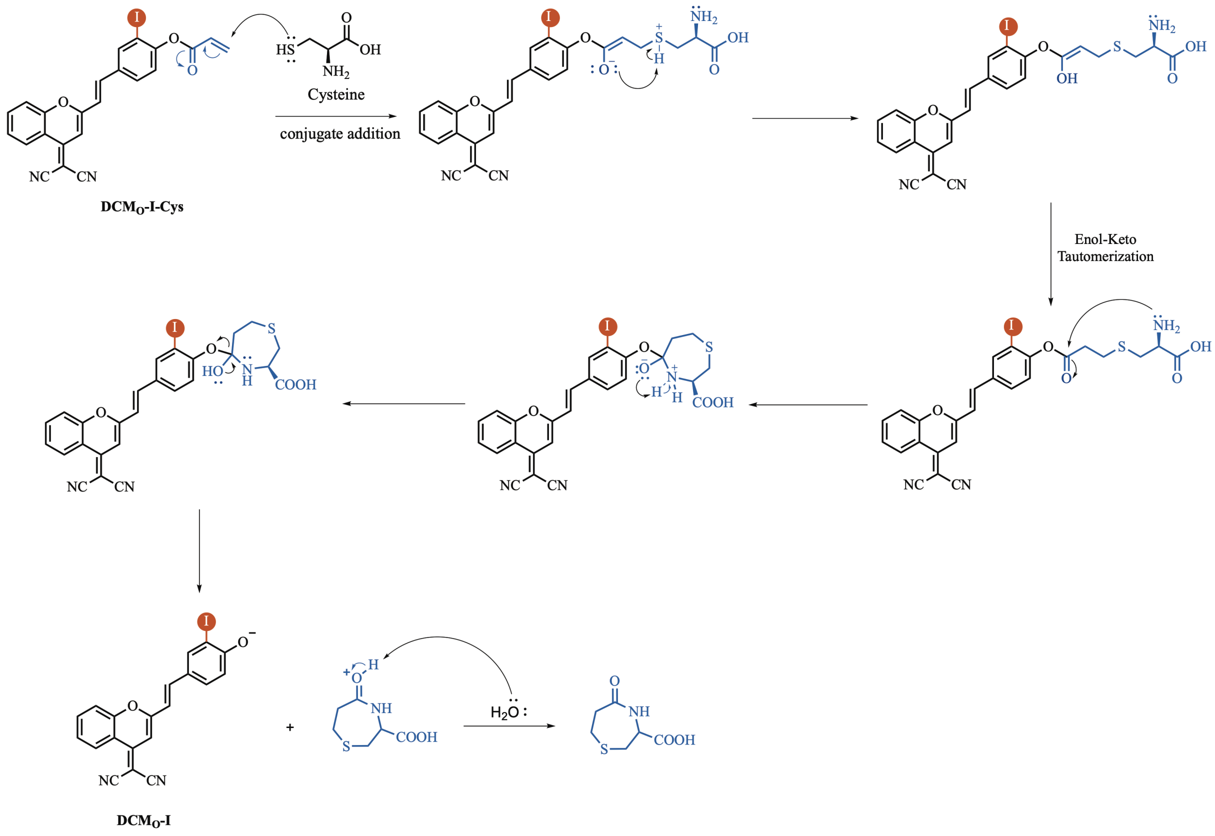

Figure S9 Proposed activation mechanism of DCM$_O$-I-Cys in the presence of Cys.

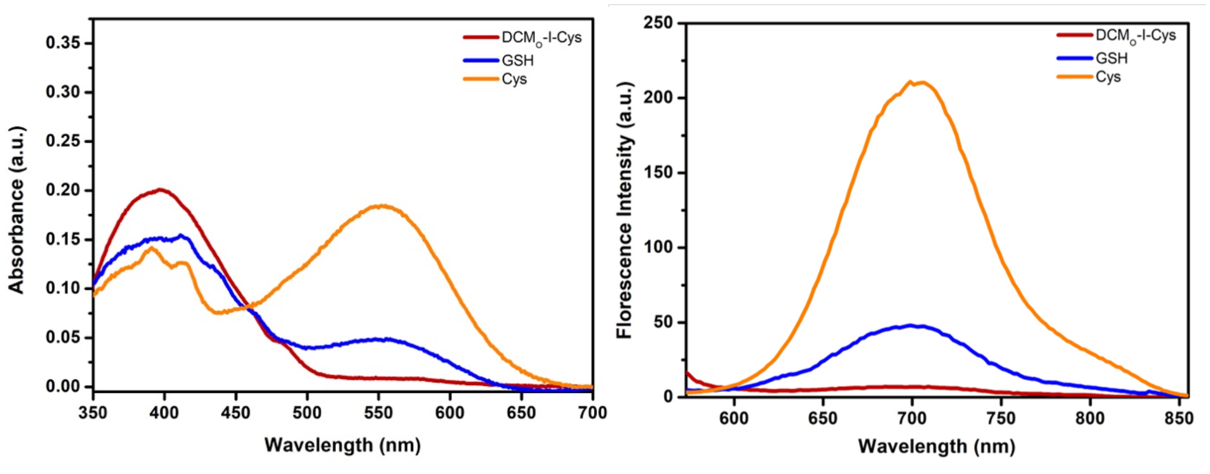

Figure S10 Absorbance (left) and fluorescence (right) spectra of DCM$_O$-I-Cys (10 μM) before and after addition of Cys (100 μM) or GSH (100 μM). Measurements were performed in DMSO–PBS buffer (10 mM, pH 7.4, 1:1, v/v) at 37˚C at 37 °C. Excitation 555 nm with slit width (10, 10).

4. Cell Culture and Treatments

Table S3 IC_{50} values of DCM_O-I and DCM_O-I-Cys in HeLa and L929 cells.

**HeLa**	DCM_O-I.	DCM_O-I-Cys	**L929**	DCM_O-I.	DCM_O-I-Cys
2 h	3.74 μM	4.33 μM	2 h	8.96 μM	10 μM <
1 h	6.32 μM	6.11 μM	1 h	9. 10 μM	10 μM <

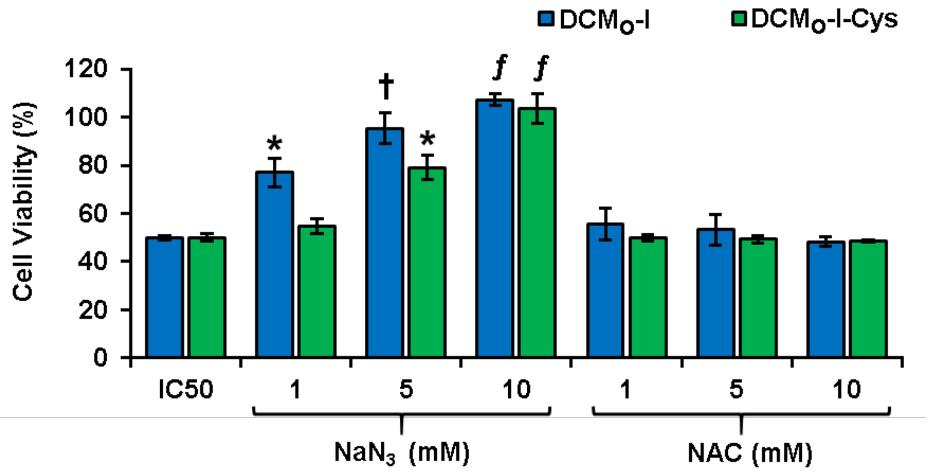

Figure S11 Cell viability of HeLa cells treated with the IC_{50} values of DCM_O-I (3.74 μM) or DCM_O-I-Cys (4.33 μM) for 2 h at dark, followed by 2 h LED light (595 nm 9.83 mW/cm^2) illumination in the presence or absence of NaN_3 (1-10 mM) or NAC (1-10 mM) and then dark incubation up to 24 h. Ctrl(v): vehicle control, NAC: *N*-acetlycsyteine, NaN_3: sodium azide. (n=5-6). *$p<0.05$, $^{\dagger}p<0.01$, $^{f}p<0.001$ *vs* IC_{50}.

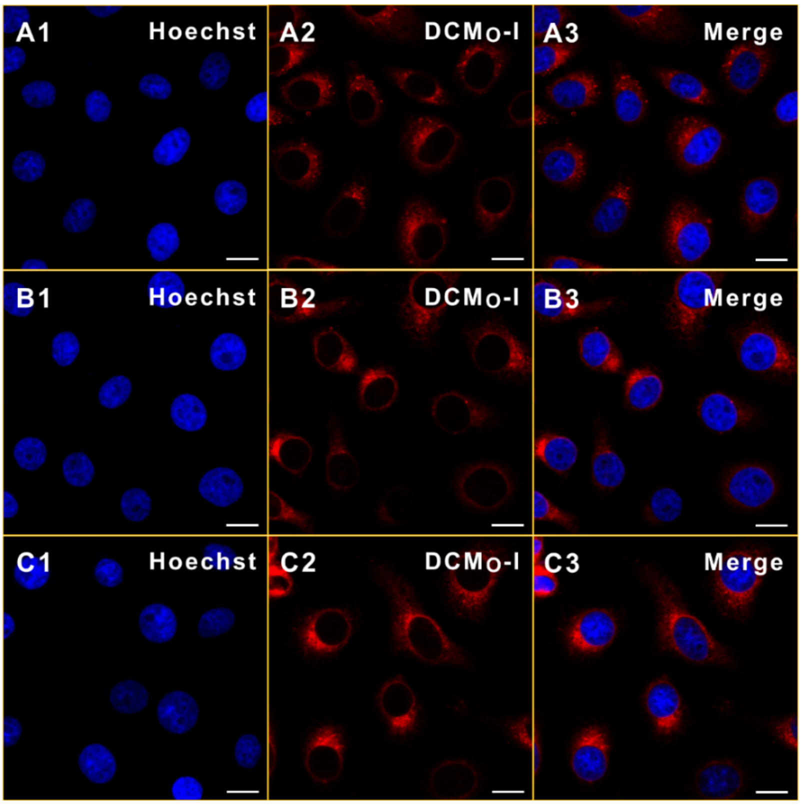

Figure S12 Time dependent cellular internalization of DCM_O- I in HeLa cells after 30 min (A1-3), 1 h (B1-3), 2 h (C1-3). Blue: Hoechst 33342; red: DCM_O- I. Scale bar: 10 μm.

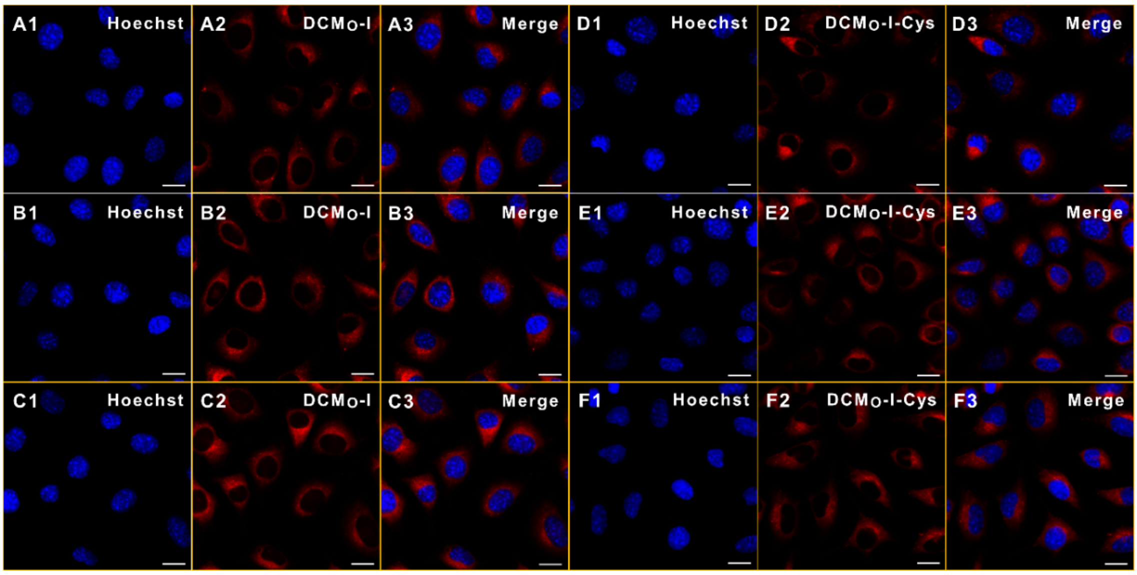

Figure S13 Time dependent cellular internalization of DCM_O- I (A-C) and DCM_O-I-Cys (D-F) in L929 cells after 30 min (A1-3, D1-3), 1 h (B1-3, E1-3), 2 h (C1-3, F1-3). Blue: Hoechst 33342; red: DCM_O- I or DCM_O-I-Cys. Scale bar: 10 μm.

5. Computational Studies:

TDDFT benchmark

A benchmark of density functionals and basis sets was conducted to determine the method of choice for the present study. The data is available Table S4-S5 and a summary is presented in Table S6. In the latter, we report the error made by the respective methods for the energy difference between bright singlet states of DCM_O-I in DMSO compared to the experimental spectrum. We notice that the topology of the excited states does not change from one method to another (data not shown), and that only the energies and ordering of the states vary. Only the range-separated double-hybrid functionals with spin-component or spin-opposite scaling SCS/SOC-ωPBEPP86 reproduces accurately the energy difference between the ground state (S0) and the first excited singlet state (S1), with an error of 0.023 eV. The difference in energy between S1 and S2, as well as that between S2 and the third bright state (i.e., S3 or S4 depending on the method, further referred to as S3/4), are significantly over-estimated by the method with errors of 0.424 and 0.382 eV, respectively. The double-hybrid range separated functional with spin-component scaling B97X-2 significantly underestimates the S0-S1 energy difference and gives an unsatisfactory picture of the excited states. Other range-separated and double-hybrid range-separated functionals tested in this study were also found unsatisfactory. The hybrid functionals B3LYP, PBE0, and TPSSH, however, yield a fair prediction of the energy of S1 relative to the ground state, and offer the best energy difference between excited states over the methods tested in this benchmark. B3LYP slightly over-estimates the energy gap between S0 and S1 by 0.281 eV. The difference between S1-S2 and S2-S3/4 is, however, very well-reproduced by the method with errors compared to experiments of only 0.003 and -0.069 eV, respectively. Additionally, we find that B3LYP reproduces fairly well the solvent-induced red-shift on the absorption spectrum compared to other functional, while the choice of basis set only has a limited impact on the results (Table S4-S5).

Based on a Wigner distribution around the ground state minimum geometry of DCM_o-I at 300 K, we calculated the absorption spectrum of the molecule at the B3LYP/aug-cc-pVDZ/cc-pVTZ-DK (iodine)/CPCM(DMSO) level and present it in Figure S14. The experimental and theoretical spectra are normalized to the intensity of the brightest peak, corresponding to the S0-S1 absorption. The sticks corresponding to the absorption in the minimum geometry are also shown in the figure, and a decomposition of the spectrum is given in terms of the different singlet states. Because S3 and S4 are very close in energy, their order is often inverted with distortion of the geometry through the Wigner distribution. One of them, nevertheless, is always dark while this other one is bright. The contribution of the third bright state is therefore referred to as S3/4. Considering vibrational distortion at 300 K broadens and red shifts all peaks, resulting in a predicted spectrum in fairly good agreement with the experimental one.

Table S4 Benchmark of the energy (in eV) of the first 6 singlet states of DCM_O-I. The basis set is given for H, C, N, O, and that of iodine was (aug-)cc-pVTZ-DK in all cases, with diffuse functions added consistently with the main basis set. For aug-cc-pVDZ*, diffuse functions on iodine were omitted.The time is the user time on 4 cores on Intel(R) Xeon(R) Gold 6258R CPU@2.70GHz. The oscillator strength for each state is given in parenthesis. For comparison, the first three bright states of DCM_O-I in DMSO are found experimentally at 1.9 eV, 2.9 eV, and 3.1 eV. "g.p." stands for gas phase.

DFT	Basis set	Solvent	S1	S2	S3	S4	S5	S6	Time (min)
bp86	cc-pVDZ	g.p.	1.809(0.00)	1.910(0.43)	2.551(0.62)	2.666(0.00)	2.674(0.05)	2.817(0.22)	4.5
bp86	cc-pVTZ	g.p.	1.851(0.00)	1.901(0.46)	2.539(0.53)	2.615(0.16)	2.719(0.00)	2.796(0.20)	9.2
bp86	aug-cc-pVDZ	g.p.	1.872(0.00)	1.895(0.49)	2.525(0.54)	2.604(0.16)	2.740(0.00)	2.762(0.22)	12.8
bp86	aug-cc-pVDZ*	g.p.	1.868(0.00)	1.892(0.49)	2.522(0.51)	2.596(0.18)	2.735(0.00)	2.757(0.22)	10.4
bp86	aug-cc-pVTZ	g.p.	1.880(0.00)	1.894(0.50)	2.525(0.51)	2.597(0.19)	2.750(0.00)	2.763(0.21)	30.6
bp86	cc-pVDZ	DMSO	1.968(1.02)	2.153(0.00)	2.673(0.60)	2.831(0.02)	2.948(0.19)	3.163(0.00)	5.2
bp86	cc-pVTZ	DMSO	1.951(1.06)	2.245(0.00)	2.674(0.58)	2.770(0.01)	2.914(0.21)	3.114(0.00)	10.4
bp86	aug-cc-pVDZ	DMSO	1.941(1.11)	2.297(0.00)	2.669(0.55)	2.762(0.01)	2.899(0.22)	3.109(0.00)	11.1
bp86	aug-cc-pVDZ*	DMSO	1.940(1.10)	2.294(0.00)	2.667(0.55)	2.754(0.02)	2.898(0.22)	3.102(0.00)	12.6
bp86	aug-cc-pVTZ	DMSO	1.940(1.11)	2.305(0.00)	2.668(0.55)	2.755(0.02)	2.897(0.22)	3.095(0.00)	31.5
pbe	cc-pVDZ	g.p.	1.782(0.00)	1.907(0.43)	2.549(0.61)	2.638(0.00)	2.668(0.06)	2.818(0.22)	4.6
pbe	cc-pVTZ	g.p.	1.823(0.00)	1.899(0.46)	2.536(0.50)	2.610(0.19)	2.691(0.00)	2.798(0.21)	9.5
pbe	aug-cc-pVDZ	g.p.	1.847(0.00)	1.892(0.49)	2.522(0.52)	2.599(0.17)	2.713(0.00)	2.757(0.23)	14.1
pbe	aug-cc-pVDZ*	g.p.	1.844(0.00)	1.889(0.48)	2.517(0.48)	2.590(0.21)	2.708(0.00)	2.751(0.23)	11.6
pbe	aug-cc-pVTZ	g.p.	1.854(0.00)	1.890(0.49)	2.520(0.48)	2.591(0.22)	2.722(0.00)	2.756(0.22)	33.4
pbe	cc-pVDZ	DMSO	1.966(1.01)	2.123(0.00)	2.670(0.61)	2.823(0.02)	2.947(0.20)	3.131(0.00)	5.0
pbe	cc-pVTZ	DMSO	1.949(1.05)	2.211(0.00)	2.673(0.58)	2.762(0.01)	2.915(0.21)	3.096(0.00)	10.2
pbe	aug-cc-pVDZ	DMSO	1.939(1.10)	2.268(0.00)	2.666(0.56)	2.754(0.02)	2.898(0.22)	3.093(0.00)	12.3
pbe	aug-cc-pVDZ*	DMSO	1.938(1.09)	2.265(0.00)	2.664(0.56)	2.745(0.02)	2.897(0.22)	3.083(0.00)	11.3
pbe	aug-cc-pVTZ	DMSO	1.937(1.10)	2.274(0.00)	2.665(0.55)	2.745(0.02)	2.896(0.22)	3.074(0.00)	46.3
tpss	cc-pVDZ	g.p.	1.940(0.00)	1.975(0.49)	2.645(0.72)	2.788(0.04)	2.842(0.00)	2.921(0.16)	4.9
tpss	cc-pVTZ	g.p.	1.969(0.52)	1.983(0.00)	2.638(0.68)	2.731(0.08)	2.898(0.00)	2.904(0.15)	11.5
tpss	aug-cc-pVDZ	g.p.	1.960(0.55)	2.000(0.00)	2.620(0.69)	2.720(0.08)	2.862(0.16)	2.912(0.00)	14.8

tpss	aug-cc-pVDZ*	g.p.	1.958(0.54)	1.997(0.00)	2.617(0.67)	2.711(0.09)	2.857(0.17)	2.907(0.00)	13.7
tpss	aug-cc-pVTZ	g.p.	1.959(0.55)	2.009(0.00)	2.622(0.67)	2.710(0.10)	2.863(0.16)	2.923(0.00)	36.7
tpss	cc-pVDZ	DMSO	2.026(1.10)	2.293(0.00)	2.760(0.60)	2.950(0.06)	3.049(0.17)	3.295(0.00)	5.2
tpss	cc-pVTZ	DMSO	2.011(1.14)	2.386(0.00)	2.764(0.57)	2.902(0.02)	3.012(0.21)	3.300(0.00)	11.9
tpss	aug-cc-pVDZ	DMSO	1.999(1.18)	2.433(0.00)	2.755(0.54)	2.893(0.02)	2.993(0.22)	3.279(0.00)	14.5
tpss	aug-cc-pVDZ*	DMSO	1.999(1.17)	2.431(0.00)	2.754(0.54)	2.886(0.02)	2.991(0.22)	3.291(0.00)	15.3
tpss	aug-cc-pVTZ	DMSO	1.999(1.18)	2.440(0.00)	2.757(0.55)	2.884(0.02)	2.992(0.22)	3.276(0.00)	45.5
m06L	cc-pVDZ	g.p.	2.056(0.54)	2.141(0.00)	2.756(0.65)	2.842(0.15)	3.055(0.16)	3.082(0.00)	5.3
m06L	cc-pVTZ	g.p.	2.045(0.57)	2.186(0.00)	2.741(0.60)	2.806(0.22)	3.029(0.00)	3.034(0.13)	11.3
m06L	aug-cc-pVDZ	g.p.	2.034(0.59)	2.188(0.00)	2.719(0.45)	2.778(0.36)	2.971(0.00)	2.993(0.15)	20.5
m06L	aug-cc-pVDZ*	g.p.	2.033(0.58)	2.186(0.00)	2.718(0.42)	2.776(0.37)	2.969(0.00)	2.990(0.15)	12.4
m06L	aug-cc-pVTZ	g.p.	2.033(0.60)	2.210(0.00)	2.722(0.56)	2.784(0.26)	2.997(0.13)	2.998(0.00)	36.2
m06L	cc-pVDZ	DMSO	2.103(1.14)	2.488(0.00)	2.886(0.65)	2.997(0.01)	3.172(0.21)	3.330(0.00)	5.6
m06L	cc-pVTZ	DMSO	2.087(1.19)	2.598(0.00)	2.878(0.59)	2.979(0.02)	3.139(0.22)	3.305(0.00)	12.8
m06L	aug-cc-pVDZ	DMSO	2.071(1.20)	2.595(0.00)	2.863(0.55)	2.937(0.06)	3.127(0.21)	3.224(0.00)	16.4
m06L	aug-cc-pVDZ*	DMSO	2.072(1.20)	2.595(0.00)	2.863(0.55)	2.936(0.07)	3.127(0.21)	3.225(0.00)	13.9
m06L	aug-cc-pVTZ	DMSO	2.073(1.23)	2.646(0.00)	2.865(0.55)	2.960(0.04)	3.121(0.22)	3.276(0.00)	53.4
b3lyp	cc-pVDZ	g.p.	2.268(0.95)	2.817(0.00)	3.005(0.69)	3.304(0.07)	3.451(0.06)	3.507(0.09)	11.9
b3lyp	cc-pVTZ	g.p.	2.248(0.97)	2.853(0.00)	2.993(0.67)	3.254(0.05)	3.419(0.04)	3.451(0.11)	31.2
b3lyp	aug-cc-pVDZ	g.p.	2.229(1.02)	2.867(0.00)	2.969(0.64)	3.235(0.05)	3.348(0.03)	3.385(0.00)	35.1
b3lyp	aug-cc-pVDZ*	g.p.	2.227(1.00)	2.862(0.00)	2.966(0.64)	3.228(0.05)	3.341(0.03)	3.426(0.11)	61.0
b3lyp	aug-cc-pVTZ	g.p.	2.227(1.01)	2.875(0.00)	2.970(0.64)	3.229(0.05)	3.350(0.03)	3.399(0.00)	274.9
b3lyp	cc-pVDZ	DMSO	2.225(1.50)	3.189(0.00)	3.199(0.41)	3.361(0.17)	3.579(0.00)	3.610(0.03)	9.4
b3lyp	cc-pVTZ	DMSO	2.200(1.51)	3.196(0.40)	3.275(0.00)	3.336(0.16)	3.521(0.05)	3.663(0.00)	26.3
b3lyp	aug-cc-pVDZ	DMSO	2.181(1.55)	3.185(0.38)	3.317(0.16)	3.321(0.00)	3.500(0.04)	3.555(0.00)	41.8
b3lyp	aug-cc-pVDZ*	DMSO	2.181(1.54)	3.184(0.39)	3.315(0.16)	3.317(0.00)	3.491(0.04)	3.641(0.00)	32.1
b3lyp	aug-cc-pVTZ	DMSO	2.181(1.55)	3.185(0.38)	3.316(0.16)	3.329(0.00)	3.491(0.04)	3.574(0.00)	202.8
pbe0	cc-pVDZ	g.p.	2.351(1.08)	3.000(0.00)	3.139(0.63)	3.439(0.08)	3.655(0.06)	3.665(0.06)	9.7

pbe0	cc-pVTZ	g.p.	2.331(1.10)	3.029(0.00)	3.131(0.60)	3.400(0.07)	3.610(0.10)	3.630(0.02)	29.1
pbe0	aug-cc-pVDZ	g.p.	2.311(1.14)	3.045(0.00)	3.108(0.58)	3.381(0.07)	3.555(0.02)	3.593(0.09)	44.9
pbe0	aug-cc-pVDZ*	g.p.	2.311(1.13)	3.040(0.00)	3.105(0.58)	3.377(0.07)	3.549(0.02)	3.588(0.09)	34.1
pbe0	aug-cc-pVTZ	g.p.	2.310(1.14)	3.047(0.00)	3.109(0.58)	3.376(0.07)	3.557(0.02)	3.587(0.09)	173.7
pbe0	cc-pVDZ	DMSO	2.290(1.58)	3.350(0.38)	3.371(0.00)	3.483(0.17)	3.764(0.04)	3.918(0.00)	10.9
pbe0	cc-pVTZ	DMSO	2.267(1.60)	3.343(0.37)	3.450(0.00)	3.464(0.17)	3.694(0.04)	3.977(0.00)	33.9
pbe0	aug-cc-pVDZ	DMSO	2.249(1.62)	3.329(0.36)	3.448(0.18)	3.492(0.00)	3.673(0.04)	3.865(0.00)	36.9
pbe0	aug-cc-pVDZ*	DMSO	2.249(1.62)	3.328(0.36)	3.446(0.17)	3.489(0.00)	3.667(0.04)	3.945(0.00)	33.5
pbe0	aug-cc-pVTZ	DMSO	2.249(1.62)	3.329(0.36)	3.448(0.17)	3.497(0.00)	3.666(0.04)	3.884(0.00)	216.7
tpssh	cc-pVDZ	g.p.	2.163(0.73)	2.439(0.00)	2.869(0.79)	3.104(0.03)	3.243(0.08)	3.300(0.16)	11.6
tpssh	cc-pVTZ	g.p.	2.151(0.77)	2.476(0.00)	2.863(0.77)	3.054(0.03)	3.223(0.06)	3.267(0.16)	30.2
tpssh	aug-cc-pVDZ	g.p.	2.138(0.81)	2.490(0.00)	2.842(0.76)	3.039(0.03)	3.170(0.06)	3.245(0.14)	57.9
tpssh	aug-cc-pVDZ*	g.p.	2.136(0.79)	2.487(0.00)	2.840(0.75)	3.032(0.04)	3.164(0.06)	3.243(0.14)	41.4
tpssh	aug-cc-pVTZ	g.p.	2.137(0.80)	2.497(0.00)	2.844(0.76)	3.031(0.03)	3.172(0.06)	3.243(0.14)	97.5
tpssh	cc-pVDZ	DMSO	2.162(1.35)	2.800(0.00)	3.020(0.50)	3.219(0.15)	3.368(0.07)	3.583(0.00)	10.5
tpssh	cc-pVTZ	DMSO	2.144(1.38)	2.889(0.00)	3.022(0.47)	3.196(0.13)	3.305(0.09)	3.654(0.00)	28.5
tpssh	aug-cc-pVDZ	DMSO	2.128(1.41)	2.931(0.00)	3.012(0.45)	3.182(0.14)	3.286(0.09)	3.550(0.00)	47.5
tpssh	aug-cc-pVDZ*	DMSO	2.128(1.41)	2.928(0.00)	3.011(0.45)	3.179(0.13)	3.281(0.09)	3.627(0.00)	31.9
tpssh	aug-cc-pVTZ	DMSO	2.128(1.41)	2.937(0.00)	3.014(0.45)	3.180(0.13)	3.279(0.10)	3.576(0.00)	96.5

Table S5 Table S4 Continued

DFT	Basis set	solvent	S1	S2	S3	S4	S5	S6	time (min)
m062x	cc-pVDZ	g.p.	2.503(1.53)	3.605(0.28)	3.678(0.00)	3.870(0.20)	4.076(0.00)	4.316(0.01)	12.6
m062x	cc-pVTZ	g.p.	2.478(1.54)	3.603(0.26)	3.735(0.00)	3.845(0.20)	4.150(0.00)	4.252(0.04)	39.8
m062x	aug-cc-pVDZ	g.p.	2.445(1.54)	3.543(0.24)	3.671(0.00)	3.711(0.00)	3.809(0.21)	3.917(0.00)	59.1
m062x	aug-cc-pVDZ*	g.p.	2.446(1.53)	3.539(0.24)	3.666(0.00)	3.707(0.00)	3.810(0.21)	3.922(0.00)	56.1
m062x	aug-cc-pVTZ	g.p.	2.450(1.55)	3.569(0.23)	3.722(0.00)	3.750(0.00)	3.819(0.21)	3.964(0.00)	431.4

m062x	cc-pVDZ	DMSO	2.378(1.81)	3.750(0.26)	3.929(0.21)	4.053(0.00)	4.140(0.00)	4.386(0.11)	16.7
m062x	cc-pVTZ	DMSO	2.354(1.81)	3.717(0.27)	3.934(0.19)	4.163(0.00)	4.236(0.00)	4.309(0.09)	39.2
m062x	aug-cc-pVDZ	DMSO	2.329(1.82)	3.675(0.27)	3.892(0.18)	4.066(0.00)	4.161(0.00)	4.213(0.13)	60.4
m062x	aug-cc-pVDZ*	DMSO	2.329(1.82)	3.675(0.27)	3.890(0.18)	4.122(0.00)	4.160(0.00)	4.233(0.12)	52.3
m062x	aug-cc-pVTZ	DMSO	2.333(1.83)	3.686(0.27)	3.916(0.18)	4.124(0.00)	4.208(0.00)	4.228(0.12)	160.5
cam-b3lyp	cc-pVDZ	g.p.	2.551(1.57)	3.699(0.24)	3.734(0.00)	3.895(0.21)	4.147(0.00)	4.354(0.04)	17.5
cam-b3lyp	cc-pVTZ	g.p.	2.519(1.57)	3.679(0.23)	3.772(0.00)	3.866(0.22)	4.200(0.00)	4.266(0.05)	43.2
cam-b3lyp	aug-cc-pVDZ	g.p.	2.488(1.60)	3.645(0.20)	3.783(0.00)	3.840(0.23)	3.940(0.00)	4.096(0.00)	49.6
cam-b3lyp	aug-cc-pVDZ*	g.p.	2.488(1.58)	3.640(0.20)	3.777(0.00)	3.840(0.23)	3.977(0.00)	4.191(0.00)	37.9
cam-b3lyp	aug-cc-pVTZ	g.p.	2.488(1.59)	3.646(0.20)	3.791(0.00)	3.840(0.23)	3.940(0.00)	4.082(0.00)	275.4
cam-b3lyp	cc-pVDZ	DMSO	2.433(1.84)	3.780(0.26)	4.028(0.19)	4.143(0.00)	4.202(0.00)	4.389(0.10)	14.9
cam-b3lyp	cc-pVTZ	DMSO	2.404(1.84)	3.742(0.27)	4.012(0.18)	4.237(0.00)	4.279(0.00)	4.310(0.10)	45.3
cam-b3lyp	aug-cc-pVDZ	DMSO	2.380(1.85)	3.709(0.27)	3.995(0.16)	4.160(0.00)	4.236(0.12)	4.282(0.00)	66.0
cam-b3lyp	aug-cc-pVDZ*	DMSO	2.380(1.85)	3.710(0.28)	3.992(0.16)	4.234(0.00)	4.252(0.10)	4.278(0.00)	68.2
cam-b3lyp	aug-cc-pVTZ	DMSO	2.381(1.85)	3.709(0.27)	3.994(0.16)	4.179(0.00)	4.231(0.12)	4.289(0.00)	250.3
wb97x-d3bj	cc-pVDZ	g.p.	2.621(1.75)	4.002(0.13)	4.120(0.00)	4.279(0.25)	4.501(0.00)	4.561(0.10)	17.3
wb97x-d3bj	cc-pVTZ	g.p.	2.590(1.74)	3.969(0.13)	4.156(0.00)	4.253(0.23)	4.483(0.11)	4.532(0.00)	36.7
wb97x-d3bj	aug-cc-pVDZ	g.p.	2.555(1.75)	3.932(0.12)	4.168(0.00)	4.223(0.20)	4.280(0.00)	4.368(0.15)	48.4
wb97x-d3bj	aug-cc-pVDZ*	g.p.	2.556(1.74)	3.931(0.12)	4.163(0.00)	4.221(0.21)	4.306(0.00)	4.392(0.13)	49.8
wb97x-d3bj	aug-cc-pVTZ	g.p.	2.558(1.75)	3.932(0.12)	4.171(0.00)	4.224(0.19)	4.261(0.00)	4.364(0.15)	483.5
wb97x-d3bj	cc-pVDZ	DMSO	2.506(1.94)	3.937(0.26)	4.443(0.09)	4.536(0.24)	4.536(0.00)	4.561(0.00)	19.2
wb97x-d3bj	cc-pVTZ	DMSO	2.479(1.93)	3.896(0.27)	4.389(0.04)	4.480(0.26)	4.609(0.00)	4.630(0.00)	42.4
wb97x-d3bj	aug-cc-pVDZ	DMSO	2.455(1.94)	3.860(0.28)	4.332(0.00)	4.430(0.29)	4.503(0.00)	4.671(0.00)	72.5
wb97x-d3bj	aug-cc-pVDZ*	DMSO	2.455(1.94)	3.860(0.28)	4.341(0.01)	4.439(0.27)	4.562(0.00)	4.668(0.00)	67.3
wb97x-d3bj	aug-cc-pVTZ	DMSO	2.458(1.94)	3.861(0.28)	4.328(0.00)	4.426(0.29)	4.517(0.00)	4.676(0.00)	249.5
RSX-0DH	cc-pVDZ	g.p.	2.688(1.80)	4.175(0.13)	4.371(0.00)	4.459(0.23)	4.746(0.13)	4.879(0.00)	28.2
RSX-0DH	cc-pVTZ	g.p.	2.653(1.79)	4.130(0.13)	4.426(0.19)	4.388(0.00)	4.649(0.17)	4.900(0.00)	92.3
RSX-0DH	aug-cc-pVDZ	g.p.	2.624(1.79)	4.095(0.12)	4.439(0.04)	4.495(0.01)	4.508(0.31)	4.724(0.00)	90.4

RSX-0DH	aug-cc-pVDZ*	g.p.	2.625(1.79)	4.093(0.12)	4.405(0.10)	4.525(0.00)	4.541(0.25)	4.400(0.01)	82.2
RSX-0DH	aug-cc-pVTZ	g.p.	2.623(1.79)	4.093(0.12)	4.513(0.00)	4.419(0.04)	4.501(0.32)	4.395(0.00)	346.4
RSX-0DH	cc-pVDZ	DMSO	2.576(1.96)	4.100(0.24)	4.629(0.03)	4.708(0.33)	4.955(0.00)	4.821(0.00)	30.5
RSX-0DH	cc-pVTZ	DMSO	2.546(1.95)	4.051(0.25)	4.559(0.01)	4.637(0.34)	4.992(0.00)	4.891(0.00)	95.9
RSX-0DH	aug-cc-pVDZ	DMSO	2.525(1.96)	4.018(0.26)	4.495(0.01)	4.593(0.33)	4.840(0.00)	5.106(0.00)	102.7
RSX-0DH	aug-cc-pVDZ*	DMSO	2.525(1.95)	4.018(0.26)	4.507(0.00)	4.600(0.32)	4.893(0.00)	5.133(0.00)	95.5
RSX-0DH	aug-cc-pVTZ	DMSO	2.525(1.95)	4.017(0.26)	4.491(0.01)	4.586(0.32)	4.861(0.00)	5.109(0.00)	336.4
wPBEPP86	cc-pVDZ	g.p.	2.326(1.56)	3.677(0.11)	3.742(0.22)	3.586(0.00)	4.351(0.10)	4.573(0.00)	23.0
wPBEPP86	cc-pVTZ	g.p.	2.280(1.53)	3.616(0.11)	3.705(0.19)	4.221(0.13)	3.569(0.00)	4.618(0.00)	87.7
wPBEPP86	aug-cc-pVDZ	g.p.	2.261(1.54)	3.586(0.10)	4.111(0.00)	3.771(0.09)	4.031(0.25)	4.367(0.00)	72.6
wPBEPP86	aug-cc-pVDZ*	g.p.	2.261(1.53)	3.583(0.10)	4.115(0.00)	3.733(0.13)	4.087(0.20)	4.370(0.00)	88.6
wPBEPP86	aug-cc-pVTZ	g.p.	2.248(1.53)	3.578(0.10)	4.174(0.00)	3.787(0.08)	4.008(0.26)	4.407(0.01)	551.4
wPBEPP86	cc-pVDZ	DMSO	2.208(1.67)	3.608(0.23)	4.078(0.11)	4.289(0.22)	4.648(0.00)	4.691(0.06)	22.3
wPBEPP86	cc-pVTZ	DMSO	2.171(1.65)	3.539(0.23)	4.037(0.05)	4.172(0.26)	4.707(0.00)	4.982(0.00)	105.5
wPBEPP86	aug-cc-pVDZ	DMSO	2.161(1.67)	3.515(0.24)	4.029(0.00)	4.087(0.30)	4.501(0.00)	4.928(0.00)	122.2
wPBEPP86	aug-cc-pVDZ*	DMSO	2.160(1.66)	3.516(0.24)	4.022(0.01)	4.111(0.28)	4.560(0.00)	4.927(0.00)	103.0
wPBEPP86	aug-cc-pVTZ	DMSO	2.149(1.65)	3.505(0.24)	4.026(0.00)	4.071(0.30)	4.554(0.00)	4.794(0.00)	590.7
wb97x-2	cc-pVDZ	g.p.	1.746(1.20)	2.970(0.10)	2.821(0.16)	4.059(0.00)	2.394(0.00)	3.805(0.10)	23.4
wb97x-2	cc-pVTZ	g.p.	1.686(1.17)	2.883(0.10)	2.797(0.14)	4.107(0.01)	3.680(0.11)	2.324(0.00)	92.0
wb97x-2	aug-cc-pVDZ	g.p.	1.674(1.18)	2.862(0.09)	3.529(0.00)	3.800(0.00)	3.178(0.02)	3.739(0.01)	91.7
wb97x-2	aug-cc-pVDZ*	g.p.	1.674(1.17)	2.857(0.09)	3.531(0.00)	3.207(0.04)	3.558(0.03)	3.335(0.21)	87.6
wb97x-2	aug-cc-pVTZ	g.p.	1.648(1.16)	3.711(0.00)	2.847(0.09)	3.907(0.00)	3.285(0.02)	3.828(0.01)	819.5
wb97x-2	cc-pVDZ	DMSO	1.599(1.24)	2.842(0.18)	3.425(0.07)	4.072(0.01)	3.660(0.20)	5.135(0.00)	29.3
wb97x-2	cc-pVTZ	DMSO	1.548(1.21)	2.743(0.18)	3.366(0.03)	3.517(0.23)	4.207(0.00)	4.937(0.00)	101.1
wb97x-2	aug-cc-pVDZ	DMSO	1.554(1.23)	2.726(0.19)	3.402(0.00)	3.952(0.00)	3.400(0.25)	4.205(0.00)	87.9
wb97x-2	aug-cc-pVDZ*	DMSO	1.551(1.22)	2.727(0.19)	3.384(0.00)	3.866(0.07)	3.597(0.19)	4.283(0.00)	87.2
wb97x-2	aug-cc-pVTZ	DMSO	1.526(1.21)	2.705(0.19)	3.401(0.00)	4.065(0.00)	3.378(0.25)	4.324(0.00)	316.0
SCS-wPBEPP86	cc-pVDZ	g.p.	2.119(1.42)	3.530(0.11)	3.589(0.21)	3.512(0.00)	4.188(0.10)	4.477(0.00)	25.2

SCS-wPBEPP86	cc-pVTZ	g.p.	2.060(1.38)	3.457(0.11)	3.540(0.19)	4.044(0.13)	3.492(0.00)	4.514(0.00)	95.9
SCS-wPBEPP86	aug-cc-pVDZ	g.p.	2.045(1.39)	3.433(0.10)	4.008(0.00)	3.611(0.09)	3.869(0.24)	4.271(0.00)	77.2
SCS-wPBEPP86	aug-cc-pVDZ*	g.p.	2.046(1.39)	3.430(0.10)	4.011(0.00)	3.574(0.13)	3.922(0.19)	4.273(0.00)	90.6
SCS-wPBEPP86	aug-cc-pVTZ	g.p.	2.026(1.38)	3.419(0.10)	4.063(0.00)	3.621(0.07)	3.841(0.25)	4.303(0.01)	569.8
SCS-wPBEPP86	cc-pVDZ	DMSO	1.980(1.50)	3.447(0.22)	3.909(0.10)	4.106(0.21)	4.552(0.00)	4.500(0.06)	22.7
SCS-wPBEPP86	cc-pVTZ	DMSO	1.929(1.47)	3.366(0.22)	3.852(0.05)	3.980(0.25)	4.600(0.00)	4.871(0.00)	110.3
SCS-wPBEPP86	aug-cc-pVDZ	DMSO	1.924(1.48)	3.346(0.23)	3.849(0.00)	3.908(0.29)	4.399(0.00)	4.825(0.00)	129.3
SCS-wPBEPP86	aug-cc-pVDZ*	DMSO	1.923(1.48)	3.347(0.23)	3.842(0.01)	3.930(0.27)	4.456(0.00)	4.824(0.00)	107.4
SCS-wPBEPP86	aug-cc-pVTZ	DMSO	1.906(1.47)	3.331(0.22)	3.838(0.00)	3.885(0.29)	4.445(0.00)	4.683(0.00)	601.9
SOS-wPBEPP86	cc-pVDZ	g.p.	2.123(1.42)	3.581(0.11)	3.683(0.22)	3.579(0.00)	4.202(0.10)	4.542(0.00)	23.9
SOS-wPBEPP86	cc-pVTZ	g.p.	2.066(1.39)	3.509(0.11)	3.631(0.19)	4.060(0.13)	3.559(0.00)	4.576(0.00)	101.2
SOS-wPBEPP86	aug-cc-pVDZ	g.p.	2.056(1.40)	3.491(0.10)	4.112(0.00)	3.690(0.09)	3.918(0.24)	4.361(0.00)	80.2
SOS-wPBEPP86	aug-cc-pVDZ*	g.p.	2.057(1.39)	3.489(0.10)	4.117(0.00)	3.660(0.13)	3.963(0.20)	4.387(0.00)	90.4
SOS-wPBEPP86	aug-cc-pVTZ	g.p.	2.032(1.38)	3.472(0.10)	4.168(0.00)	3.691(0.08)	3.890(0.25)	4.409(0.01)	563.0
SOS-wPBEPP86	cc-pVDZ	DMSO	1.983(1.50)	3.486(0.22)	3.960(0.11)	4.107(0.21)	4.618(0.00)	4.522(0.06)	23.7
SOS-wPBEPP86	cc-pVTZ	DMSO	1.933(1.47)	3.405(0.22)	3.888(0.05)	3.991(0.25)	4.661(0.00)	4.915(0.00)	103.6
SOS-wPBEPP86	aug-cc-pVDZ	DMSO	1.935(1.49)	3.389(0.23)	3.878(0.00)	3.947(0.29)	4.479(0.00)	4.882(0.00)	121.2
SOS-wPBEPP86	aug-cc-pVDZ*	DMSO	1.934(1.49)	3.390(0.23)	3.877(0.01)	3.962(0.27)	4.540(0.00)	4.881(0.00)	97.6
SOS-wPBEPP86	aug-cc-pVTZ	DMSO	1.911(1.47)	3.368(0.23)	3.861(0.00)	3.919(0.29)	4.525(0.00)	4.767(0.00)	574.6

Table S6 Error (eV) with respect to experimental observations for the prediction of energy differences between bright states of DCM$_o$-I as summary of the benchmark of density functionals for DCM$_o$-I. All calculations presented in the table were performed with the Douglas-Kroll-Hess Hamiltonian, aug-cc-pVDZ/cc-pVTZ-DK(iodine) basis set, in implicit DMSO on structures optimized with the same functional without diffuse basis function in the gas phase.

DFT	*Error on* $\boldsymbol{\Delta E_{S0-S1}}$	*Error on* $\boldsymbol{\Delta E_{S1-S2}}$	*Error on* $\boldsymbol{\Delta E_{S2-S3/4}}$*
B3LYP	0.281	0.003	-0.069
PBE0	0.349	0.079	-0.082
TPSSH	0.228	-0.117	-0.032
M06-2X	0.429	0.346	0.014
CAM-B3LYP	0.480	0.330	0.082
wB97X-D3BJ	0.555	0.405	0.378
RSX-0DH	0.625	0.494	0.382
wPBEPP86	0.260	0.356	0.395
wB97X-2	-0.349	0.176	0.670
SCS-wPBEPP86	0.023	0.424	0.382

* S3/4 refers to the third bright state. There is a dark singlet state in the same range of energy and the energy ordering varies from one method to another.

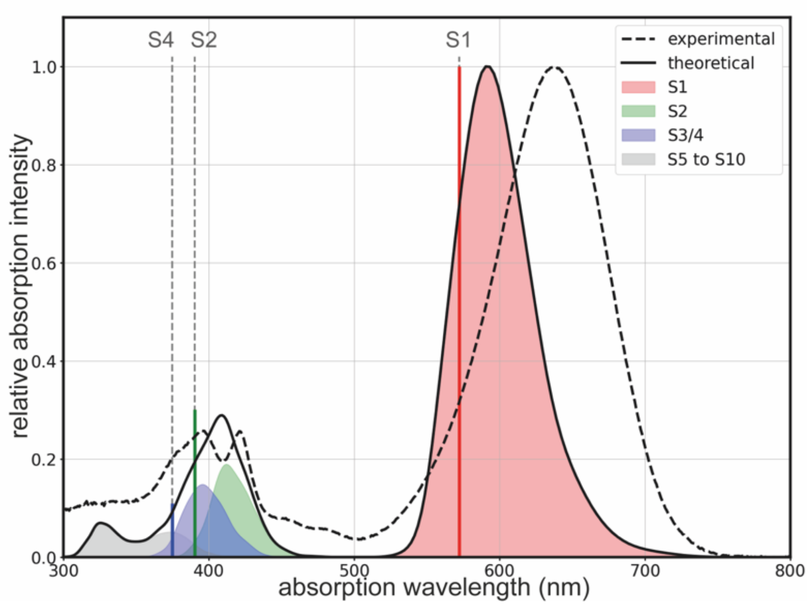

Figure S14 Experimental and calculated absorption spectrum of DCM$_O$-I in DMSO. The theoretical spectrum was calculated from a Wigner distribution at 300 K with B3LYP/aug-cc-pVDZ/cc-pVTZ-DK(iodine)/CPCM(DMSO). A decomposition of the spectrum into contributions of the different singlet state is given as filled curved. S3 (dark state) and S4 (bright state) are very close in energy and their order can be inverted from one geometry to another. Yet, one of them is always bright while the other is dark and the bright state is correspondingly labeled as S3/4. The line spectrum in the ground state optimized geometry is given as sticks.

Computational Studies – Additional Figures & Tables:

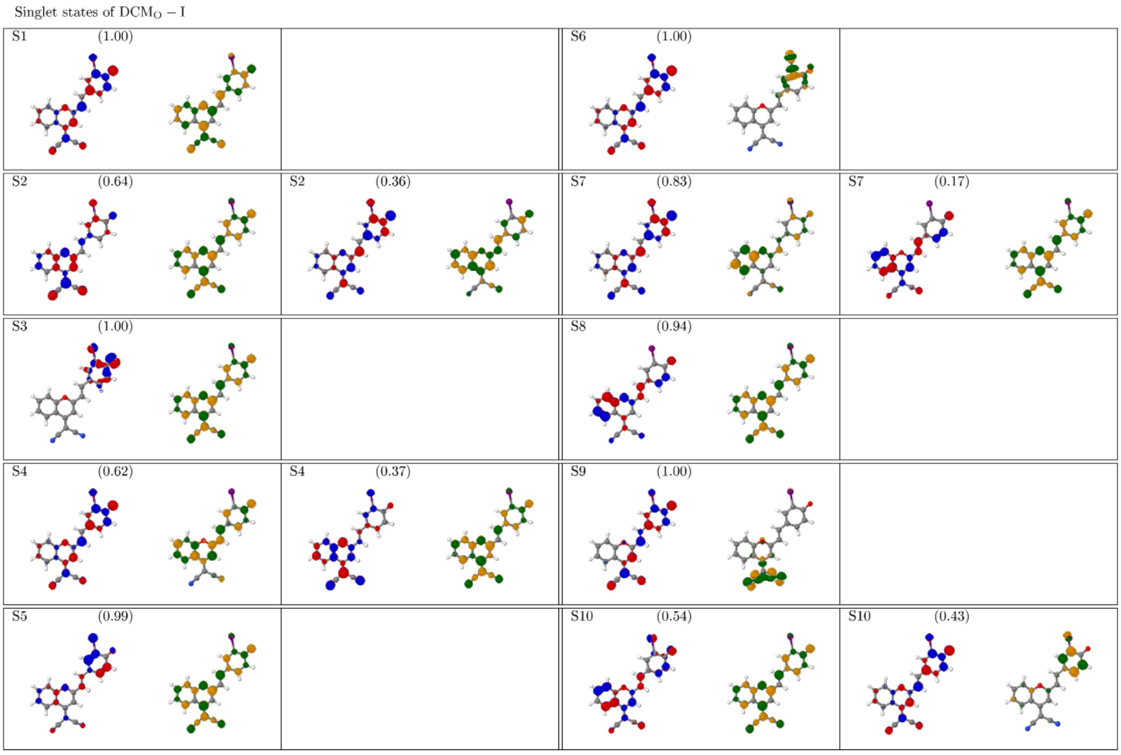

Figure S15 Natural transition orbitals for the first 10 singlet states of DCM$_O$-I.

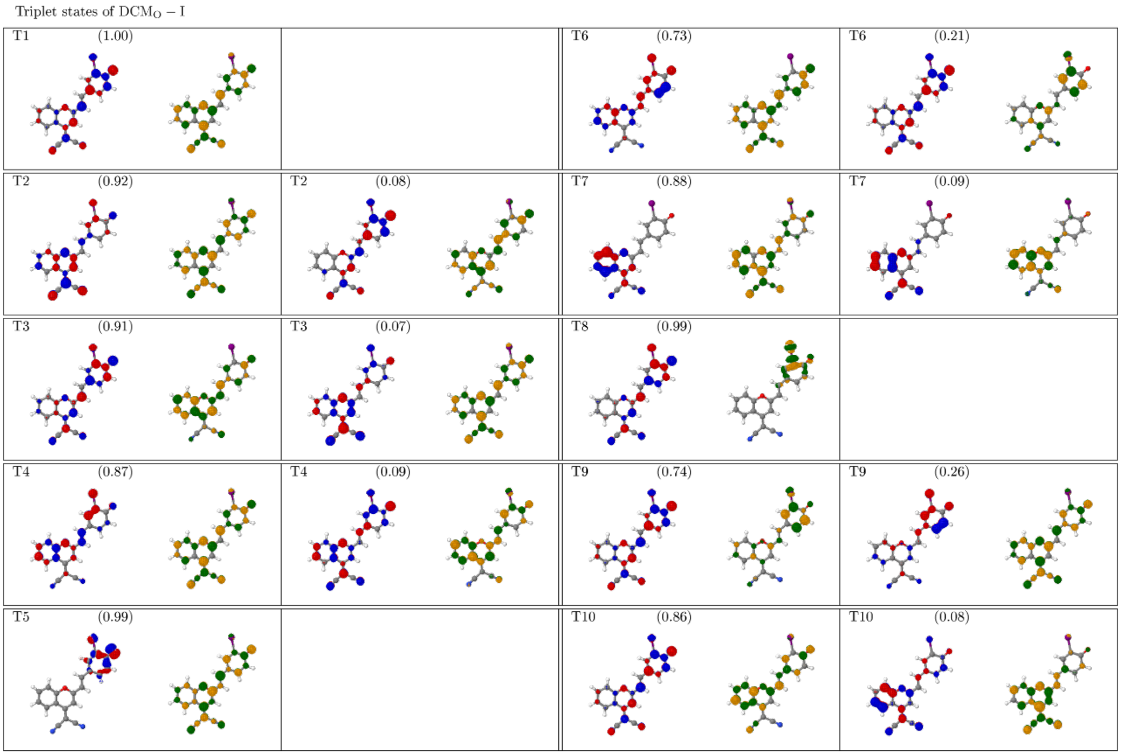

Figure S16 Natural transition orbitals for the first 10 triplet states of **DCM$_O$-I**.

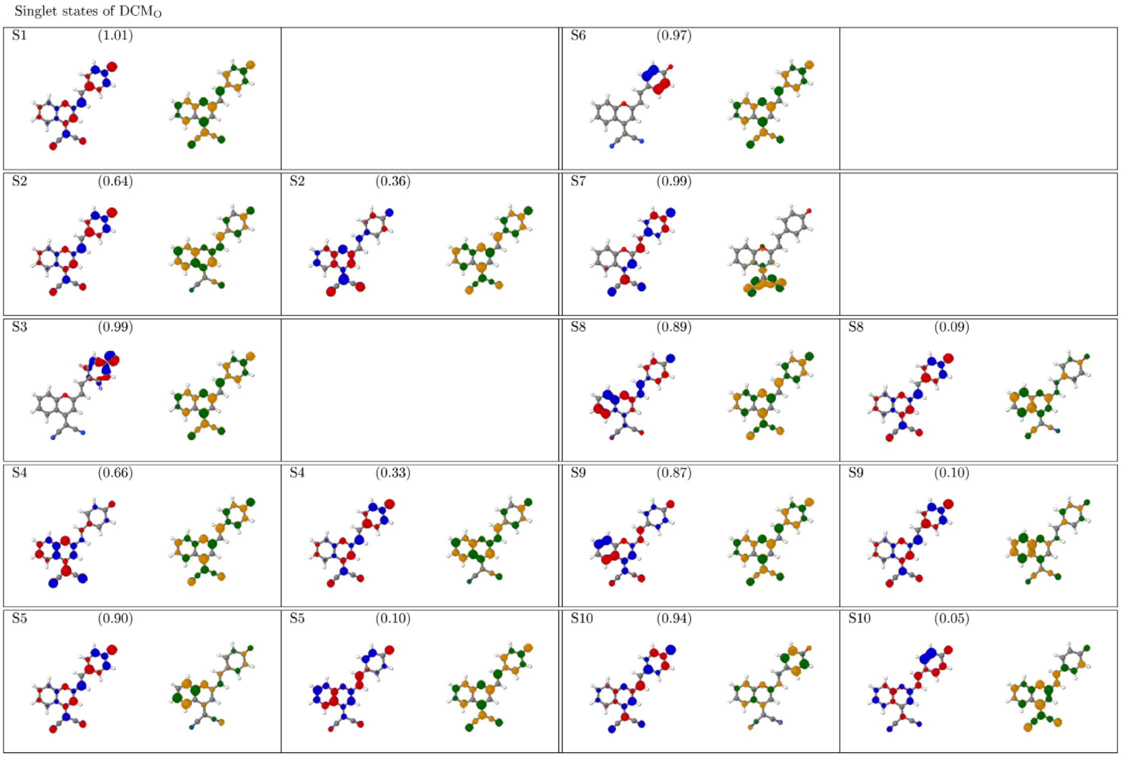

Figure S17 Natural transition orbitals for the first 10 singlet states of **DCM$_O$**.

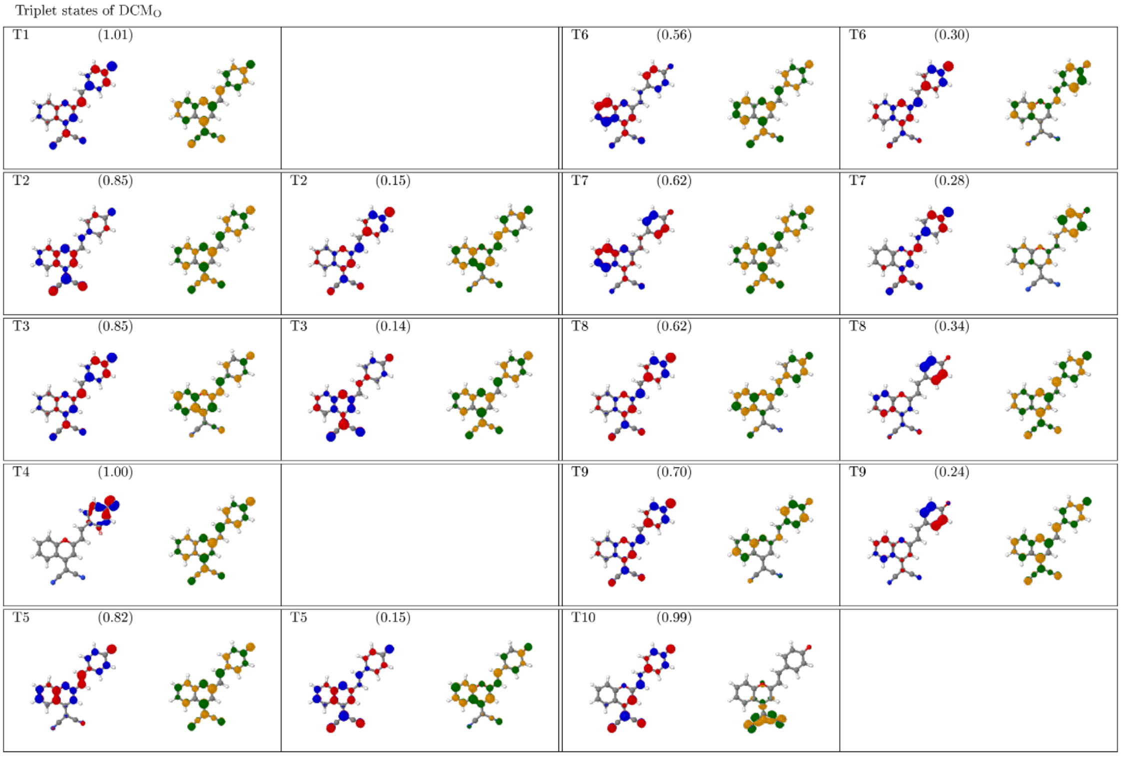

Figure S18 Natural transition orbitals for the first 10 triplet states of **DCM$_O$**.

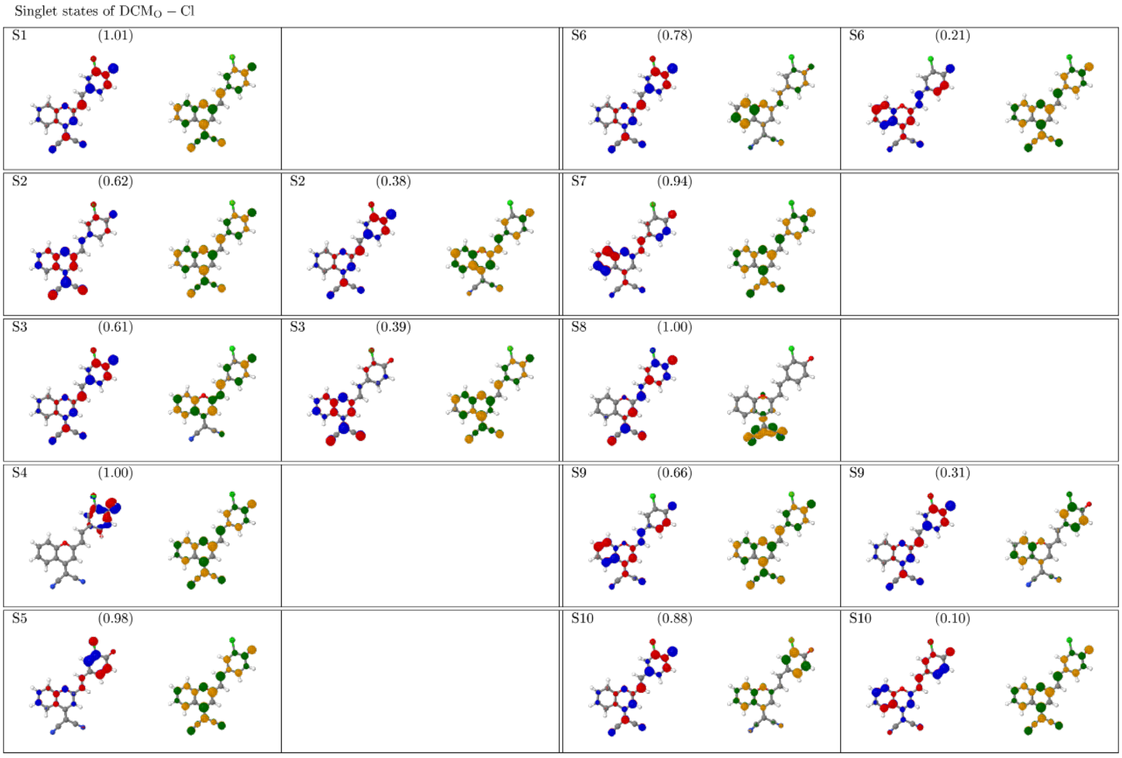

Figure S19 Natural transition orbitals for the first 10 singlet states of **DCM_O-Cl**.

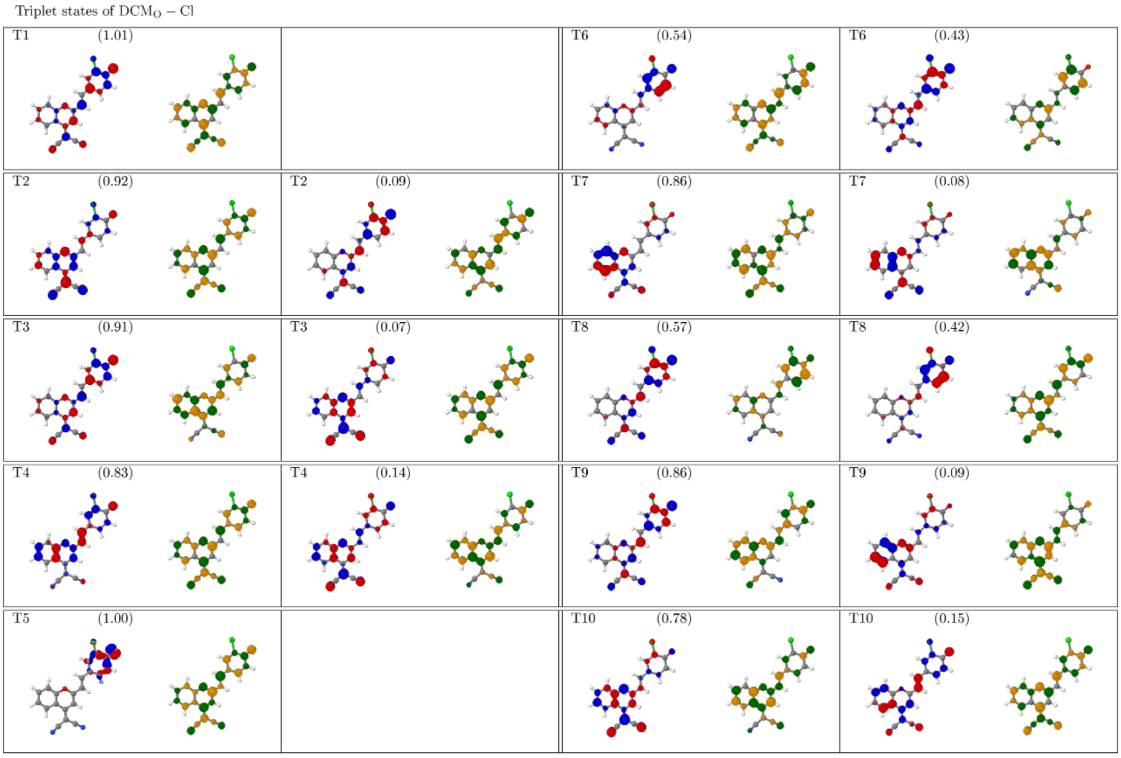

Figure S20 Natural transition orbitals for the first 10 triplet states of **DCM_O-Cl**.

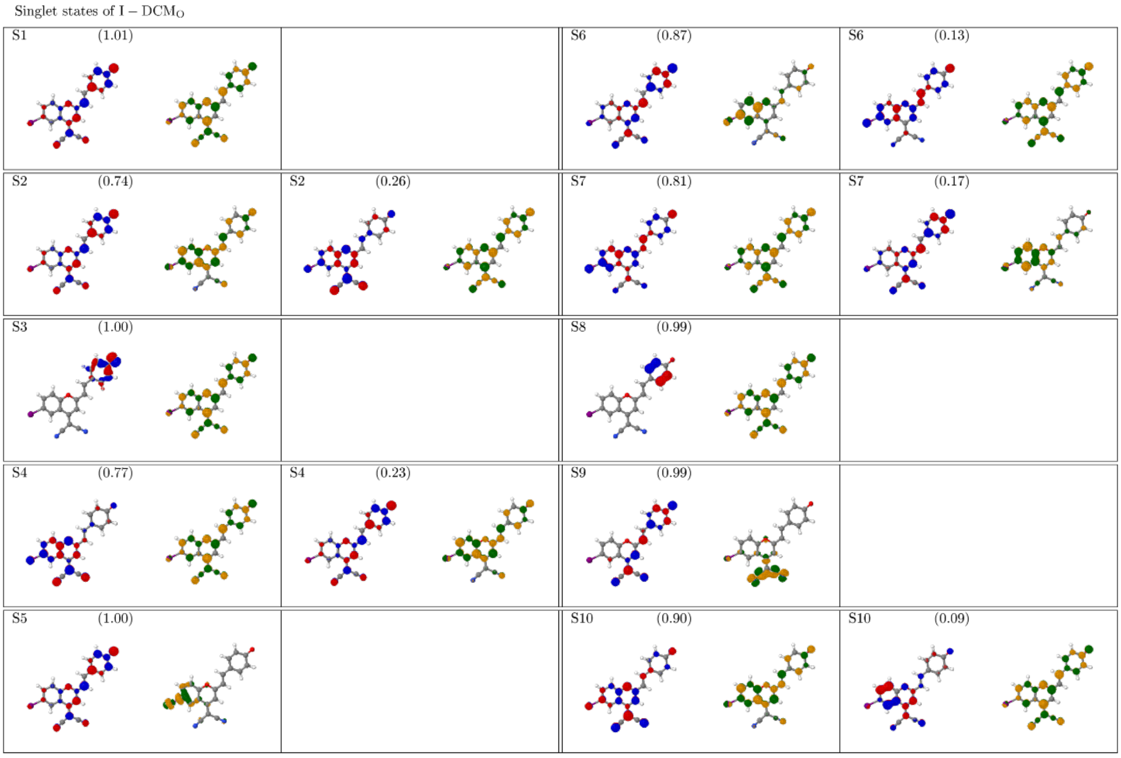

Figure S21 Natural transition orbitals for the first 10 singlet states of I-DCM$_O$.

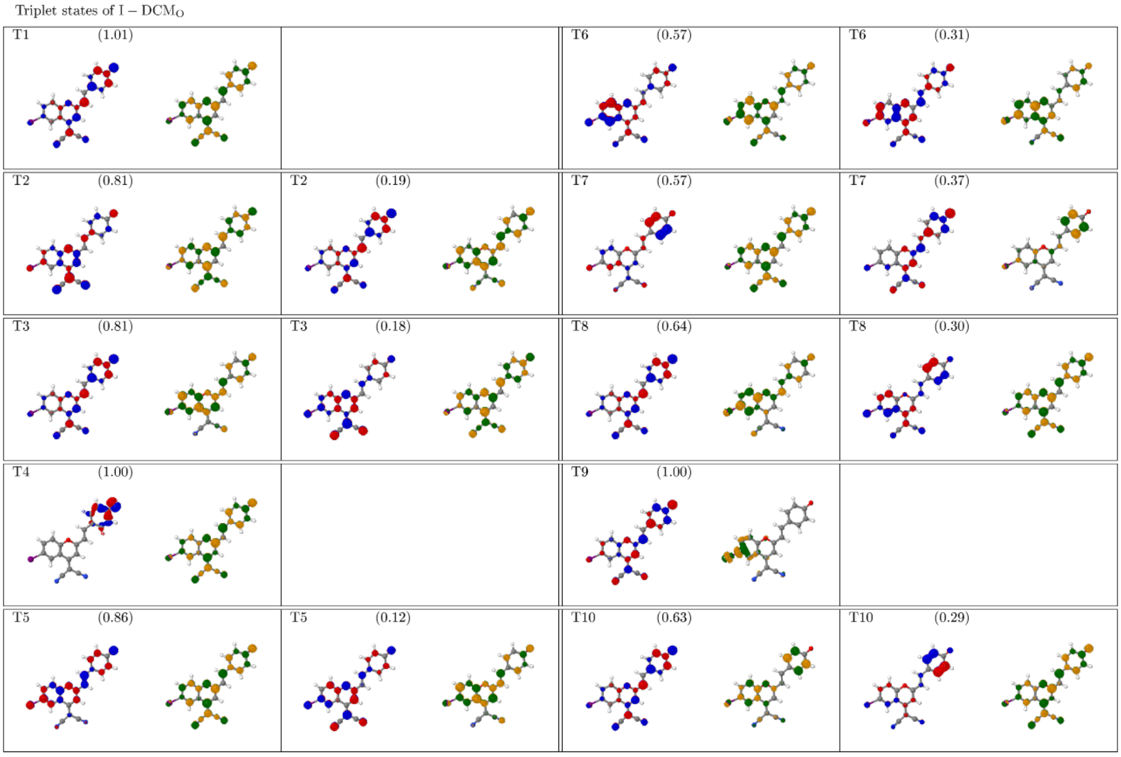

Figure S22 Natural transition orbitals for the first 10 triplet states of I-DCM$_O$.

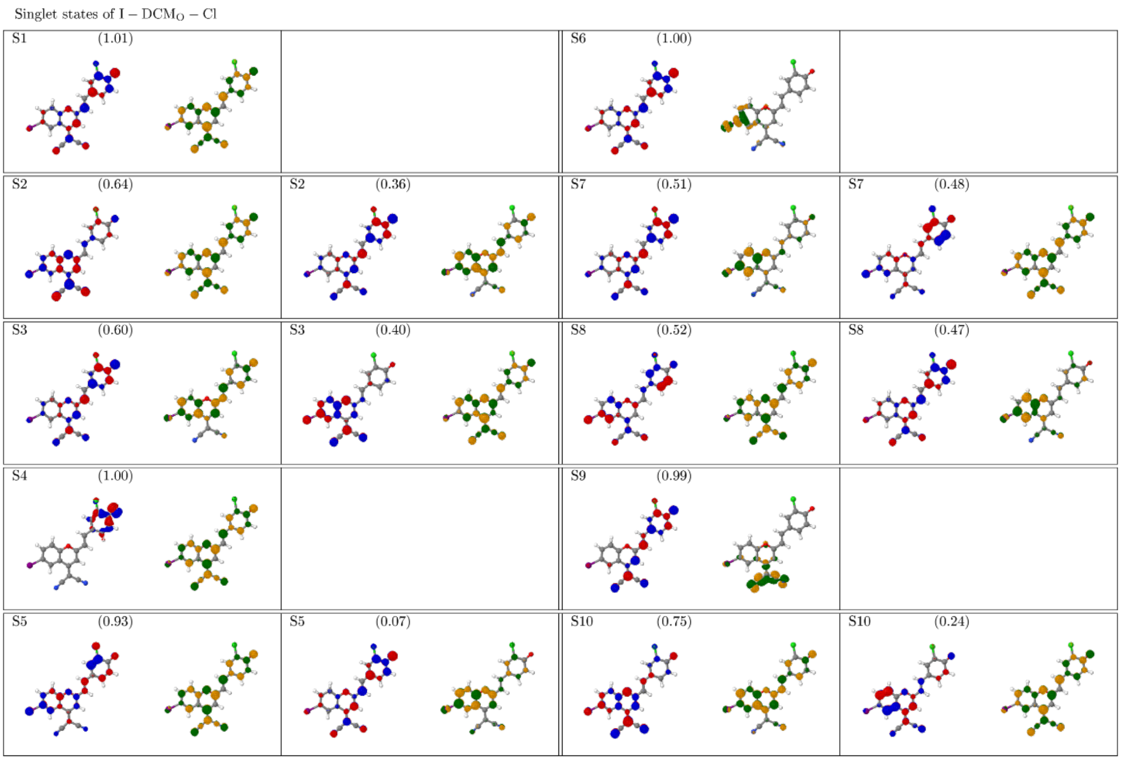

Figure S23 Natural transition orbitals for the first 10 singlet states of I-DCM$_O$-Cl.

Table S7 Square modulus of spin-orbit coupling matrix elements (in cm^{-1}; bold font) between singlet and triplet states of DCM$_O$-I. The energy of each state is also given in eV and the oscillator strength of the singlet states in indicated as "f".

	Singlet	0	1	2	3	4	5	6	7	8	9	10
	energy	0.00	2.17	3.18	3.26	3.31	3.46	3.63	3.88	3.93	3.97	4.00
	f	0.00	1.46	0.44	0.00	0.16	0.06	0.00	0.01	0.00	0.00	0.03
Triplet	energy											
1	1.42	0.51	0.04	0.93	148.02	3.48	2.67	47.27	0.78	2.03	3.92	94.70
2	2.35	0.84	0.13	0.73	111.91	2.78	2.25	4.10	0.44	1.45	3.84	71.24
3	2.67	2.30	0.19	0.36	60.38	1.45	1.24	34.13	0.22	0.98	2.97	44.89
4	3.10	10.30	26.44	17.66	422.26	41.79	87.88	53.62	4.19	2.53	5.80	269.11
5	3.11	41.28	174.95	135.90	80.62	208.17	530.72	9.62	20.04	26.75	3.36	149.11
6	3.21	10.97	1.29	2.58	239.96	4.37	1.58	103.39	0.89	3.62	4.18	159.78
7	3.30	4.22	0.33	0.60	57.17	1.02	0.32	19.95	0.44	0.83	1.01	36.60
8	3.41	883.15	3.49	24.77	34.01	15.21	44.69	19.95	49.83	10.40	4.64	89.85
9	3.44	91.44	1.78	5.22	279.12	5.56	3.67	183.76	4.10	3.84	4.16	181.62
10	3.67	6.50	0.25	0.57	43.32	0.71	0.25	16.23	0.30	0.74	0.13	31.42

Table S8. Square modulus of spin-orbit coupling matrix elements (in cm^{-1}; bold font) between singlet and triplet states of DCM$_O$. The energy of each state is also given in eV and the oscillator strength of the singlet states in indicated as "f".

	Singlet	0	1	2	3	4	5	6	7	8	9	10
	energy	0.00	2.18	3.16	3.16	3.31	3.82	3.89	3.89	3.95	4.05	4.10
	f	0.00	1.47	0.40	0.00	0.21	0.00	0.00	0.00	0.01	0.08	0.10
Triplet	energy											
1	1.35	0.75	0.02	1.89	19.76	0.10	0.24	0.37	4.96	0.04	0.05	0.05
2	2.38	0.12	0.07	1.05	10.64	0.06	0.11	0.29	3.34	0.05	0.03	0.06
3	2.63	2.07	0.14	0.11	1.02	0.11	0.08	0.22	3.17	0.08	0.07	0.17
4	2.98	25.93	17.94	1.25	0.12	7.93	3.29	1.91	0.16	12.01	8.34	2.41

5	3.16	0.22	0.06	1.36	14.32	0.07	0.05	0.05	0.80	0.02	0.04	0.07
6	3.31	1.16	0.09	0.04	0.50	0.09	0.06	0.02	0.38	0.05	0.06	0.03
7	3.36	1.09	0.05	0.21	2.08	0.05	0.04	0.02	0.54	0.06	0.07	0.01
8	3.57	0.42	0.06	0.27	1.60	0.03	0.05	0.05	0.71	0.03	0.04	0.12
9	3.70	0.57	0.09	0.27	2.39	0.05	0.04	0.04	0.36	0.02	0.04	0.08
10	3.79	13.05	5.82	0.86	0.38	3.72	0.21	0.04	0.25	0.67	1.08	0.89

Table S9 Square modulus of spin-orbit coupling matrix elements (in cm^{-1}; bold font) between singlet and triplet states of DCM$_O$-Cl. The energy of each state is also given in eV and the oscillator strength of the singlet states in indicated as "f".

	Singlet	0	1	2	3	4	5	6	7	8	9	10
	energy	0.00	2.18	3.18	3.32	3.34	3.70	3.89	3.93	3.98	4.02	4.08
	f	0.00	1.45	0.41	0.21	0.00	0.01	0.01	0.00	0.00	0.03	0.17
Triplet	energy											
1	1.41	0.50	0.02	0.04	0.07	17.17	0.05	0.21	0.09	4.87	0.13	0.05
2	2.35	0.46	0.06	0.10	0.06	8.86	0.08	0.10	0.10	2.97	0.05	0.02
3	2.67	1.98	0.15	0.08	0.13	1.00	0.02	0.11	0.06	3.65	0.16	0.09
4	3.15	0.51	0.07	0.04	0.09	11.00	0.02	0.07	0.03	0.69	0.03	0.07
5	3.17	24.73	15.65	2.27	5.57	0.04	11.96	4.78	9.52	0.57	8.39	3.78
6	3.26	0.64	0.10	0.04	0.10	9.97	0.04	0.04	0.04	0.11	0.03	0.02
7	3.30	1.35	0.05	0.05	0.05	1.95	0.04	0.04	0.03	0.69	0.09	0.05
8	3.51	0.52	0.05	0.05	0.04	9.68	0.01	0.01	0.04	0.15	0.03	0.02
9	3.68	0.07	0.09	0.09	0.04	3.13	0.02	0.06	0.03	0.75	0.04	0.06
10	3.79	1.46	0.09	0.11	0.04	3.58	0.03	0.04	0.05	0.71	0.01	0.06

Table S10 . Square modulus of spin-orbit coupling matrix elements (in cm^{-1}; bold font) between singlet and triplet states of I-DCM$_O$. The energy of each state is also given in eV and the oscillator strength of the singlet states in indicated as "f".

	Singlet	0	1	2	3	4	5	6	7	8	9	10
	energy	0.00	2.14	3.08	3.12	3.20	3.53	3.63	3.70	3.85	3.89	4.06
	f	0.00	1.49	0.43	0.00	0.22	0.00	0.01	0.02	0.00	0.00	0.04
Triplet	energy											
1	1.32	1.25	0.10	0.64	19.72	0.71	63.36	0.80	0.51	0.08	7.27	0.46
2	2.34	1.05	0.23	0.12	10.58	0.40	19.52	0.80	1.14	0.17	5.21	1.30
3	2.56	5.19	0.32	0.32	1.08	0.17	57.20	1.59	1.01	0.06	9.13	1.25
4	2.94	26.06	17.61	0.38	0.02	6.01	0.27	4.46	11.52	2.20	0.12	2.75
5	3.05	9.22	0.49	0.63	13.26	1.13	11.92	0.32	1.27	0.16	1.89	4.41
6	3.26	5.46	0.47	0.95	2.92	1.53	87.63	1.85	1.68	0.06	14.88	2.69
7	3.32	3.62	0.14	0.43	1.89	0.96	28.40	1.23	0.57	0.04	3.39	1.86
8	3.47	34.53	2.56	4.23	3.72	2.92	164.12	12.92	2.13	0.15	22.09	2.06
9	3.50	520.43	37.32	89.39	0.37	60.40	13.13	166.50	36.19	2.43	49.57	4.73
10	3.65	5.53	0.12	0.91	2.15	0.97	50.89	0.28	0.74	0.03	8.61	0.85

Table S 11 Square modulus of spin-orbit coupling matrix elements (in cm^{-1}; bold font) between singlet and triplet states of I-DCM$_O$-Cl. The energy of each state is also given in eV and the oscillator strength of the singlet states in indicated as "f".

	Singlet	0	1	2	3	4	5	6	7	8	9	10
	energy	0.00	2.14	3.10	3.19	3.30	3.60	3.63	3.71	3.73	3.97	4.05
	f	0.00	1.47	0.40	0.26	0.00	0.00	0.00	0.01	0.01	0.00	0.04
Triplet	energy											
1	1.37	0.99	0.11	0.45	0.80	17.09	2.46	66.82	0.93	0.19	7.82	0.91
2	2.32	1.94	0.16	0.09	0.45	8.72	0.86	11.92	0.73	1.03	4.19	2.86
3	2.60	4.73	0.38	0.26	0.20	1.00	2.08	54.14	1.45	1.13	9.65	1.86
4	3.02	9.54	0.46	1.01	0.82	10.31	0.45	5.80	0.34	1.65	2.06	9.50
5	3.13	24.82	15.41	1.63	4.15	0.04	7.60	0.47	10.74	10.16	0.16	3.72
6	3.23	2.93	0.31	1.15	0.99	7.07	2.43	52.43	0.98	0.82	10.12	3.16
7	3.26	3.39	0.25	1.32	1.22	8.37	2.11	28.50	0.69	0.84	7.70	3.50

8	3.48	12.63	0.88	0.83	1.00	5.81	4.18	130.38	4.32	2.35	18.90	3.01
9	3.56	34.34	2.27	2.34	3.45	7.47	2.83	129.97	8.68	5.85	19.52	2.97
10	3.60	584.88	42.30	58.28	87.08	0.52	45.80	10.01	131.18	103.87	47.72	6.28

6. NMR Spectra

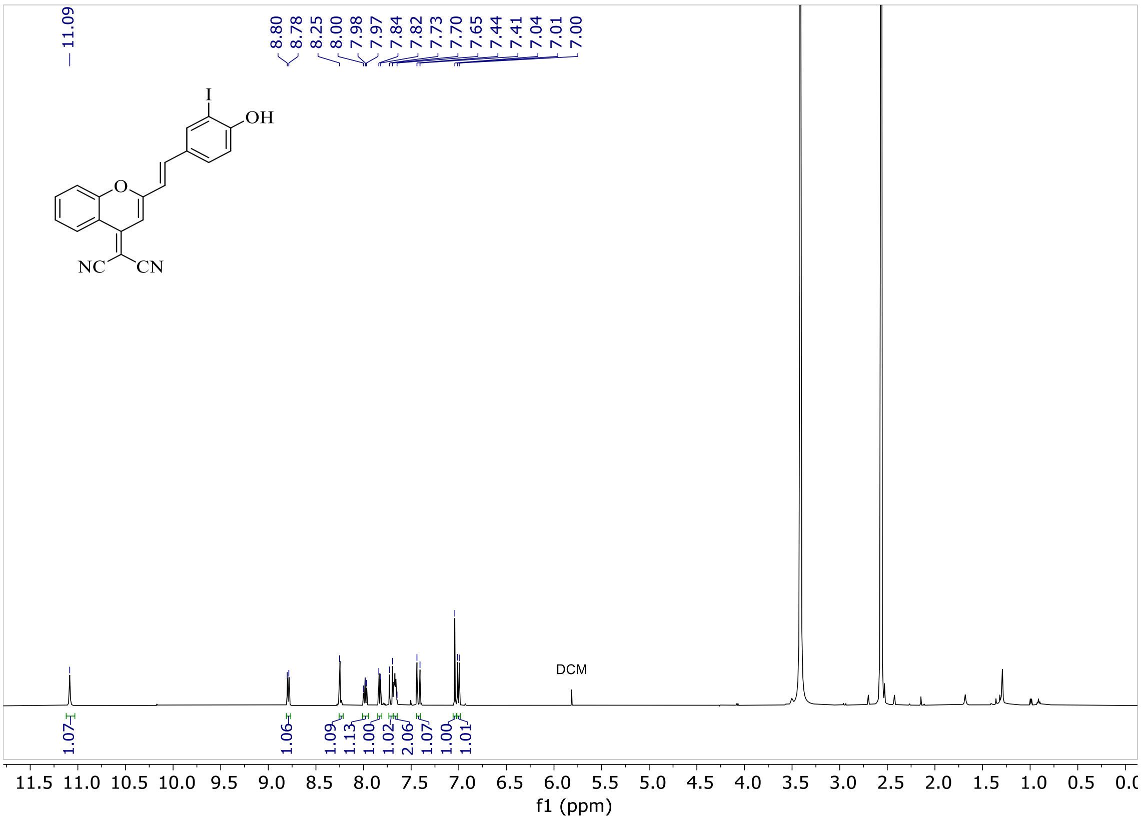

Figure S24 ^{1}H NMR spectrum of DCM_{O}- I in d_6-DMSO.

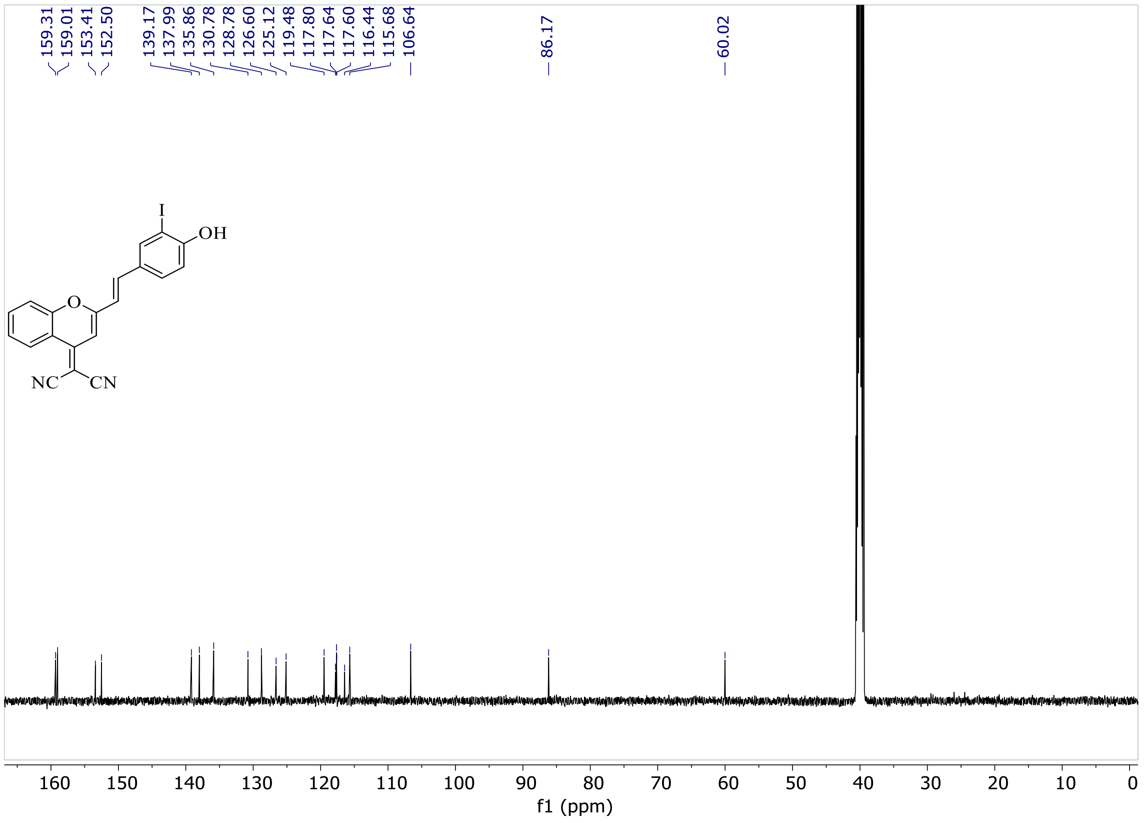

Figure S25 ^{13}C NMR spectrum of DCM$_O$- I in d_6-DMSO.

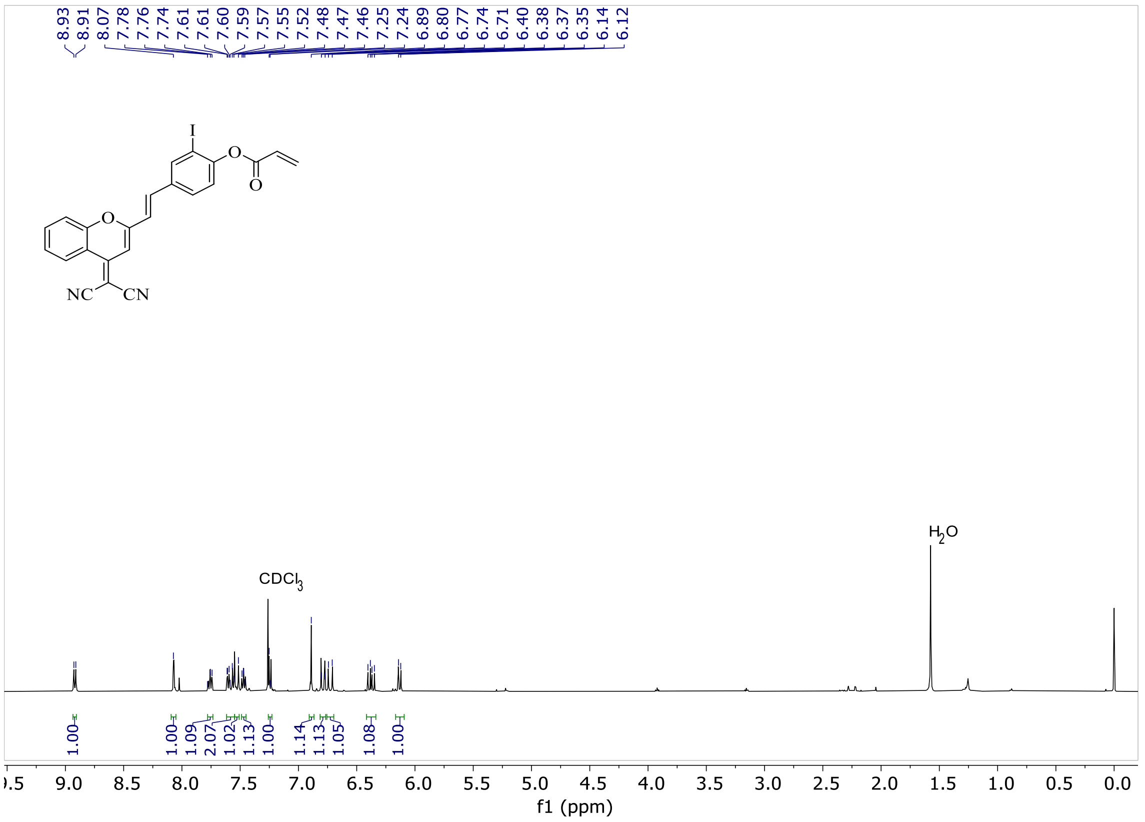

Figure S26 ^1H NMR spectrum of DCM$_O$-I-Cys in $CDCl_3$.

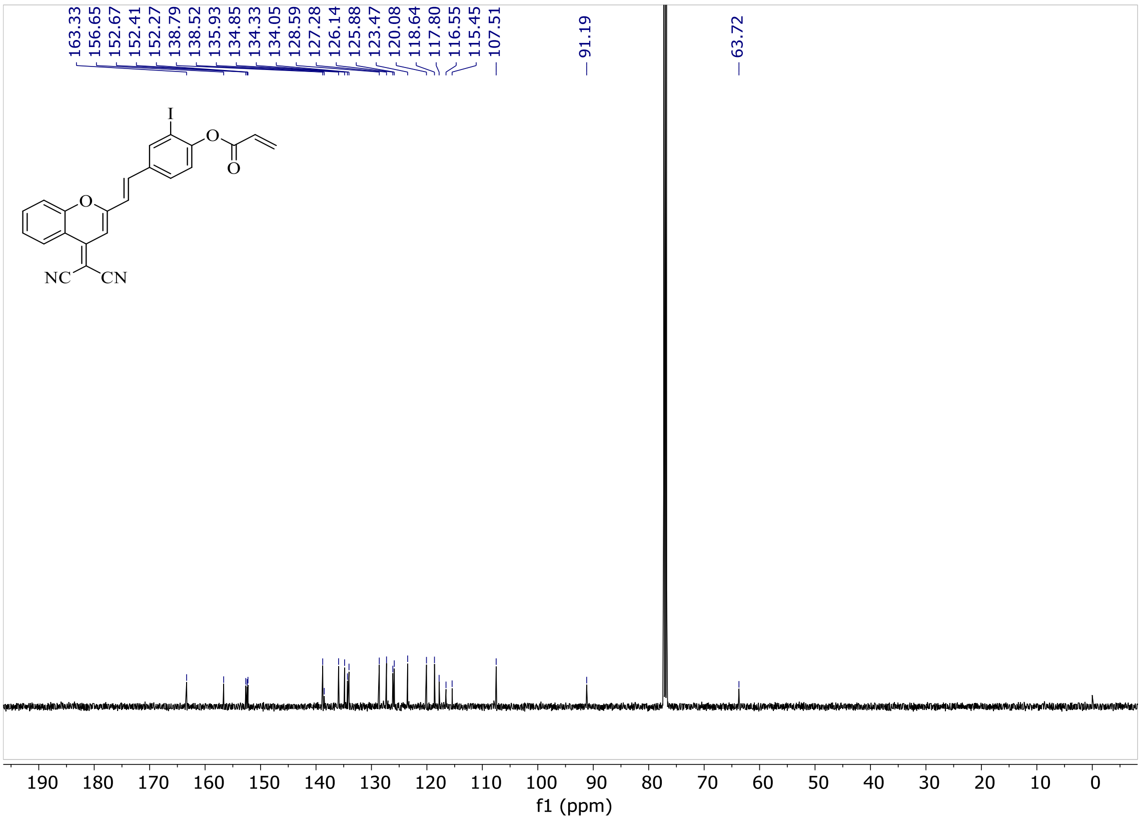

Figure S27 ^{13}C NMR spectrum of DCM_O-I-Cys in $CDCl_3$.

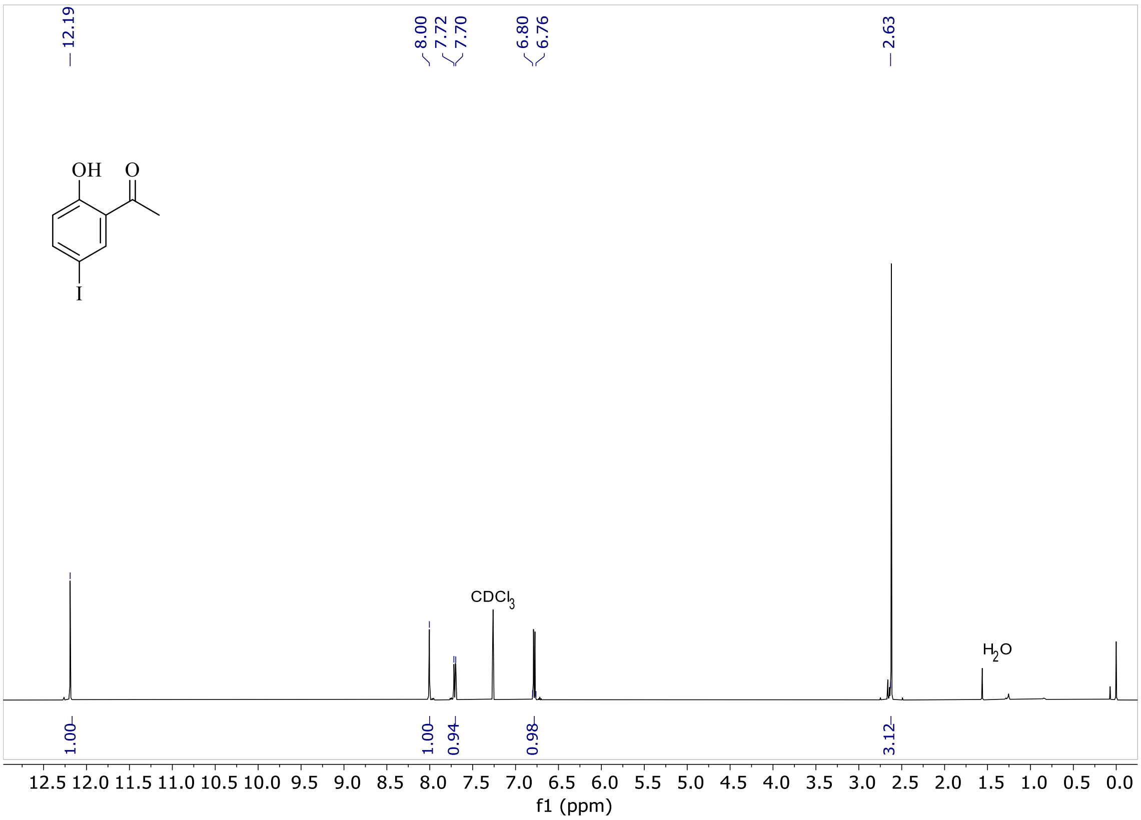

Figure S28 ^{1}H NMR spectrum of 3 in $CDCl_3$.

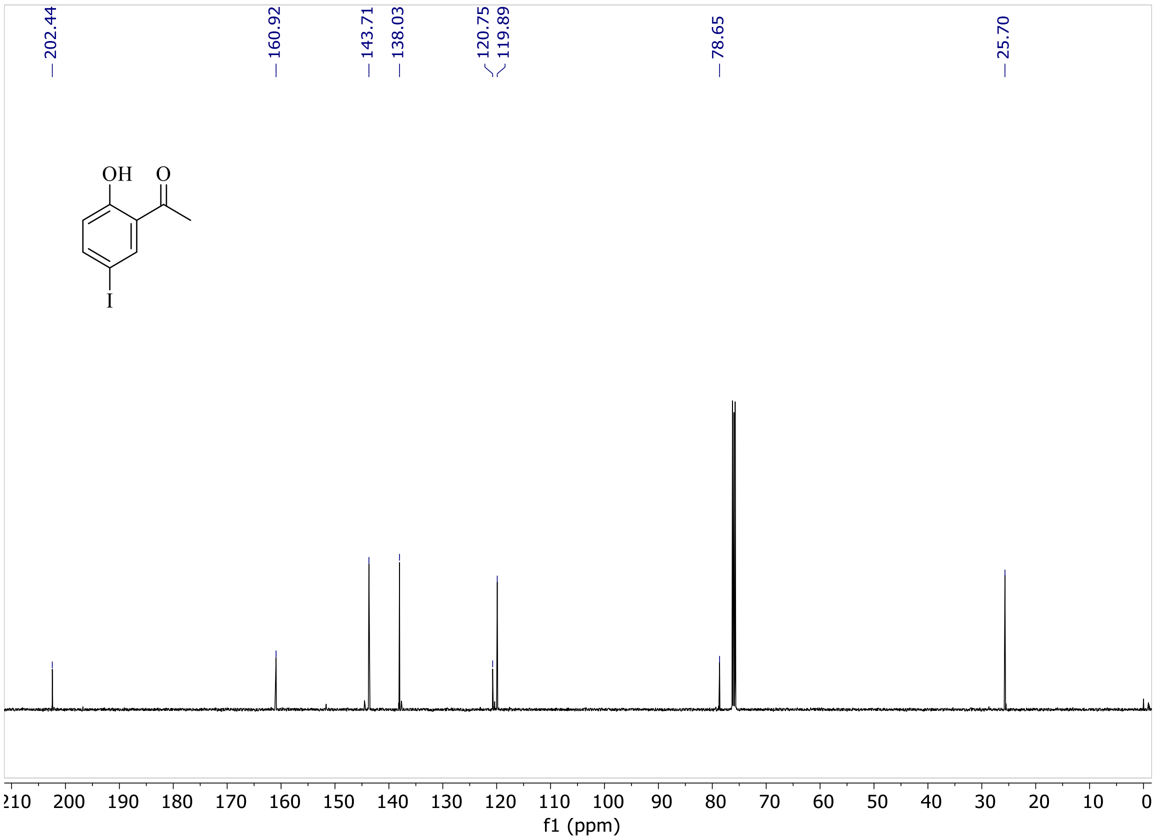

Figure S29 ^{13}C NMR spectrum of 3 in $CDCl_3$.

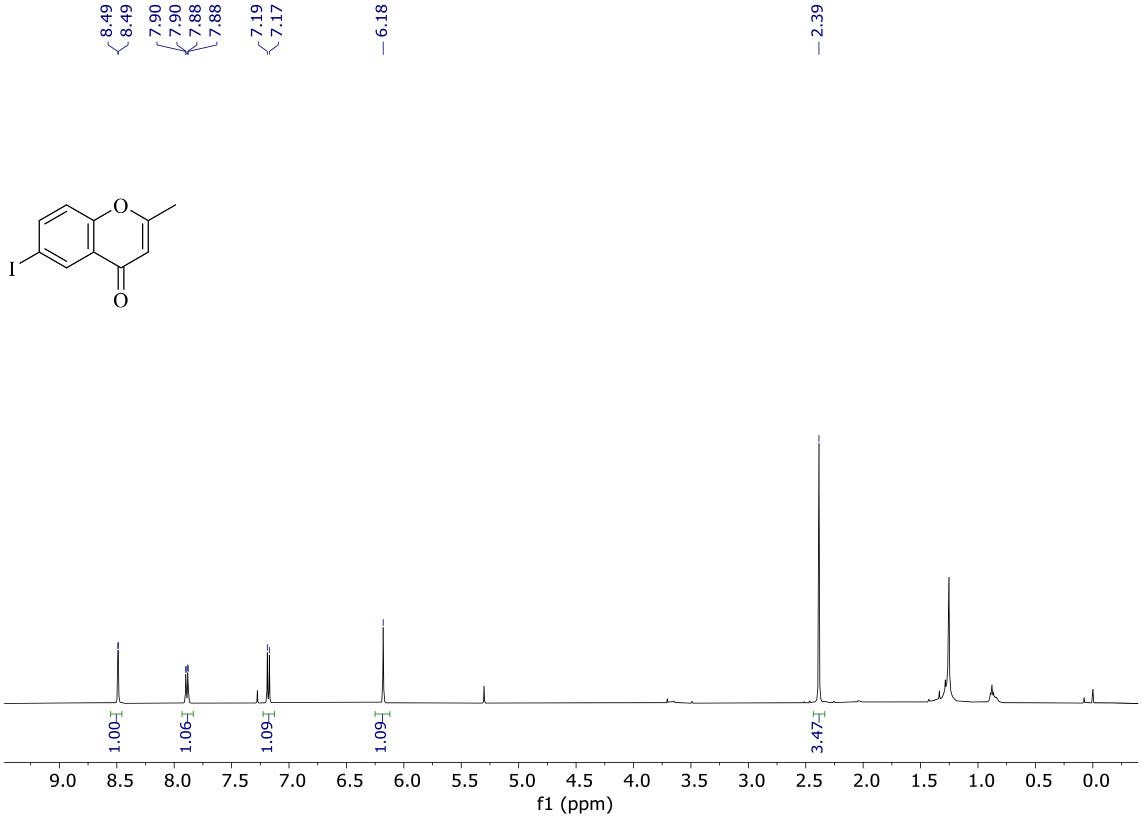

Figure S30 ^{1}H NMR spectrum of 4 in $CDCl_3$.

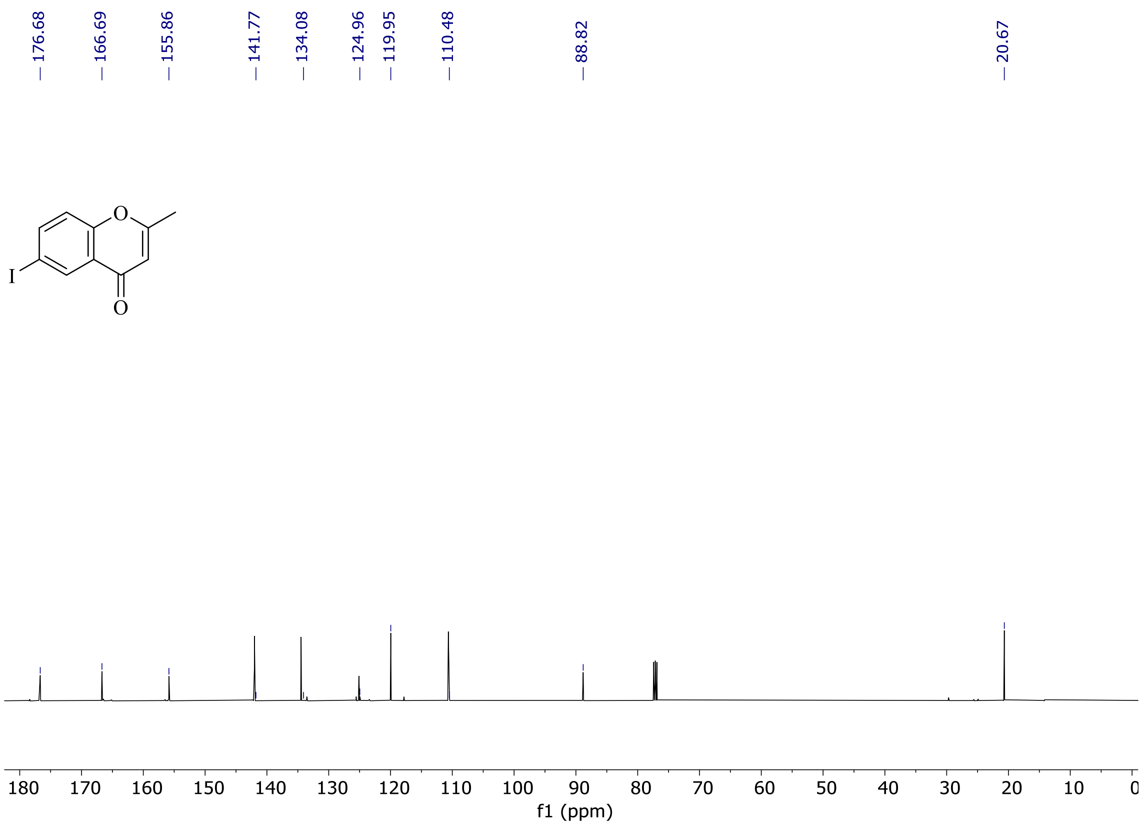

Figure S31 ^{13}C NMR spectrum of 4 in $CDCl_3$.

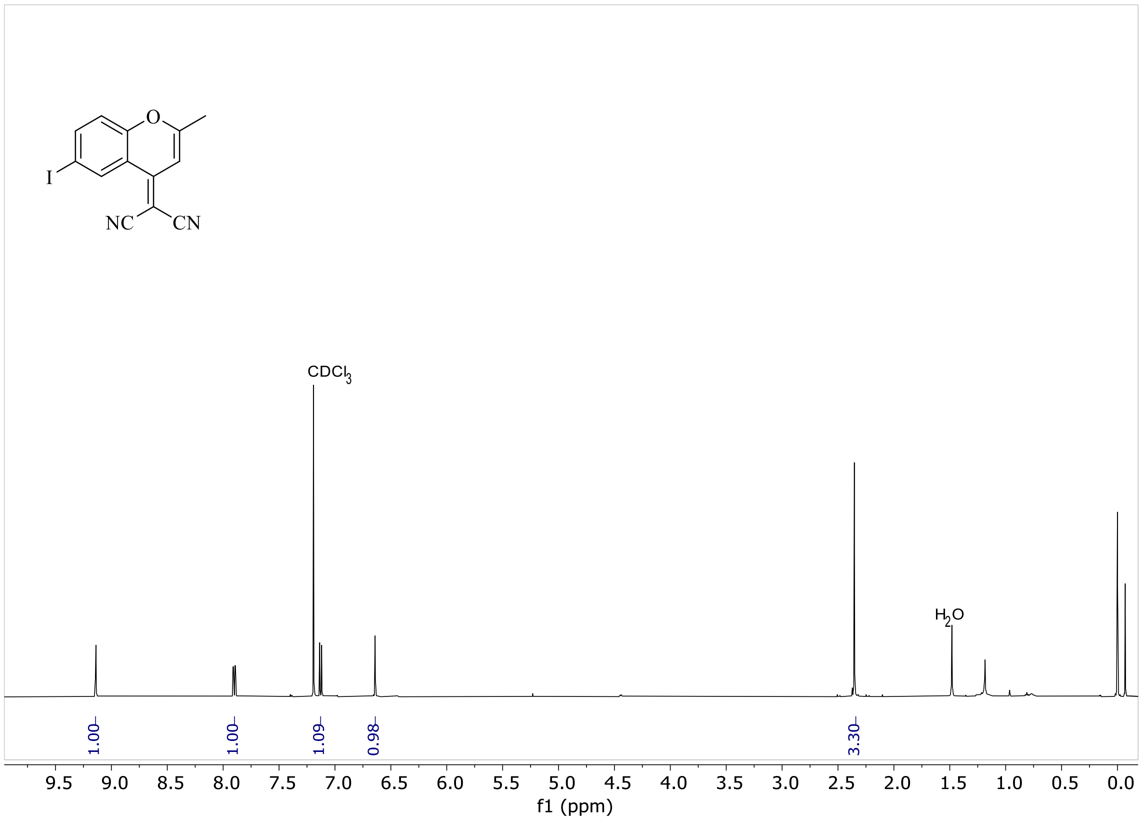

Figure S32 ^{1}H NMR spectrum of 5 in $CDCl_3$.

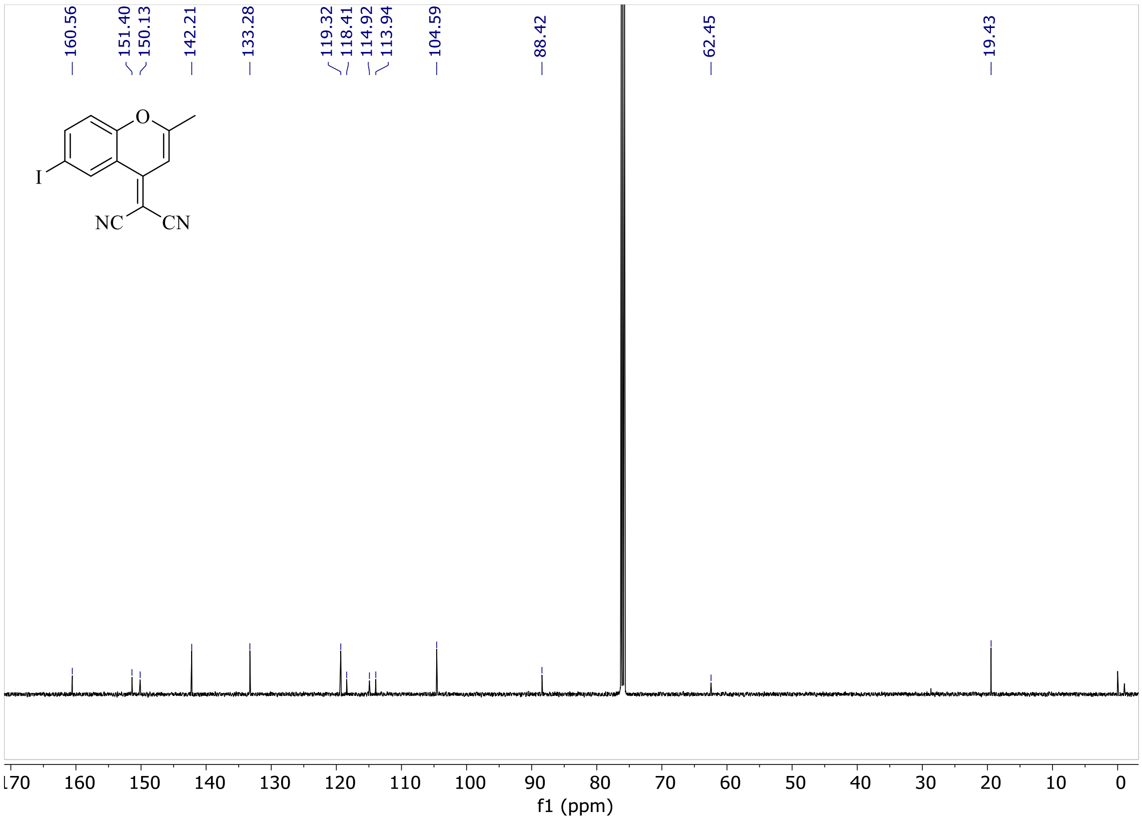

Figure S33 ^{13}C NMR spectrum of 5 in $CDCl_3$.

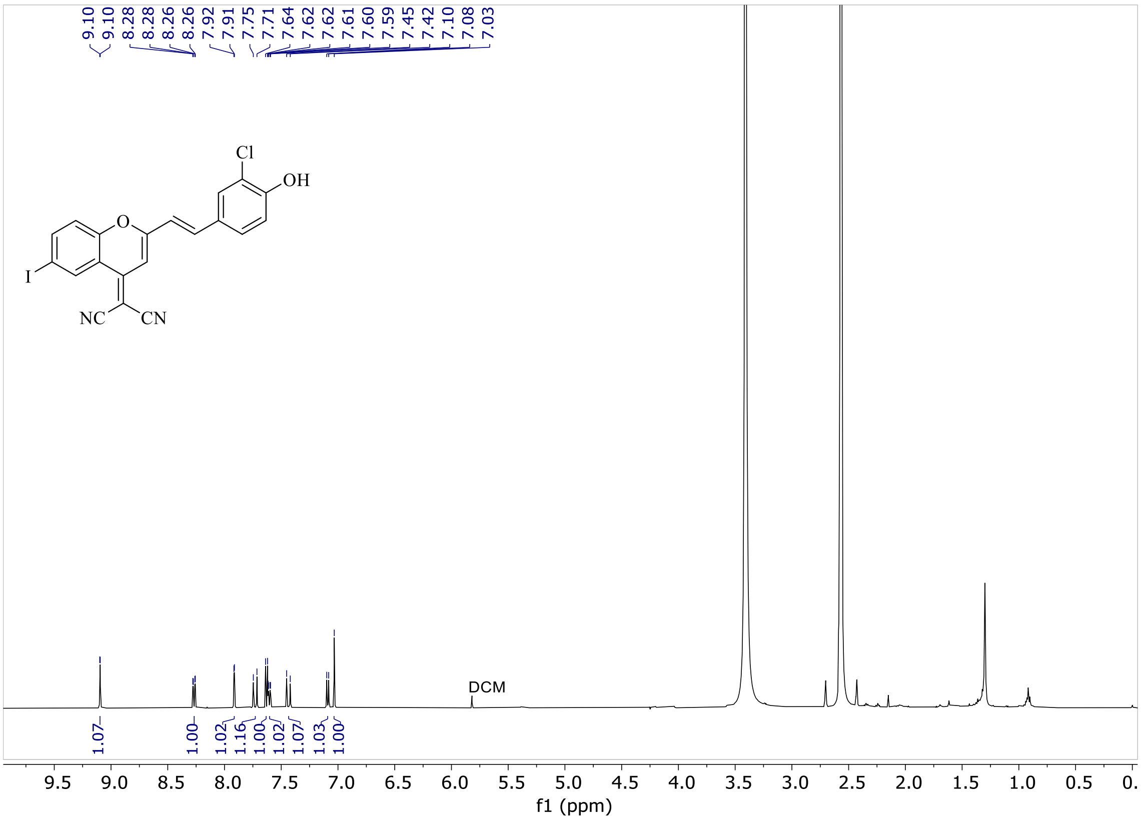

Figure S34 ^{1}H NMR spectrum of I-DCM$_{O}$-Cl in d$_6$-DMSO.

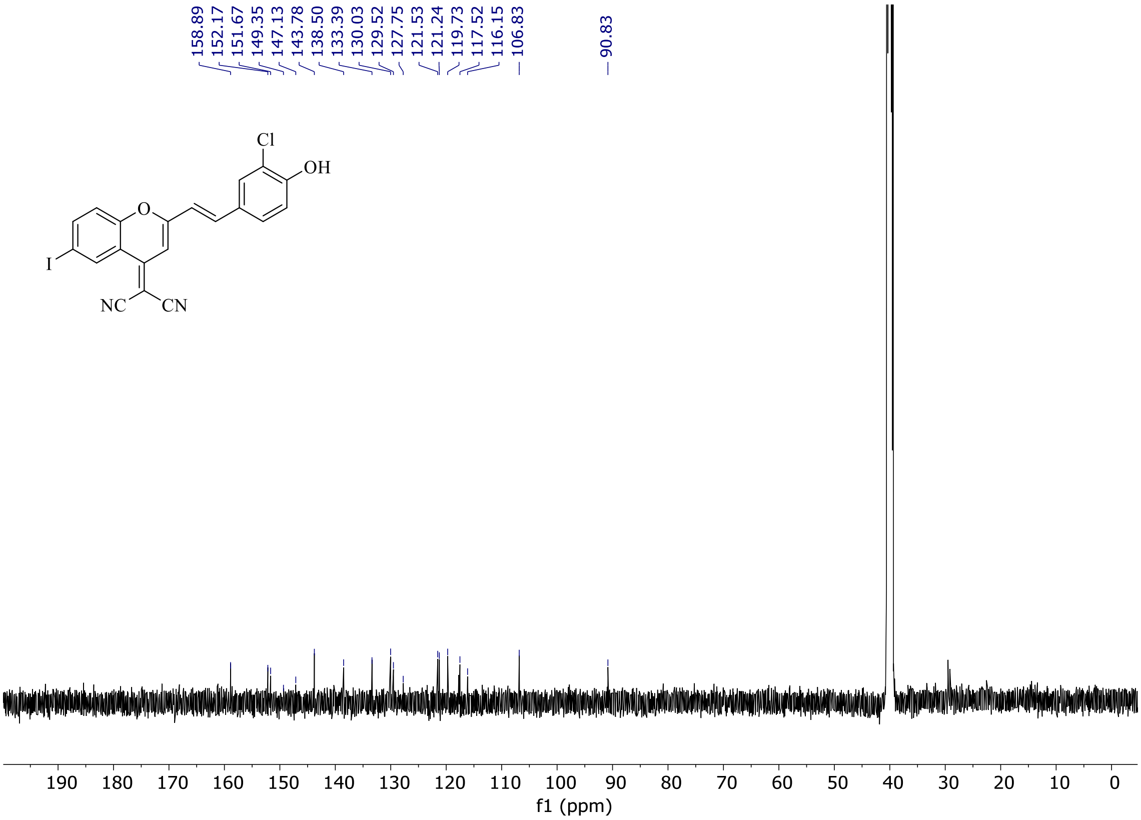

Figure S35 ^{13}C NMR spectrum of I-DCM$_{O}$-Cl in d$_6$-DMSO.

7. HR-MS Spectra:

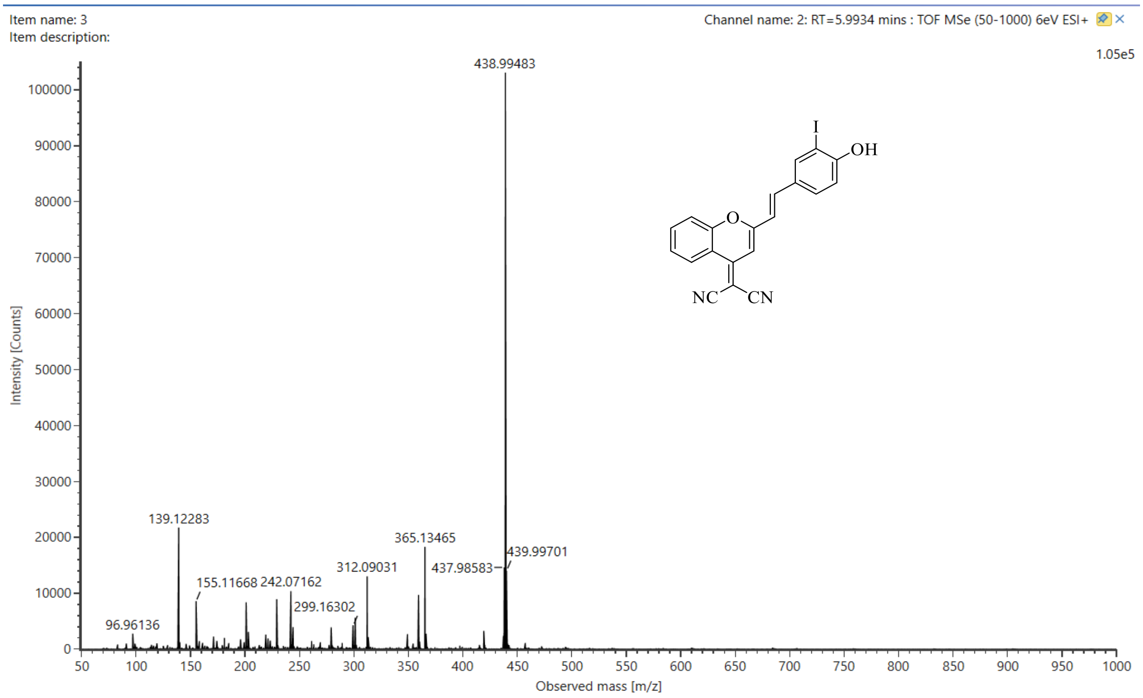

Figure S36 HRMS spectrum of DCM_O-I.

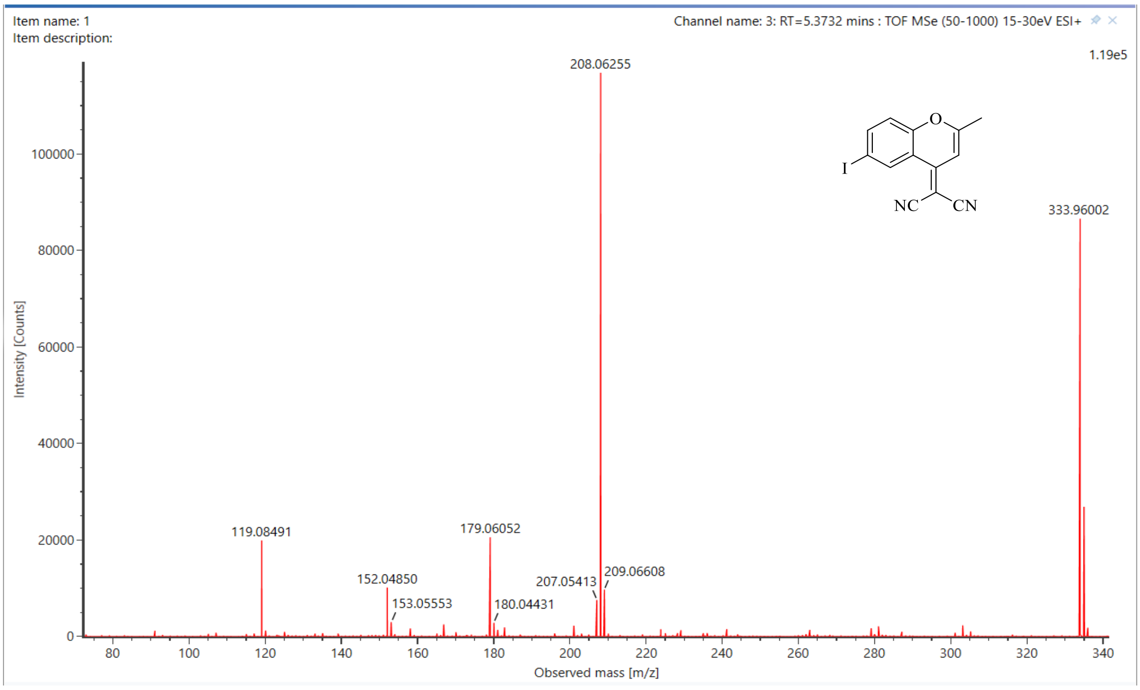

Figure S37 HRMS spectrum of Compound 5.

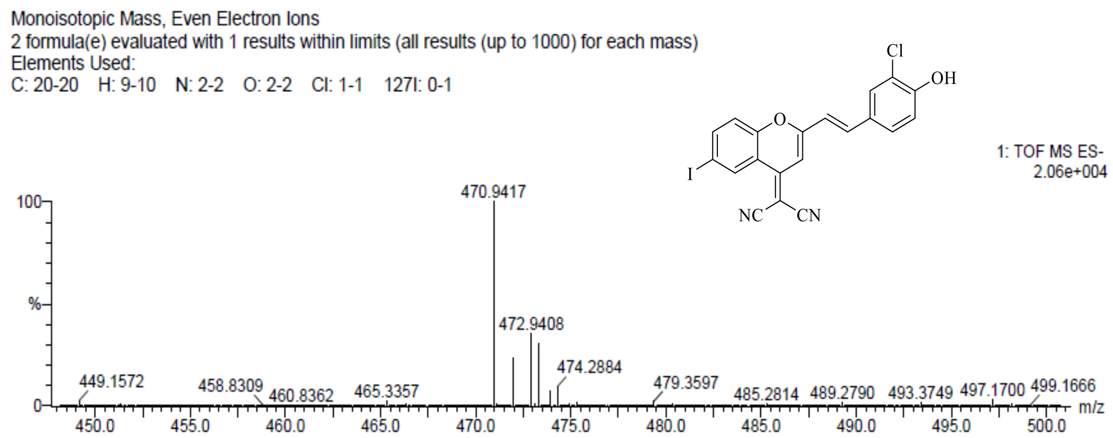

Minimum:				-5.5			
Maximum:		1000.0	1000.0	1000.0			
Mass	Calc. Mass	mDa	PPM	DBE	i-FIT	i-FIT (Norm)	Formula
470.9417	470.9397	2.0	4.2	16.5	228.0	0.0	C20 H9 N2 O2 Cl 127I

Figure S38 HRMS spectrum of I-DCM$_O$-Cl.

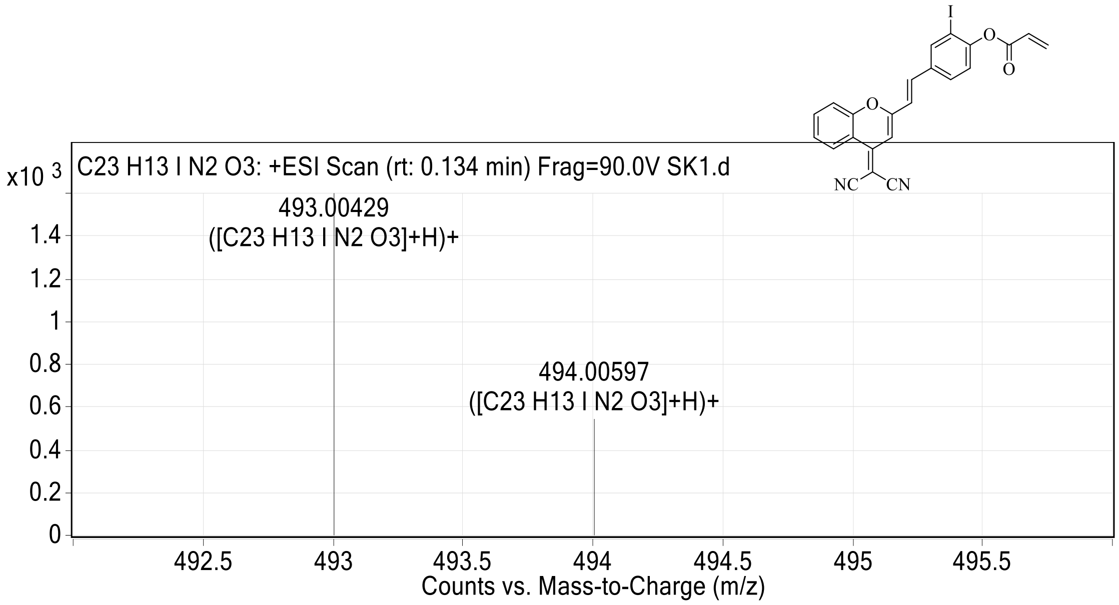

Figure S39 HRMS spectrum of DCM$_O$-I-Cys.

References

(1) Gu, K.; Xu, Y.; Li, H.; Guo, Z.; Zhu, S.; Zhu, S.; Shi, P.; James, T. D.; Tian, H.; Zhu, W. H. Real-Time Tracking and in Vivo Visualization of β-Galactosidase Activity in Colorectal Tumor with a Ratiometric Near-Infrared Fluorescent Probe. *J. Am. Chem. Soc.* **2016**, *138* (16), 5334–5340. https://doi.org/10.1021/jacs.6b01705.

(2) Shi, C.; Guo, Z.; Yan, Y.; Zhu, S.; Xie, Y.; Zhao, Y. S.; Zhu, W.; Tian, H. Self-Assembly Solid-State Enhanced Red Emission of Quinolinemalononitrile: Optical Waveguides and Stimuli Response. *ACS Appl. Mater. Interfaces* **2012**, *5* (1), 192–198. https://doi.org/10.1021/am302466m.

(3) Lutkus, L. V.; Rickenbach, S. S.; McCormick, T. M. Singlet Oxygen Quantum Yields Determined by Oxygen Consumption. *J. Photochem. Photobiol. A Chem.* **2019**, *378*, 131–135. https://doi.org/10.1016/j.jphotochem.2019.04.029.
